# Synthesis of New Chiral *β*-Carbonyl Selenides with Antioxidant and Anticancer Activity Evaluation—Part I

**DOI:** 10.3390/ma17040899

**Published:** 2024-02-15

**Authors:** Anna Laskowska, Agata J. Pacuła-Miszewska, Magdalena Obieziurska-Fabisiak, Aneta Jastrzębska, Angelika Długosz-Pokorska, Katarzyna Gach-Janczak, Jacek Ścianowski

**Affiliations:** 1 Department of Organic Chemistry, Faculty of Chemistry, Nicolaus Copernicus University, 7 Gagarin Street, 87-100 Torun, Poland; annlas@doktorant.umk.pl (A.L.); pacula@umk.pl (A.J.P.-M.); magdao@umk.pl (M.O.-F.); 2 Department of Analytical Chemistry and Applied Spectroscopy, Faculty of Chemistry, Nicolaus Copernicus University, 7 Gagarin Street, 87-100 Torun, Poland; aj@umk.pl; 3 Department of Biomolecular Chemistry, Faculty of Medicine, Medical University of Lodz, Mazowiecka 6/8, 92-215 Lodz, Poland; angelika.dlugosz@umed.lodz.pl (A.D.-P.); katarzyna.gach@umed.lodz.pl (K.G.-J.)

**Keywords:** 2-((2-oxopropyl)selanyl)benzamides, pharmacophore, antioxidant activity, antiproliferative activity

## Abstract

A series of unsymmetrical phenyl *β*-carbonyl selenides with *o*-amido function substituted on the nitrogen atom with chiral alkyl groups was obtained. The compounds form a series of enantiomeric and diastereomeric pairs and present the first examples of this type of chiral Se derivatives. All obtained selenides were further evaluated as antioxidants and anticancer agents to define the influence of the particular stereochemistry of the attached functional groups on the bioactivity of the molecules. The highest H_2_O_2_ reduction potential was observed for *N*-(*cis*-2-hydroxy-1-indanyl)-2-((2-oxopropyl)selanyl)benzamide, and the best radical scavenging properties for *N*-(-1-hydroxy-2-butanyl)-2-((2-oxopropyl)selanyl)benzamide. Also, both enantiomers of the *N*-(1-hydroxy-2-butanyl) selenide expressed the highest cytotoxic potential towards human promyelocytic leukemia HL-60 cell line with similar IC_50_ values 14.4 ± 0.5 and 16.2 ± 1.1 µM, respectively. On the other hand, breast cancer cell line MCF-7 was most sensitive to *N*-((*R*)-(-)-1-hydroxy-2-butanyl)- 2-((2-oxopropyl)selanyl)benzamide (IC50 of 35.7 ± 0.6 µM). The structure–activity dependence of the obtained Se derivatives was discussed, and the most potent compounds were selected.

## 1. Introduction

Finding relationships between the chemical structure and biological activity is an approach that enables the proper design and further selection of potential drug candidates. Various structural features of the evaluated compounds have to be considered, including the installation of functional groups that influence the physicochemical properties of the molecule, the bonding arrangement of the compound’s basic skeleton, and its chirality. The selection and incorporation of pharmacophores that can interact and fit into specific domains of the receptor and improve the molecule’s bioavailability allow the potential drug’s target bioproperty to be achieved [1,2,3]. In this context, organoselenium compounds, creating a diversified group of derivatives with remarkable redox properties, are constantly being modified and evaluated to find the perfect combination of structural features to maximize their biocapacity and toxicity reduction [4,5,6].

**Figure 1:**
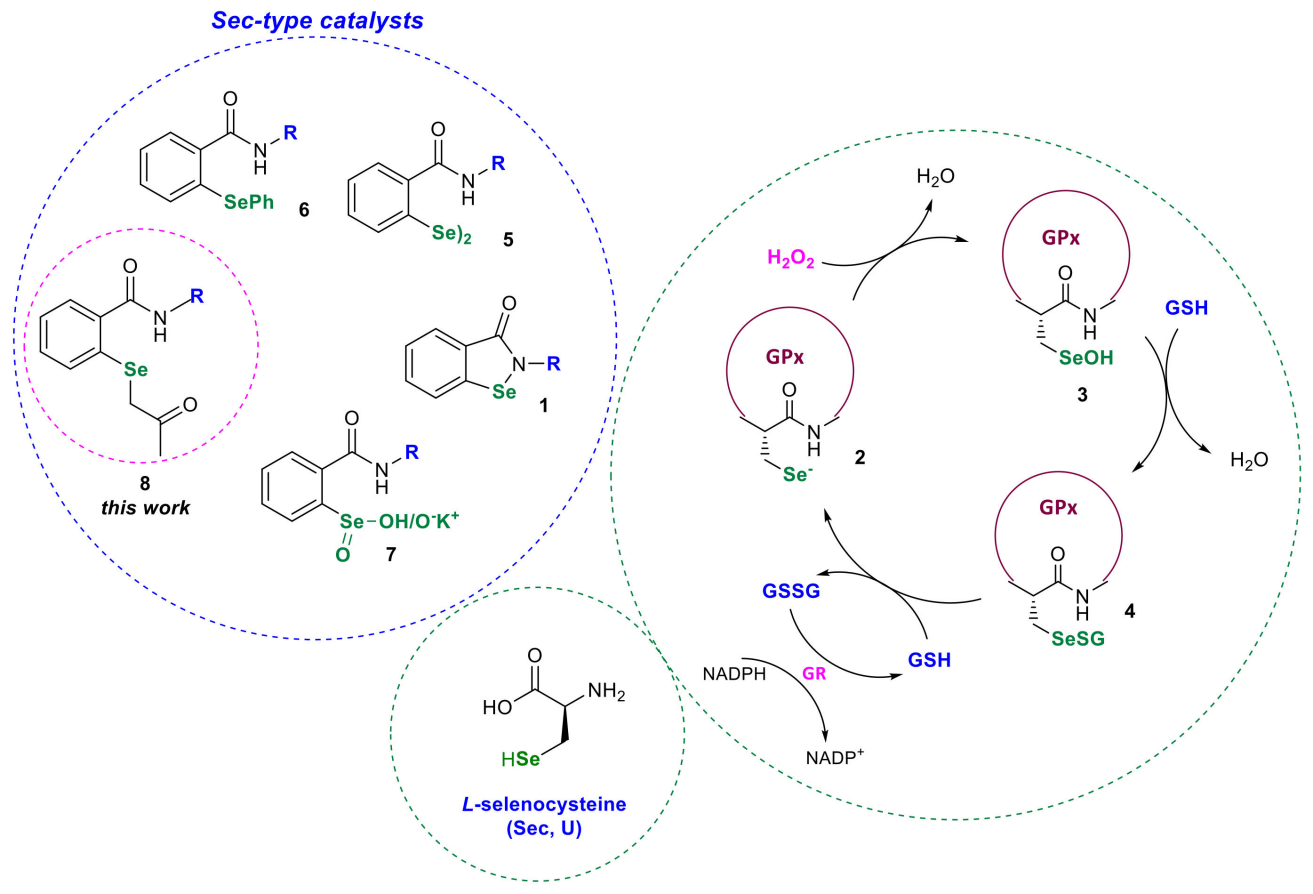
GPx activity cycle and examples of Sec-type catalysts **1**, **5**–**8**.

The importance and applicability of Se compounds have been highlighted in various research fields, including catalysis [7], material science [8], and medicinal chemistry [9,10]. Due to the high reactivity of the selenium atom and facile introduction into the structure of the substrate, through selenenylation and selenocyclization reactions [11], new Se derivatives can be efficiently obtained, providing a broad range of diversified heterorganic compounds under mild reaction conditions. Among this group of derivatives, *N*-substituted benzisoselenazolones **1** occupy an important place because of their unique ability to act as an artificial L-selenocysteine-like catalyst and mimic the activity of the antioxidant selenoenzyme glutathione peroxidase (GPx) [12]. It should be highlighted that the family of glutathione peroxidase includes eight isozymes and five of them are selenocysteine-containing proteins. Some of them are being studied with cancerous diseases. But, on the other hand, different GPx subtypes play different roles in tumors, and the mechanisms of action need to be studied further [13,14,15,16]. In the presented catalytic cycle of H_2_O_2_ reduction by GPx, the active selenol of L-Sec **2**, maintained in its ionized form through a hydrogen bond network with the neighboring amino acid environment (glutamine and tryptophan residues [17]) is first oxidized to the selenenic acid **3**, and further regenerated in the presence of two GHS molecules. The spatial structure of the potential GPx mimetic, which needs to interact with the homochiral L-amino acid residues of the enzyme, influences the efficiency of the entire biochemical cycle. In our research, we synthesized and evaluated the antioxidant and anticancer properties of various *N*-substituted benzisoselenazolones, including chiral *N*-terpenyl and aminoacid derivatives [18]. We have additionally explored the possibility of improving their catalytic properties by converting the Se-N bond into different functional groups that could also imitate the active selenol of L-Sec, like corresponding diselenides **5** [19], phenylselenides **6** [20], seleninic acids, and their potassium salts **7** [21]. Herein, we have further differentiated the primary structure by synthesizing a series of new *N*-functionalized *β*-carbonyl selenides **8** (Figure 1).

The synthesis of this type of selenide **8** in the form of *N*-chiral enantiomeric and diastereomeric pairs will enable us to conclude the influence of the configuration of particular carbon centers on the catalytic and antiproliferative activity of the molecules. Additionally, the ability of the 2-(2-oxopropyl)selanyl moiety to mimic the selenocysteine selenolate anion and the influence of an additional carbonyl moiety will be evaluated.

## 2. Results and Discussion

The first step of this research involved synthesizing *N*-substituted 2-((2-oxopropyl)selanyl)-benzamide derivatives. The first example of this type of compound, derived from D-glucosamine, was obtained by Z. Zhang and co-workers in 2010 [22]. The reaction of 2-(chloroseleno)benzoyl chloride with 1,3,4,6-tetra-O-acetyl-2-deoxy-b-D-glucopyranosylamine, performed in acetone and catalyzed by sodium bicarbonate, gave the final product with 60% yield. Then, Sh. Zhang et al. obtained a series of *β*-carbonyl selenides through a modified procedure using dichloromethane as the solvent and eliminating NaHCO_3_ [23,24]. Herein, we have optimized the reaction conditions based on the synthesis of selenide **9**. The method presented recently by Sh. Zhang and co-workers furnished the final product with only a 30% yield (entry 1). The addition of sodium bicarbonate slightly increased the efficiency of the procedure (entry 2). Using a 0.75 M solution of NaHCO_3_ (entries 3 and 4) also did not improve the reaction yield. The order in which the reagents were added significantly influenced the overall process. The reaction’s key step was mixing the ketone with sodium bicarbonate to generate the carbanion for the subsequent acylation of the selenium atom. The best result was obtained using an excess of acetone as both the substrate and the solvent, furnishing the final product with 87% yield (entry 6, Table 1).

**Scheme 1:**
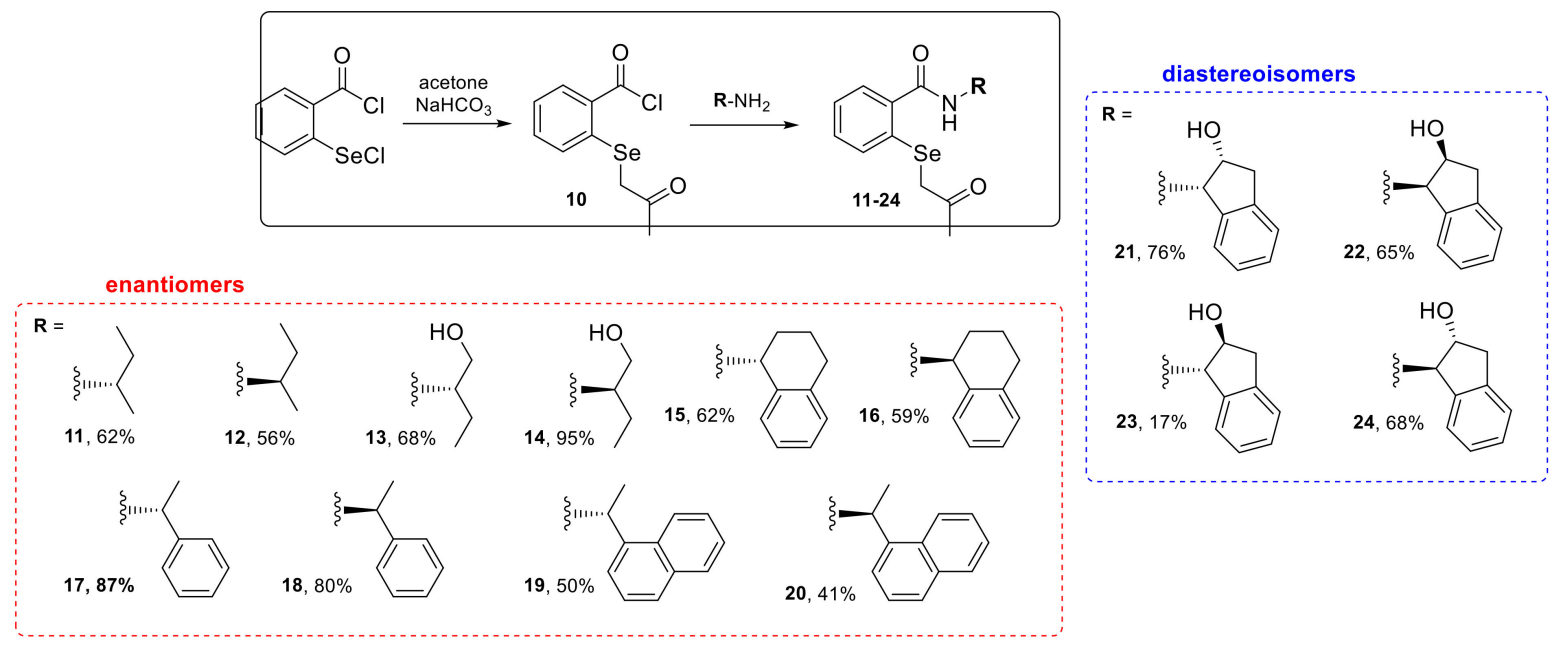
Synthetized chiral β-carbonyl selenides **11**–**24**.

The selected conditions proved efficient for a variety of chiral amines. In one-pot synthesis, 2-(chloroseleno)-benzoyl chloride was first transformed in situ to the acetylated selenide **10**. Subsequent reactions with commercially available enantiomerically pure amines furnished a series of *β*-carbonyl selenides as enantiomeric and diastereomeric pairs in good yields (Scheme 1).

The ability to reduce hydrogen peroxide was evaluated by a conventionally used assay presented by Iwaoka and co-workers [25]. The method is based on the oxidation of the Se catalyst to its active species **25** by H_2_O_2_, which is later reduced to its initial form by dithiothreitol (DTT^red^). The dithiol DTT is simultaneously transformed into a disulfide (DTT^ox^). ^1^HNMR spectra, recorded in specific time intervals, present the rate of the dithiol formation, which equals the rate of H_2_O_2_ reduction. The results of the performed analysis are shown in Table 2.

**Figure 2:**
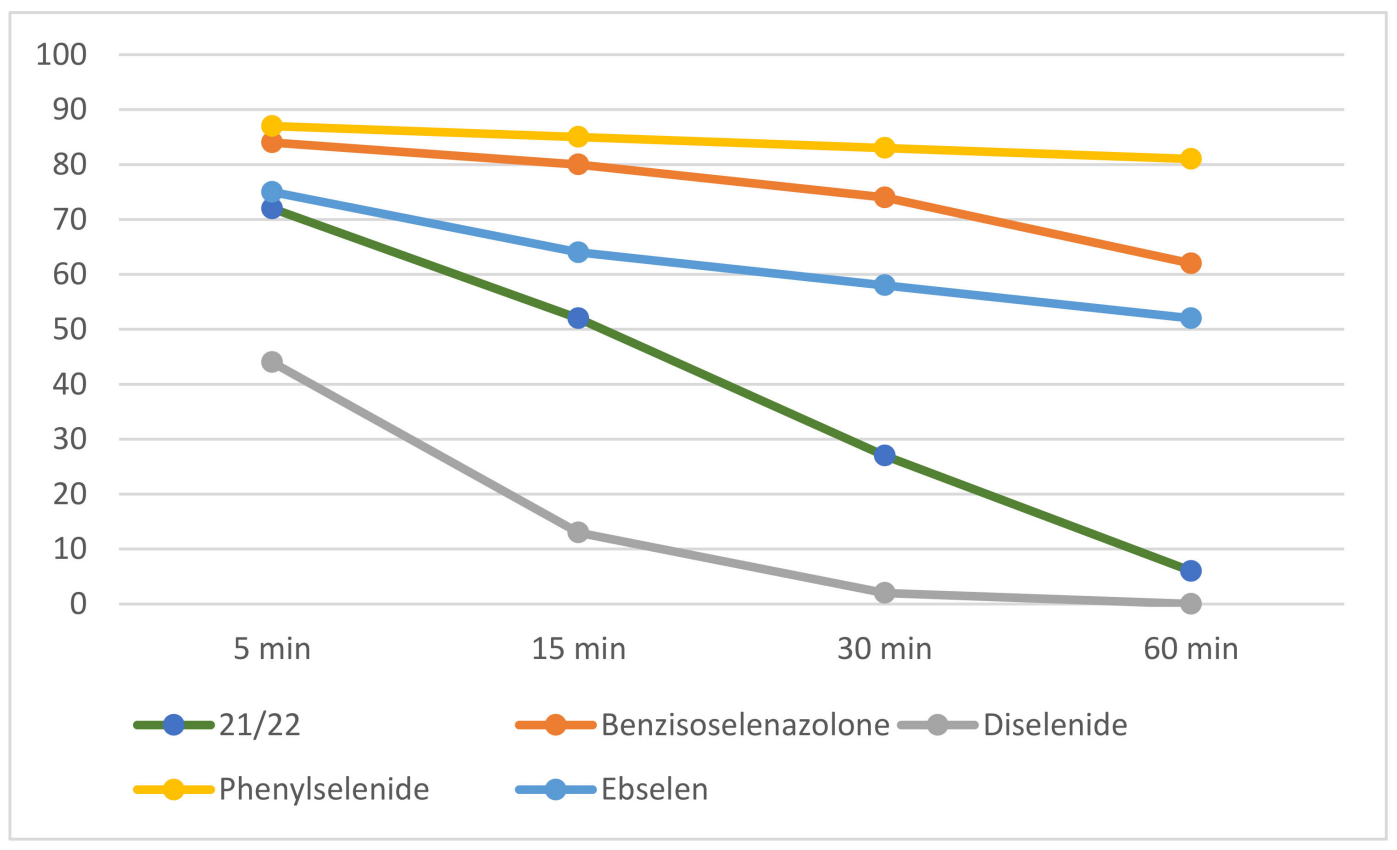
Results of the antioxidant activity measurements of corresponding Se derivatives.

All enantiomeric and diastereomeric pairs presented the same H_2_O_2_-scavenging potential. In general, the highest reactivity was observed for both *N*-indanyl selenides possessing the 2-hydroxy group in *cis* (**21**/**22**) and *trans* (**23**/**24**) configuration, with the DTT^red^ reduced to 6 and 10%, respectively. The best result obtained for compounds **21**/**22** was compared to the values measured for corresponding *N*-(*cis*-2-hydroxy-1-indanyl) Se derivatives. As presented in Figure 2, it can be observed that diselenides possessing a reactive and susceptible to facile cleavage Se-Se bond are the most efficient H_2_O_2_ scavengers. However, the presence of a *β*-carbonyl selanyl group, instead of a phenylselanyl moiety or a Se-N bond of the benzisoselenazolone core, significantly enhanced the H_2_O_2_ reduction potential.

It also has to be noted that the sterically undeveloped *N*-(2-butyl) derivatives **11**/**12** and those possessing an additional hydroxy group **13**/**14** were 4 and 2,5 fold, respectively, more active than the well-known GPx-mimetic ebselen. The introduction of additional aromatic rings to the *N*-alkyl chains, without the attachment of any polar OH group, seems to reduce the H_2_O_2_ scavenging capacity (derivatives **15**/**16**, **17**/**18**, **19**/**20**, Table 2).

Next, the free radical scavenging activity of the tested selenides was evaluated using the 2,2-diphenyl-1-picrylhydrazyl (DPPH) test (Table 3). This straightforward procedure relies on donating electrons from the tested compounds to neutralize the radical, changing the color of DPPH from purple to yellow [26,27]. The discussed assay depends on a mixed mode involving mechanisms such as hydrogen atom transfer, single electron transfer, and proton-coupled electron transfer [26]. The advantages of the DPPH test encompass low cost, effectiveness, experimental simplicity, reproducibility, applicability at ambient temperature, and the capability for result comparison with alternative radical scavenging methodologies [27,28,29]. In contrast to other free radicals, such as peroxyl radicals, hydroxyl radicals, or superoxide radical anions, the DPPH radical exhibits stability [27]. On the other hand, DPPH is capable of reacting under certain conditions, even in the absence of antioxidants, for example, in the presence of oxygen or sunlight [30]. Nevertheless, due to the usually short time of reaction between tested compounds and DPPH radical (less than 60 min), these reactions do not affect the final result of the analysis.

**Scheme 2:**
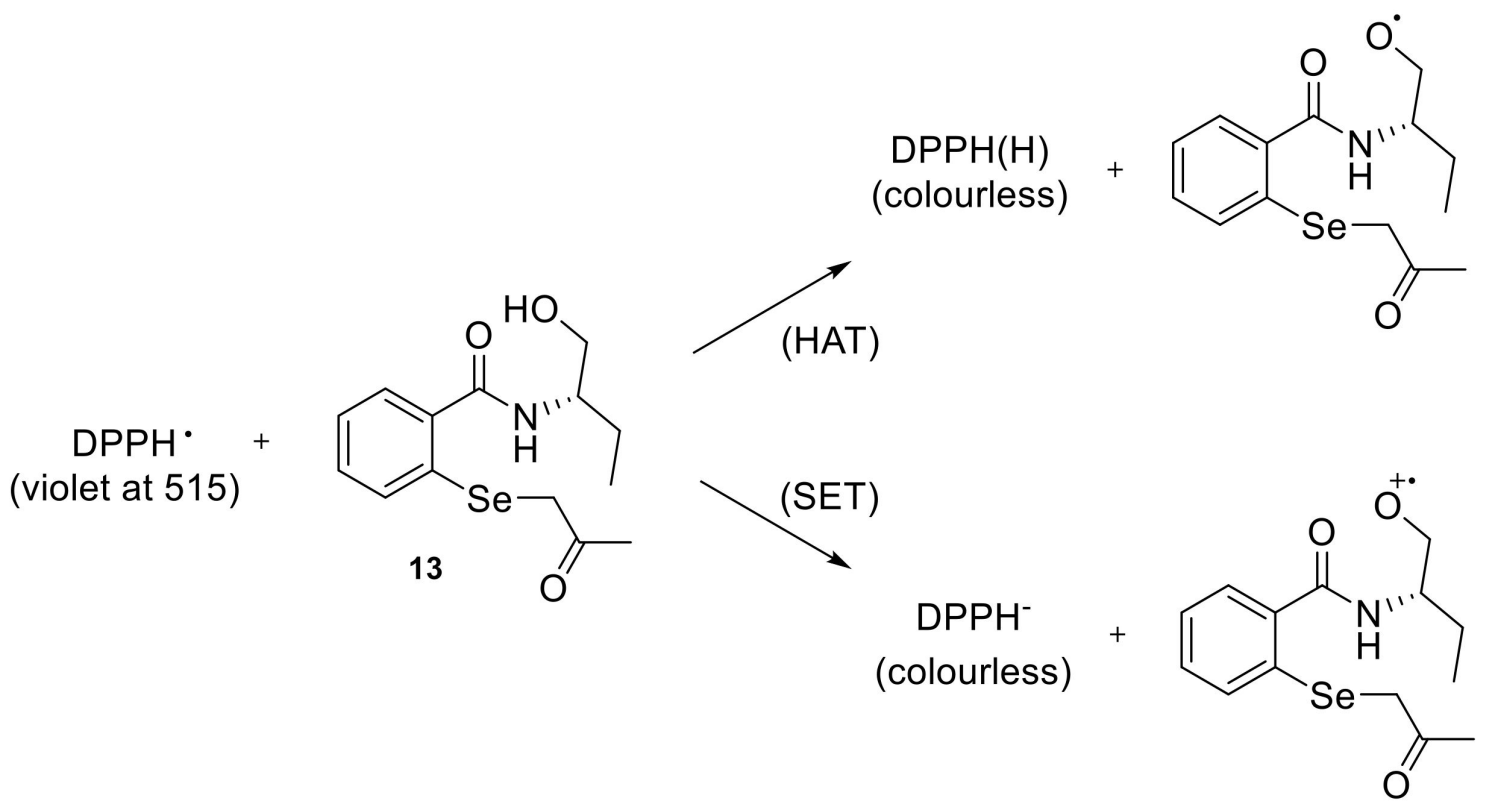
A plausible mechanism for the DPPH reduction, where HAT—hydrogen atom transfer; SET—single electron transfer.

All tested selenides demonstrated the neutralization of the DPPH radical, nevertheless, all calculated values were higher than the IC_50_ for Trolox (0.17 mM). The presence of different substituents affects their antioxidant activity. The scavenging capacity on DPPH radicals was in the order of **13**/**14** > **23**/**24** > **19**/**20** > **21**/**22** > **15**/**16** > **17**/**18** > **11**/**12**. Additionally, the Trolox equivalent antioxidant capacity (TEAC) was calculated [31,32]. The obtained results were as follows: 0.19 (for **13**/**14**), 0.18 (for **23**/**24**), 0.10 (for **19**/**20**), 0.07 (for **21**/**22**, **15**/**16**, and **17**/**18**), and 0.03 for **11**/**12**. The tested compounds share a heteroaromatic ring of high electronegativity, and they only differ in their *N*-substituted amido group, which comes from chiral amines or amino alcohols. Obtained results indicated that enantiomers **13**/**14** were the most potent free radical inhibitors, which react with free radicals, particularly the hydroperoxide radical. In the structure of **13**/**14**, the nitrogen atom is substituted with a chiral aliphatic butyl chain possessing an OH group. The latter has been recognized as a hydrogen donor to quench electron mobility while interrupting the free radical chain reaction. Based on the mechanisms of the DPPH assay described in the literature [26,27,33,34], we assume that the reaction can occur by a combination of hydrogen atoms and/or a single electron transfer mechanism, presented for compound **13** in Scheme 2.

Studies of the antioxidant role of *N*-substituted benzisoselenazolones and their derivatives are scarce compared to those in biological substrates. Moreover, the IC 50 parameter is a characteristic property of a given antioxidant only under certain conditions, so comparing the obtained results with those described in the literature is often difficult. We have observed different effects of substituents on the DPPH radical scavenging properties for the tested compounds. Regarding the antioxidant activity, additional insertion of the hydroxyl group contributes to the scavenging activities. However, from the obtained results, establishing correlations between the structure of *β*-carbonyl selenides and their DPPH neutralization proved challenging. It would be interesting to know the possible mechanisms of the antioxidant activity of aromatic and aliphatic substituents of the tested structures, typical to different potentially interesting molecules with antioxidant activity. Therefore, our future research will focus on this issue.

The antiproliferative activity of all Se compounds was measured using a cell viability assay (MTT) on breast cancer MCF-7 and human promyelocytic leukemia HL-60 cell lines [35]. In this case, the highest cytotoxic potential was observed for both enantiomers of *N*-(1-hydroxy-2-butanyl) selenide **13** and **14** with similar IC_50_ values—14.4 ± 0.5 and 16.2 ± 1.1 µM (HL-60 cell line), respectively (Table 4).

Comparing the results obtained for selenides **13** and **14** to the IC50 values measured for corresponding phenylselenides, benzisoselenazolones, and diselenides, substituted on the nitrogen atom with the same *N*-(1-hydroxy-2-butanyl) scaffolds, we can observe a structure–activity dependence relative to the type of attached Se moiety. In general, the difference in cytotoxic potential is more visible towards HL-60 cell lines. Selenides **13** and **14** are 2-fold more active than corresponding benzisoselenazolones and slightly less cytotoxic than the *N*-(1-hydroxy-2-butanyl) diselenides. Additionally, the exchange of the *β*-carbonyl group to a phenyl ring significantly decreases the antiproliferative activity of the Se-derivative (Table 5).

**Figure 3:**
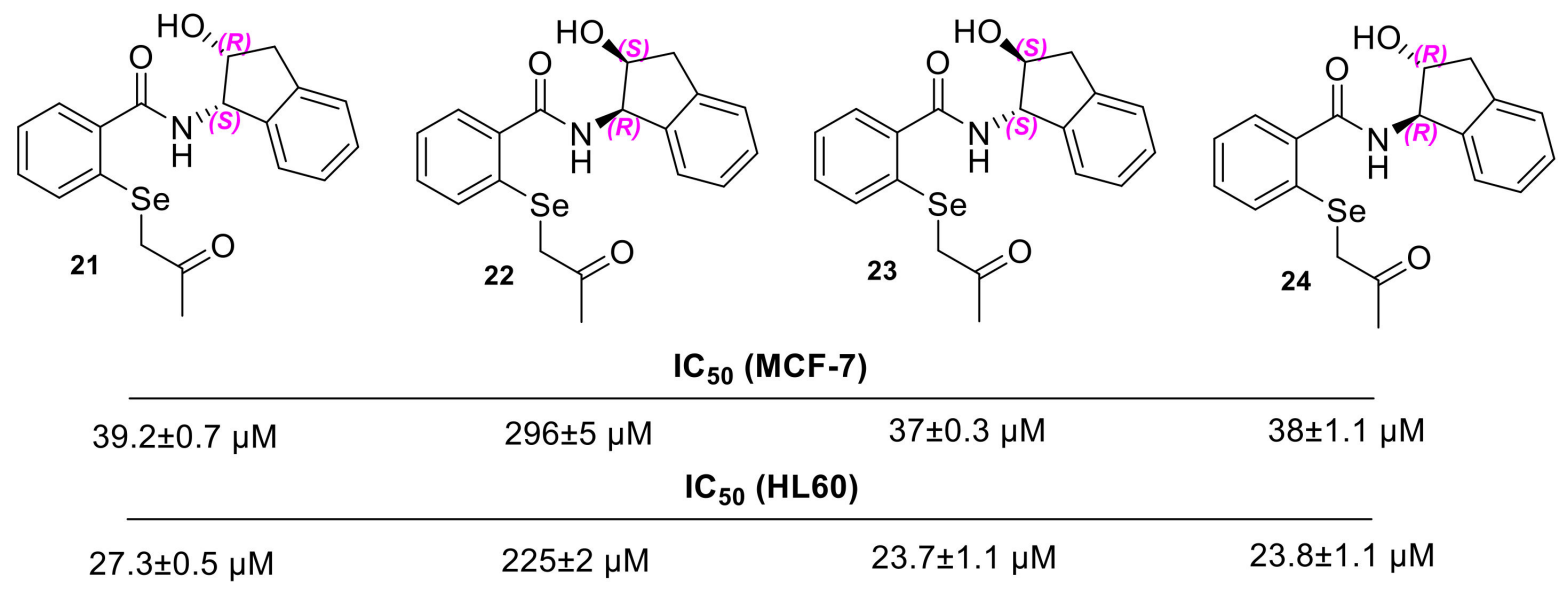
Different bio-activity of diastereomeric hydroxyindanyl selenides **21**–**24**.

In the context of interaction with chiral biological targets, the most interesting phenomenon is to observe a difference in the activity between two enantio- or diastereoisomers. It was observed in the case of *N*-*cis*-2-hydroxy-1-indanyl derivatives **21** and **22**. When the stereochemistry of the C1 and C2 carbon centers was 1*R*,2*S*, the bio-activity towards both cell lines significantly decreased compared to the 1*S*,2*R* isomer. The *trans* epimers **23** and **24** also presented almost 10-fold more reactive than selenide **22** (Figure 3).

## 3. Materials and Methods

### 3.1. General

NMR spectra were recorded on Bruker Avance III/400 or Bruker Avance III/700 (Karlsruhe, Germany) for 1H and 176.1 MHz or 100.6 MHz for 13C (Supplementary Materials). Chemical shifts were recorded relative to SiMe_4_ (δ0.00) or solvent resonance (CDCl_3_ δ7.26, CD3OD δ3.31). Multiplicities were given as s (singlet), d (doublet), dd (double doublet), ddd (double double doublet), t (triplet), dt (double triplet), and m (multiplet). The 77Se NMR spectra were recorded on a Bruker Avance III/400 or Bruker Avance III/700 with diphenyl diselenide as an external standard. NMR spectra were carried out using ACD/NMR Processor Academic Edition. Melting points were measured with a Büchi Tottoli SPM-20 heating unit (Büchi Labortechnik AG, Flawil, Switzerland) and were uncorrected. Elemental analyses were performed on a Vario MACRO CHN analyzer. Optical rotations were measured in 10 mm cells with a polAAr 3000 polarimeter. Column chromatography was performed using Merck 40–63D 60Å silica gel (Merck, Darmstadt, Germany). HPLC analyses were recorded on Shimadzu LC-2060C with phenyl-hexyl column in liquid phase 5–95 or 50–99 (MeOH/H_2_O); for chromatographs, see the Supplementary Materials (Figures S15–S28). All amines were commercially available and purchased from Merck (Merck, Darmstadt, Germany). Their chemical and optical purities were above 97%. The MCF-7 human breast adenocarcinoma cell line was acquired from the European Collection of Cell Cultures (ECACC, Nice, France). Cultivation involved the use of Minimum Essential Medium Eagle (MEME) supplemented with non-essential amino acids, antibiotics (100 mg/mL streptomycin and 100 U/mL penicillin, 2 mM glutamine, all sourced from Sigma-Aldrich, St. Louis, MO, USA), and 10% fetal bovine serum from Biological Industries (Beit-HaEmek, Israel). For the human leukemia cell line HL-60, obtained from the European Collection of Authenticated Cell Cultures (ECACC Nice, France), the culture medium was RPMI 1640 plus GlutaMax I (Invitrogen, Grand Island, NY, USA), supplemented with 10% fetal bovine serum (FBS) and antibiotics (100 μg/mL streptomycin and 100 U/mL penicillin). Cells were maintained at 37 °C in a 5% CO_2_ atmosphere, allowing them to grow until reaching 80% confluence.

### 3.2. General Procedure and Analysis Data

NaHCO_3_ (0.063 g, 0.75 mmol, 1 eq.) was stirred with acetone (2 mL) for 30 min, and then the solution of 2-chloroseleno)benzoyl chloride (0.190 g, 0.75 mmol, 1 eq.) in acetone (1 ml) was added. After 1 h, amine (1 mmol, 1.3 eq.) was added, and the reaction was continued for 3 h (selenides **15**–**18**, **23**, **24**) or 12 h (selenides **11**–**14**, **19**–**22**) at room temperature. After the reaction was finished, the solvent was treated with water (10 mL) and then extracted with DCM. The combined organic layers were dried over magnesium sulfate, and the solvent was evaporated on a rotary evaporator. The crude product was purified by column chromatography (silica gel, DCM) and then recrystallized from a mixture of DCM/heptane.

*N*-((*S*)-(+)-*sec*-butyl)-2-((2-oxopropyl)selanyl)benzamide 11.

Yield: 62%; mp 73–74 °C; ${\left[\propto \right]}_{D}^{20}$ = 33 (c = 0.24, CHCl_3_).

^1^H NMR (400 MHz, DMSO) δ = 0.87 (t, J = 7.6 Hz, 3H), 1.11 (d, J = 6.8 Hz, 3H), 1.44–1.52 (m, 2H), 2.20 (s, 3H), 3.73 (s, 2H), 3.80–3.85 (m, 1H), 7.24 (t, J = 7.6 Hz, 1H_ar_), 7.38 (t, J = 8 Hz, 1H_ar_), 7.50 (d, J = 8 Hz, 1H_ar_), 7.55 (d, J = 7.6 Hz, 1H_ar_), 8.21 (d, J = 8.8 Hz, 1H_ar_) ^13^C NMR (400 MHz, DMSO) δ = 11.15 (CH_3_), 20.64 (CH_3_), 28.67 (CH_3_), 29.26 (CH_2_), 35.66 (CH_2_), 46.97 (CH), 125.80 (CH_ar_), 128.28 (CH_ar_), 129.74 (CH_ar_), 131.00 (CH_ar_), 132.40 (C_ar_), 136.19 (C_ar_), 167.58 (C=O), 205.03 (C=O) ^77^Se NMR (400 MHz, DMSO) δ = 319.53 ppm; IR: 3291, 2960, 2932, 2872, 1694, 1636, 1585, 1532, 1457, 1427, 1356, 1264, 1230, 1141, 1039, 882, 743, 743, 533 cm^−1^. Elemental Anal. Calcd for C_14_H_19_O_2_Se (312.03): C, 53.85; H, 6.13; N, 4.49; Found C, 53.82; H, 6.12; N, 4.48; HPLC purity > 98%.

*N*-((*R*)-(-)-*sec*-butyl)-2-((2-oxopropyl)selanyl)benzamide **12**.

Yield: 56%; mp 72–73 °C; ${\left[\propto \right]}_{D}^{20}$ = −32 (c = 0.31, CHCl_3_).

^1^H NMR (400 MHz, DMSO) δ = 0.87 (t, J = 7.6 Hz, 3H), 1.11 (d, J = 7.2 Hz, 3H), 1.42–1.50 (m, 2H), 2.20 (s, 3H), 3.73 (s, 2H), 3.80–3.89 (m, 1H), 7.24 (t, J = 7.2 Hz, 1H_ar_), 7.36 (t, J = 8 Hz, 1H_ar_), 7.50 (d, J = 8 Hz, 1H_ar_), 7.55 (d, J = 7.6 Hz, 1H_ar_), 8.21 (d, J = 8.4 Hz, 1H_ar_) ^13^C NMR (400 MHz, DMSO) δ = 11.16 (CH_3_), 20.64 (CH_3_), 28.67 (CH_3_), 29.26 (CH_2_), 39.67 (CH_2_), 46.97 (CH), 125.80 (CH_ar_), 128.28 (CH_ar_), 129.75 (CH_ar_), 131.00 (CH_ar_), 132.40 (C_ar_), 136.20 (C_ar_), 167.58 (C=O), 205.03 (C=O) ^77^Se NMR (400 MHz, DMSO) δ = 319.58 ppm; IR: 3299, 2963, 2928, 2873, 1695, 1626, 1583, 1535, 1455, 1431, 1357, 1283, 1230, 1147, 1031, 872, 746, 714, 546 cm^−1^. Elemental Anal. Calcd for C_14_H_19_NO_2_Se (312.03): C, 53.85; H, 6.13; N, 4.49; Found C, 53.86; H, 6.15; N, 4.49; HPLC purity > 97%.

*N*-((*S*)-(+)-1-hydroxy-2-butanyl)-2-((2-oxopropyl)selanyl)benzamide **13**.

Yield: 68%; mp 65–66 °C; ${\left[\propto \right]}_{D}^{20}$ = 32 (c = 0.39, CHCl_3_).

^1^H NMR (400 MHz, DMSO) δ = 0.89 (t, J = 7.2 Hz, 3H), 1.33–1.45 (m, 1H), 1.58–1.67 (m, 1H), 2.20 (s, 3H), 3.40–3.49 (m, 2H), 3.73 (s. 2H), 3.78–3.83 (m, 1H), 4.65 (t, J = 5.6 Hz, 1H), 7.25 (t, J = 7.6 Hz, 1H_ar_), 7.37 (t, J = 8 Hz, 1H_ar_), 7.50 (d, J = 8 Hz, 1H_ar_), 7.61 (d, J = 6.8 Hz, 1H_ar_), 8.08 (d, J = 7.6 Hz, 1H_ar_) ^13^C NMR (400 MHz, DMSO) δ = 11.05 (CH_3_), 24.08 (CH_2_), 28.67 (CH_3_), 39.64 (CH_2_), 53.57 (CH), 63.52 (CH_2_), 125.72 (CH_ar_), 128.45 (CH_ar_), 129.64 (CH_ar_), 131.07 (CH_ar_), 132.61 (C_ar_), 135.95 (C_ar_), 168.11 (C=O), 205.04 (C=O) ^77^Se NMR (400 MHz, DMSO) δ = 321.67 ppm; IR: 3292, 2961, 2874, 1694, 1635, 1585, 1530, 1456, 1355, 1231, 1039, 1017, 880, 743, 685, 577 cm^−1^.Elemental Anal. Calcd for C_14_H_19_NO_3_Se (329.05): C, 51.22; H, 5.83; N, 4.27; Found C, 51.19; H, 5.81; N, 4.27; HPLC purity > 97%.

*N*-((*R*)-(-)-1-hydroxy-2-butanyl)-2-((2-oxopropyl)selanyl)benzamide **14**.

Yield: 95%; mp 66–67 °C; ${\left[\propto \right]}_{D}^{20}$ = −35 (c = 0.49, CHCl_3_).

^1^H NMR (400 MHz, DMSO) δ = 0.90 (t, J = 7.6 Hz, 3H), 1.36–1.46 (m, 1H), 1.61–1.71 (m, 1H), 2.23 (s, 3H), 3.45–3.50 (m, 2H), 3.76 (s. 2H), 3.78–3.85 (m, 1H), 4.68 (t, J = 5.6 Hz, 1H), 7.25 (t, J = 7.6 Hz, 1H_ar_), 7.38 (t, J = 8 Hz, 1H_ar_), 7.52 (d, J = 8 Hz, 1H_ar_), 7.63 (d, J = 8 Hz, 1H_ar_), 8.10 (d, J = 8.4 Hz, 1H_ar_) ^13^C NMR (400 MHz, DMSO) δ = 11.05 (CH_3_), 24.08 (CH_2_), 28.67 (CH_3_), 39.64 (CH_2_), 53.57 (CH), 63.52 (CH_2_), 125.72 (CH_ar_), 128.45 (CH_ar_), 129.64 (CH_ar_), 131.06 (CH_ar_), 132.61 (C_ar_), 135.96 (C_ar_), 168.11 (C = O), 205.04 (C = O) ^77^Se NMR (400 MHz, DMSO) δ = 321.67 ppm; IR: 3291, 2961, 2872, 1694, 1636, 1585, 1532, 1457, 1355, 1230, 1039, 1018, 881, 743, 685, 576 cm^−1^. Elemental Anal. Calcd for C, 51.22; H, 5.83; N, 4.27; Found C, 51.23; H, 5.84; N, 4.29; HPLC purity > 97%.

*N*-((*R*)-(-)-1,2,3,4-tetrahydro-1-naphthyl) 2-((2-oxopropyl)selanyl)benzamide **15**.

Yield: 62%; mp 115–116 °C; ${\left[\propto \right]}_{D}^{20}$ = −35 (c = 0.76, CHCl_3_).

^1^H NMR (400 MHz, DMSO) δ = 1.73–1.81 (m, 2H), 1.92–2.00 (m, 2H), 2.23 (s, 3H), 2.73–2.78 (m, 2H), 3.77 (s, 2H), 5.52–5.59 (m, 1H), 7.11–7.19 (m, 3H_ar_), 7.21–7.28 (m, 2H_ar_), 7.37 (t, J = 8 Hz, 1H_ar_), 7.53 (d, J = 8 Hz, 1H_ar_), 7.60 (d, J = 8 Hz, 1H_ar_), 8.85 (d, J = 8.8 Hz, 1H) ^13^C NMR (700 MHz, DMSO) δ = 20.83 (CH_2_), 28.70 (CH_3_), 29.32 (CH_2_), 30.28 (CH_2_), 35.77 (CH_2_), 47.64 (CH), 125.84 (CH_ar_), 126.29 (CH_ar_), 127.16 (CH_ar_), 128.32 (CH_ar_), 128.48 (CH_ar_), 129.22 (CH_ar_), 129.84 (CH_ar_), 131.17 (CH_ar_), 132.62 (C_ar_), 135.81 (C_ar_), 137.65 (C_ar_), 137.84 (C_ar_), 167.84 (C=O), 205.03 (C=O) ^77^Se NMR (700 MHz, DMSO) δ = 321.23 ppm; IR: 3354, 2999, 2941, 1738, 1680, 1627, 1584, 1530, 1488, 1430, 1356, 1261, 1077, 738, 561 cm^−1^. Elemental Anal. Calcd for C_20_H_21_NO_2_Se (387.07): C, 62.18; H, 5.48; N, 3.63; Found C, 62.15; H, 5.51; N, 3.65; HPLC purity > 98%.

*N*-((*S*)-(+)-1,2,3,4-tetrahydro-1-naphthyl)-2-((2-oxopropyl)selanyl)benzamide **16**.

Yield: 59%; mp 114–115 °C; ${\left[\propto \right]}_{D}^{20}$ = 34 (c = 0.74, CHCl_3_).

^1^H NMR (400 MHz, DMSO) δ = 1.74–1.81 (m, 2H), 1.90–1.99 (m, 2H), 2.22 (s, 3H), 2.63–2.68 (m, 2H), 3.77 (s, 2H), 5.51–5.60 (m, 1H), 7.14–7.18 (m, 3H_ar_), 7.20–7.28 (m, 2H_ar_), 7.37 (t, J = 7.2 Hz, 1H_ar_), 7.53 (d, J = 7.2 Hz, 1H_ar_), 7.59 (d, J = 7.6 Hz, 1H_ar_), 8.83 (d, J = 8.8 Hz, 1H) ^13^C NMR (400 MHz, DMSO) δ = 20.82 (CH_2_), 28.69 (CH_3_), 29.30 (CH_2_), 30.27 (CH_2_), 35.78 (CH_2_), 47.64 (CH), 125.85 (CH_ar_), 126.28 (CH_ar_), 127.16 (CH_ar_), 128.31 (CH_ar_), 128.46 (CH_ar_), 129.22 (CH_ar_), 129.87 (CH_ar_), 131.16 (CH_ar_), 132.56 (C_ar_), 135.84 (C_ar_), 137.65 (C_ar_), 137.82 (C_ar_), 167.85 (C=O), 205.03 (C=O) ^77^Se NMR (400 MHz, DMSO) δ = 319.81 ppm; IR: 3353, 2999, 2941, 1738, 1681, 1627, 1585, 1532, 1488, 1432, 1357, 1262, 1078, 739, 562 cm^−1^. Elemental Anal. Calcd for C_20_H_21_NO_2_Se (387.07): C, 62.18; H, 5.48; N, 3.63; Found C, 62.19; H, 5.47; N, 3.63; HPLC purity > 98%.

*N*-((*R*)-(+)-α-methylbenzyl)-2-((2-oxopropyl)selanyl)benzamide **17**.

Yield: 87%; mp 133–134°C; ${\left[\propto \right]}_{D}^{20}$ = 64 (c = 0.78, CHCl_3_).

^1^H NMR 400 MHz, DMSO) δ = 1.44 (d, J = 7.2 Hz, 3H), 2.19 (s, 3H), 3.72 (s, 2H), 5.07–5.13 (m, 1H), 7.17–7.24 (m, 1H_ar_), 7.24–7.34 (m, 3H_ar_), 7.35–7.42 (m, 3H_ar_), 7.51 (d, J = 8 Hz, 1H_ar_), 7.68 (d, J = 6 Hz, 1H_ar_), 8.88 (d, J = 8 Hz, 1H) ^13^C NMR (300 MHz, DMSO) δ = 22.74 (CH_3_), 28.67 (CH_3_), 35.65 (CH_2_), 48.97 (CH), 125.78 (CH_ar_), 126.49 (2xCH_ar_), 127.10 (CH_ar_), 128.59 (CH_ar_), 128.69 (2xCH_ar_), 129.73 (CH_ar_), 131.25 (CH_ar_), 132.84 (C_ar_), 135.38 (C_ar_), 145.07 (C_ar_), 167.31 (C=O), 204.99 (C=O) ^77^Se NMR (400 MHz, DMSO) δ = 322.26 ppm; IR: 3337, 2989, 2970, 1738, 1678, 1631, 1581, 1530, 1454, 1415, 1353, 1330, 1230, 1156, 755, 746, 686, 554, 541 cm^−1^. Elemental Anal. Calcd for C_18_H_19_NO_2_Se (361.06): C, 60.00; H, 5.32; N, 3.89; Found C, 59.95; H, 5.31; N, 3.89; HPLC purity > 98%.

*N*-((*S*)-(-)-α-methylbenzyl)-2-((2-oxopropyl)selanyl)benzamide **18**.

Yield: 80%; mp 132–133°C; ${\left[\propto \right]}_{D}^{20}$ = −61 (c = 0.60, CHCl_3_).

^1^H NMR 400 MHz, DMSO) δ = 1.46 (d, J = 7.2 Hz, 3H), 2.21 (s, 3H), 3.75 (s, 2H), 5.07–5.13 (m, 1H), 7.17–7.24 (m, 1H_ar_), 7.24–7.34 (m, 3H_ar_), 7.35–7.42 (m, 3H_ar_), 7.53 (d, J = 8 Hz, 1H_ar_), 7.69 (d, J = 6 Hz, 1H_ar_), 8.91 (d, J = 8 Hz, 1H) ^13^C NMR (300 MHz, DMSO) δ = 22.72 (CH_3_), 28.67 (CH_3_), 35.67 (CH_2_), 48.99 (CH), 125.81 (CH_ar_), 126.50 (2xCH_ar_), 127.10 (CH_ar_), 128.59 (CH_ar_), 128.70 (2xCH_ar_), 129.73 (CH_ar_), 131.25 (CH_ar_), 132.78 (C_ar_), 135.47 (C_ar_), 145.06 (C_ar_), 167.34 (C=O), 204.98 (C=O) ^77^Se NMR (400 MHz, DMSO) δ = 322.03 ppm; IR: 3336, 2989, 2970, 1738, 1678, 1630, 1582, 1529, 1455, 1414, 1354, 1331, 1232, 1157, 755, 747, 686, 555, 541 cm^−1^. Elemental Anal. Calcd for C_18_H_19_NO_2_Se (361.06): C, 60.00; H, 5.3; N, 3.89; Found C, 59.99; H, 5.30; N, 3.87; HPLC purity > 99%.

*N*-((*S*)-(-)-1-(1-naphthyl)ethyl)-2-((2-oxopropyl)selanyl)benzamide **19**.

Yield: 50%; mp 161–162°C; ${\left[\propto \right]}_{D}^{20}$ = −36 (c = 0.55, CHCl_3_).

^1^H NMR (400 MHz, DMSO) δ = 1.61 (d, J = 6.8 Hz, 3H), 2.21 (s, 3H), 3.75 (s, 2H), 5.90 (quin, J = 5.6 Hz, 1H), 7.29 (t, J = 7.2 Hz, 1H_ar_), 7.40 (t, J = 7.6 Hz, 1H_ar_), 7.44–7.57 (m, 5H_ar_), 7.69 (d, J = 7.6 Hz, 1H_ar_), 7.85 (d, J = 8 Hz, 1H_ar_), 7.96 (d, J = 7.6 Hz, 1H_ar_), 8.23 (d, J = 8 Hz, 1H_ar_), 9.09 (d, J = 7.6 Hz, 1H) ^13^C NMR (400 MHz, DMSO) δ = 21.89 (CH_3_), 28.65 (CH_3_), 39.64 (CH_2_), 45.31 (CH), 123.05 (CH_ar_), 123.68 (CH_ar_), 125.79 (CH_ar_), 125.93 (CH_ar_), 126.06 (CH_ar_), 126.67 (CH_ar_), 127.77 (CH_ar_), 128.64 (CH_ar_), 129.13 (CH_ar_), 129.77 (CH_ar_), 130.88 (C_ar_), 131.25 (CH_ar_), 132.76 (C_ar_), 133.85 (C_ar_), 135.47 (C_ar_), 140.52 (C_ar_), 167.26 (C=O), 204.93 (C=O) ^77^Se NMR (400 MHz, DMSO) δ = 322.03 ppm; IR: 3378, 2971, 1738, 1693, 1633, 1537, 1455, 1356, 1257, 1133, 1031, 801, 781, 737, 643, 542 cm^−1^. Elemental Anal. Calcd for C_22_H_21_NO_2_Se (411.07): C, 64.39; H, 5.16; N, 3.41; Found C, 64.46; H, 5.15; N, 3.42; HPLC purity >98%.

*N*-((*R*)-(+)-1-(1-naphthyl)ethyl)-2-((2-oxopropyl)selanyl)benzamide **20**.

Yield: 41%; mp 161–162°C; ${\left[\propto \right]}_{D}^{20}$ = 37 (c = 0.49, CHCl_3_).

^1^H NMR (400 MHz, DMSO) δ = 1.58 (d, J = 7.2 Hz, 3H), 2.18 (s, 3H), 3.73 (s, 2H), 5.87 (quin, J = 7.6 Hz, 1H), 7.25 (t, J = 7.2 Hz, 1H_ar_), 7.37 (t, J = 7.2 Hz, 1H_ar_), 7.45–7.64 (m, 5H_ar_), 7.67 (d, J = 7.6 Hz, 1H_ar_), 7.82 (d, J = 8 Hz, 1H_ar_), 7.93 (d, J = 8 Hz, 1H_ar_), 8.21 (d, J = 8 Hz, 1H_ar_), 9.07 (d, J = 7.6 Hz, 1H) ^13^C NMR (400 MHz, DMSO) δ = 21.89 (CH_3_), 28.65 (CH_3_), 39.61 (CH_2_), 45.30 (CH), 123.05 (CH_ar_), 123.68 (CH_ar_), 125.78 (CH_ar_), 125.93 (CH_ar_), 126.06 (CH_ar_), 126.66 (CH_ar_), 127.77 (CH_ar_), 128.63 (CH_ar_), 129.12 (CH_ar_), 129.77 (CH_ar_), 130.88 (C_ar_), 131.25 (CH_ar_), 132.76 (C_ar_), 133.85 (C_ar_), 135.47 (C_ar_), 140.51 (C_ar_), 167.25 (C=O), 204.92 (C=O) ^77^Se NMR (400 MHz, DMSO) δ = 322.07 ppm; IR: 3377, 2970, 1738, 1693, 1633, 1537, 1454, 1365, 1257, 1132, 1032, 801, 781, 737, 642, 542 cm^−1^. Elemental Anal. Calcd for C_22_H_21_NO_2_Se (411.07): C, 64.39; H, 5.16; N, 3.41; Found C, 64.44; H, 5.18; N, 3.43;. HPLC purity > 98%.

*N*-((1*S*,2*R*)-(-)-*cis*-2-hydroxy-1-indanyl)-2-((2-oxopropyl)selanyl)benzamide **21**.

Yield: 76%; mp 111–112 °C; ${\left[\propto \right]}_{D}^{20}$ = −45 (c = 0.38, CHCl_3_).

^1^H NMR (700 MHz, DMSO) δ = 2.26 (s, 3H), 2.86–2.90 (m, 1H), 3.08–3.13 (m, 1H), 3.80 (d, J = 5.6 Hz, 2H), 4.50–4.53 (m, 1H), 5.12 (d, J = 4.2 Hz, 1H), 5.38–5.42 (m, 1H), 7.19–7.25 (m, 2H_ar_), 7.26–7.29 (m, 3H_ar_), 7.41 (t, J = 7.7 Hz, 1H_ar_), 7.56 (d, J = 7.7 Hz, 1H_ar_), 7.79 (d, J = 7.7 Hz, 1H_ar_), 8.32 (d, J = 9.1 Hz, 1H) ^13^C NMR (700 MHz, DMSO) δ = 28.78 (CH_3_), 35.75 (CH_2_), 40.18 (CH_2_), 57.85 (CH), 72.55 (CH), 124.81 (CH_ar_), 125.31 (CH_ar_), 125.76 (CH_ar_), 126.77 (CH_ar_), 127.88 (CH_ar_), 128.98 (CH_ar_), 129.60 (CH_ar_), 131.37 (CH_ar_), 133.13 (C_ar_), 135.09 (C_ar_), 141.36 (C_ar_), 142.20 (C_ar_), 168.33 (C=O), 205.10 (C=O) ^77^Se NMR (700 MHz, DMSO) δ = 324.41 ppm; IR: : 3283, 2947, 2926, 1724, 1634, 1583, 1562, 1477, 1423, 1353, 1227, 1080, 793, 737, 584 cm^−1^. Elemental Anal. Calcd for C_19_H_19_NO_3_Se (389.05): C, 58.77; H, 4.93; N, 3.61; Found C, 58.81; H, 4.90; N, 3.59; HPLC purity > 99%.

*N*-((1*R*,2*S*)-(+)-*cis*-2-hydroxy-1-indanyl)-2-((2-oxopropyl)selanyl)benzamide **22**.

Yield: 65%; mp 110–111 °C; ${\left[\propto \right]}_{D}^{20}$ = 48 (c = 0.42, CHCl_3_).

^1^H NMR (700 MHz, DMSO) δ = 2.26 (s, 3H), 2.87–2.91 (m, 1H), 3.08–3.14 (m, 1H), 3.80 (d, J = 5.6 Hz, 2H), 4.51–4.55 (m, 1H), 5.12 (d, J = 4.2 Hz, 1H), 5.39–5.42 (m, 1H), 7.18–7.25 (m, 2H_ar_), 7.26–7.29 (m, 3H_ar_), 7.41 (t, J = 7.7 Hz, 1H_ar_), 7.56 (d, J = 7 Hz, 1H_ar_), 7.80 (d, J = 7.7 Hz, 1H_ar_), 8.32 (d, J = 8.4 Hz, 1H) ^13^C NMR (400 MHz, DMSO) δ = 28.78 (CH_3_), 35.75 (CH_2_), 40.18 (CH_2_), 57.85 (CH), 72.55 (CH), 124.81 (CH_ar_), 125.32 (CH_ar_), 125.77 (CH_ar_), 126.78 (CH_ar_), 127.88 (CH_ar_), 128.97 (CH_ar_), 129.61 (CH_ar_), 131.37 (CH_ar_), 133.10 (C_ar_), 135.12 (C_ar_), 141.36 (C_ar_), 142.19 (C_ar_), 168.34 (C=O), 205.10 (C=O) ^77^Se NMR (700 MHz, DMSO) δ = 325.43 ppm; IR: 3349, 2923, 2853, 1724, 1626, 1584, 1560, 1509, 1459, 1354, 1228, 1094, 795, 738, 580 cm^−1^. Elemental Anal. Calcd for C_19_H_19_NO_3_Se (389.05): C, 58.77; H, 4.93; N, 3.61; Found C, 58.76; H, 4.94; N, 3.60; HPLC purity > 98%.

*N*-((1*S*,2*S*)-(-)-*trans*-2-hydroxy-1-indanyl) 2-((2-oxopropyl)selanyl)benzamide **23**.

Yield: 17%; mp 112–114 °C; ${\left[\propto \right]}_{D}^{20}$ = 35 (c = 0.38, CHCl_3_).

^1^H NMR (700 MHz, DMSO) δ = 2.26 (s, 3H), 2.71–2.79 (m, 1H), 3.13–3.19 (m, 1H), 3.80 (d, J = 7 Hz, 2H), 4.42 (quin, J = 7 Hz, 1H), 5.22 (t, J = 7.7 Hz, 1H), 5.39 (d, J = 5.6 Hz, 1H), 7.26–7.34 (m, 4H_ar_), 7.28 (t, J = 6.3 Hz, 1H_ar_), 7.41 (t, J = 7 Hz, 1H_ar_), 7.57 (d, J = 5.6 Hz, 1H_ar_), 7.70 (d, J=7.7 Hz, 1H_ar_), 8.84 (d, J = 5.6 Hz, 1H) ^13^C NMR (300 MHz, DMSO) δ = 28.71 (CH_3_), 35.67 (CH_2_), 39.11 (CH_2_), 61.97 (CH), 77.74 (CH), 124.34 (CH_ar_), 125.11 (CH_ar_), 125.76 (CH_ar_), 127.11 (CH_ar_), 128.09 (CH_ar_), 128.62 (CH_ar_), 129.66 (CH_ar_), 131.31 (CH_ar_), 132.95 (C_ar_), 135.38 (C_ar_), 140.26 (C_ar_), 142.26 (C_ar_), 168.55 (C=O), 205.08 (C=O) ^77^Se NMR (700 MHz, DMSO) δ = 321.18 ppm; IR: 3303, 2997, 2940, 1738, 1677, 1642, 1541, 1364, 1232, 748, 647, 542, 517 cm^−1^. Elemental Anal. Calcd for C_19_H_19_NO_3_Se (389.05): C, 58.77; H, 4.93; N, 3.61; Found C, 58.67; H, 4.91; N, 3.62; HPLC purity > 98%.

*N*-((1*R*,2*R*)-(-)-*trans*-2-hydroxy-1-indanyl)-2-((2-oxopropyl)selanyl)benzamide **24**.

Yield: 68%; mp 112–114 °C; ${\left[\propto \right]}_{D}^{20}$ = −31 (c = 0.58, CHCl_3_).

^1^H NMR (700 MHz, DMSO) δ = 2.26 (s, 3H), 2.74–2.79 (m, 1H), 3.13–3.19 (m, 1H), 3.80 (d, J = 7 Hz, 2H), 4.42 (quin, J = 7 Hz, 1H), 5.22 (t, J = 7.7 Hz, 1H), 5.39 (d, J = 5.6 Hz, 1H), 7.17–7.24 (m, 4H_ar_), 7.29 (t, J = 7 Hz, 1H_ar_), 7.41 (t, J = 6.3 Hz, 1H_ar_), 7.57 (d, J = 6.3 Hz, 1H_ar_), 7.70 (d, J = 6.3 Hz, 1H_ar_), 8.84 (d, J = 8.4 Hz, 1H) ^13^C NMR (300 MHz, DMSO) δ = 28.71 (CH_3_), 35.68 (CH_2_), 39.11 (CH_2_), 61.97 (CH), 77.74 (CH), 124.32 (CH_ar_), 125.11 (CH_ar_), 125.75 (CH_ar_), 127.12 (CH_ar_), 128.10 (CH_ar_), 128.63 (CH_ar_), 129.68 (CH_ar_), 131.31 (CH_ar_), 132.97 (C_ar_), 135.36 (C_ar_), 140.27 (C_ar_), 142.27 (C_ar_), 168.54 (C=O), 205.08 (C=O) ^77^Se NMR (700 MHz, DMSO) δ = 324.38 ppm; IR: 3305, 2997, 2942, 1738, 1677, 1642, 1541, 1365, 1229, 748, 647, 528, 517 cm^−1^. Elemental Anal. Calcd for C_19_H_19_NO_3_Se (389.05): C, 58.77; H, 4.93; N, 3.61; Found C, 58.71; H, 4.95; N, 3.63; HPLC purity > 97%.

### 3.3. Antioxidant Activity Evaluation

#### 3.3.1. DTT Activity Assay

To a solution of compounds **11**–**24** (0.015 mmol) and dithiothreitol DTT^red^ (0.15 mmol) in 1.1 mL of CD_3_OD, 30% H_2_O_2_ (0.15 mmol) was added. ^1^H NMR spectra were measured right after the addition of hydrogen peroxide and then in specific time intervals. The concentration of the substrate was determined according to the changes in the integration on the ^1^H NMR spectra [25], Supplementary Materials (Figures S29–S31).

#### 3.3.2. DPPH Radical Scavenging Assay

The 2,2-di(4-tert-octylphenyl)-1-picrylhydrazyl) test is based on the capacity of tested compounds to scavenge the stable free radical DPPH, and the obtained results are expressed as inhibitory concentration (IC50).

The radical neutralization test was conducted according to the previously described method [39]. In brief, calibration curves were created for each tested compound by increasing volumes of selenides. Solutions in methanol to a 0.5 mL methanolic DPPH radical (0.3 mM) made up with methanol to the 2.0 mL. All solutions were measured in triplicate against a reagent blank (2 mL of methanol + 0.5 mL of DPPH methanolic solution) after 30 min at 517 nm using a UV-1601 spectrophotometer (Shimadzu, Kyoto, Japan).

The inhibition ratio (%) was obtained from the following equation:

$$\mathrm{inhibition}\;\mathrm{ratio}\;(\%)=\frac{A1-A0}{A0}\cdot 100\%,\;\mathrm{where}:$$

A1—absorbance of sample

A0—absorbance of the reagent blank.

Next, the 50% DPPH inhibition (IC_50_) was calculated by the linear regression analysis between the radical scavenging percentage against the tested compound concentration. All obtained curves are presented in Supplementary Materials (Figures S32–S39).

Additionally, the antioxidant capacity (AC) of tested compounds was determined and expressed as millimoles of Trolox (TE) equivalent per 1 g of compounds. For this purpose, the calibration curve of inhibition ratio (%DPPH) vs. TE concentration was prepared and presented in the Supporting Information (Figure S22). Finally, the results of DPPH radical scavenging activity by *β*-carbonyl selenides were presented as Trolox equivalent antioxidant capacity (TEAC) and calculated as follows [31,32]:

$$\mathrm{Trolox}\;\mathrm{equivalent}\;\mathrm{antioxidant}\;\mathrm{capacity}=\frac{TroloxIC50[mM]}{Tested\;\beta -carbonylselenidesIC50[mM]}$$

### 3.4. MTT Viability Assay

The MTT (3-(4,5-didiazol-2-yl)-2,5 diphenyl tetrazolium bromide) assay, which measures the activity of methylcellular dehydrogenases, was based on Mosmann’s method [35]. Briefly, cells were seeded into 96-well plates (about 1.5 × 10^4^ cells per well, in 100 µL) and then left to adhere and grow for 24 h. Subsequently, 100 µL of the tested compounds in the medium was added to a final concentration of 0–250 μM for 24 h, followed by the addition of 100 µL MTT, 3 mg/mL in PBS, for the next 3 h. After the incubation, the medium was removed. The remaining insoluble formazan crystals were dissolved in 100 µL DMSO. The absorbance of the blue formazan product was measured at 570 nm in the plate reader spectrophotometer Infinite M200 (Tecan, Austria) and compared with the control (untreated cells). All experiments were performed three times in triplicate. The concentration of the tested compounds required to inhibit cell viability by 50% (IC_50_) was calculated using Microsoft Excel software for semi-log curve fitting with linear regression analysis.

## 4. Conclusions

Herein, we optimized the synthesis of chiral β-carbonyl selenides and proved that the generation of the carbanion from the starting ketone is crucial for the high efficiency of the reaction. This procedure was further used for the synthesis of *N*-substituted derivatives in the form of enantiomeric and diastereomeric pairs, presenting the first examples of unsymmetrical (2-oxopropyl)selanyl) benzamides possessing chiral groups attached to the nitrogen atom. All compounds were tested as antioxidants by two assays: the DTT method, presenting the ability to reduce H_2_O_2_, and the DPPH procedure for evaluating radical scavenging capacity. In both cases, derivatives possessing hydroxy groups were the most active ones—selenides with 2-hydroxy group in *cis* (DTT assay) and *trans* configuration (DTT and DPPH method) and 1-hydroxy-2-butanyl substituent (DPPH assay). Additionally, the antiproliferative capacity towards breast cancer MCF-7 and human promyelocytic leukemia HL-60 cell lines was measured. Also, in this case, both enantiomers of *N*-(1-hydroxy-2-butanyl) selenides were the most potent cytotoxic agents with similar IC50 values. Considering the chirality–activity dependence, we have observed that the configurations of C1 and C2 carbon of the *N*-2-hydroxy-1-indanyl derivatives influence their antiproliferative potential. The results indicate that the N-1-hydroxy-2-butanyl and N-2-hydroxy-1-indanyl substituents can be selected as possible pharmacophores to develop potential Se therapeutics with optimal drug–target interaction further.

## Figures and Tables

**Table 1:** Optimization of reaction conditions.

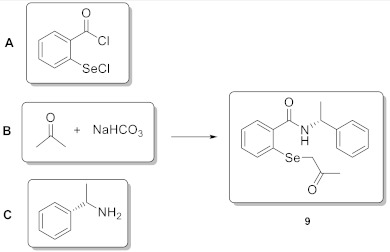				
Entry	Solvent (Solubilization of A)	NaHCO_3_	Conditions and Order of Addition	Yield (%)
1	DCM	-	1. **A** + acetone, rt, 1 h 2. **C**, rt, 20 min	30
2	DCM	1 eq.	1. **A** + **B**, rt, 1 h 2. **C**, rt, 3 h	41
3	-	1 eq. (0.75 M NaHCO_3_/H_2_O)	1. **A** + **C**, rt, 20 min 2. **B**, 0 °C to rt, 2 h	37
4	Acetone	1 eq. (0.75 M NaHCO_3_/H_2_O)	1. **B** (acetone + H_2_O solution of NaHCO_3_, rt, 25 min) + **A**, 0 °C to rt, 1 h 2. **C**, rt, 2 h	0
5	THF	1 eq.	1. **B** (acetone + NaHCO_3_, rt, 30 min) + **A**, rt, 1 h 2. **C**, rt, 3 h	65
6	Acetone	1 eq.	1. **B** (acetone + NaHCO_3_, rt, 30 min) + **A**, rt, 1 h 2. **C**, rt, 3 h	87

**Table 2:** Results of the antioxidant activity measurement.

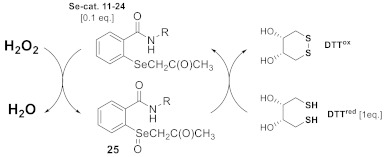				
	Remaining DTT^red^ (%)			
Catalyst (0.1 equiv.)	5 min	15 min	30 min	60 min
**11**/**12**	79 ± 5.4	61 ± 4.8	44 ± 2.8	13 ± 3.7
**13**/**14**	72 ± 7.4	61 ± 6.1	48 ± 3.7	21 ± 1.7
**15**/**16**	86 ± 0.1	78 ± 0.2	69 ± 0.1	56 ± 0.5
**17**/**18**	91 ± 2.2	90 ± 0.7	82 ± 1.4	73 ± 0.5
**19**/**20**	93 ± 0.1	90 ± 0.6	83 ± 0.8	70 ± 0.2
**21**/**22**	72 ± 2.5	52 ± 0.2	27 ± 2.7	6 ± 4.3
**23**/**24**	79 ± 0.3	56 ± 5.3	30 ± 3.3	10 ± 2.4
**Ebselen**	75	64	58	52

**Table 3:** Results of DPPH test.

Compound	IC_50_ (mM)	Antioxidant Capacity (mM TE·1 g^−1^)
**11**/**12**	5.61	0.10
**13**/**14**	0.89	0.47
**15**/**16**	2.28	0.16
**17**/**18**	2.59	0.12
**19**/**20**	1.62	0.23
**21**/**22**	2.51	0.19
**23**/**24**	0.96	0.39

**Table 4:** The antiproliferative activity of compounds **11**–**24**.

Compound	IC_50_ [µM] ± SEM	
	MCF-7	HL-60
**11**	46.1 ± 1.6	181 ± 11
**12**	55 ± 1.4	101 ± 14
**13**	39.2 ± 0.9	14.4 ± 0.5
**14**	35.7 ± 0.6	16.2 ± 1.1
**15**	115 ± 21	111 ± 3.5
**16**	188.3 ± 0.3	142 ± 1.7
**17**	235 ± 1	303 ± 3
**18**	237 ± 11	23.5 ± 1.4
**19**	87.4 ± 0.5	101 ± 0.6
**20**	72.4 ± 0.8	84.5 ± 0.6
**21**	39.2 ± 0.7	27.3 ± 0.5
**22**	296 ± 5	225 ± 2
**23**	37 ± 0.3	23.7 ± 1.1
**24** **Oxaliplatin**	38 ± 1.1 35 [36,37]	23.8 ± 1.1 0.8 [38]

**Table 5:** The antiproliferative activity of *N*-(1-hydroxy-2-butanyl) Se derivatives.

	IC_50_ [µM] ± SEM			
	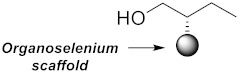		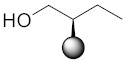	
Se-derivative	MCF-7	HL-60	MCF-7	HL-60
*β*-Carbonylselenide	39.2 ± 0.9	14.4 ± 0.5	35.7 ± 0.6	16.2 ± 1.1
Phenylselenide	>150	>150	>150	>150
Benzisoselenazolone	38.3 ± 1.3	26,0. ± 1.7	35.1 ± 0.5	33.3 ± 0.5
Diselenide	37.00 ± 4.25	8.67 ± 0.14	>100	10.10 ± 0.49

## Data Availability

The original contributions presented in the study are included in the article/Supplementary Material, further inquiries can be directed to the corresponding author.

## Supplementary Materials

The following supporting information can be downloaded at: https://www.mdpi.com/article/10.3390/ma17040899/s1, Figure S1: (a) ^1^H NMR, (b) ^13^C NMR, and (c) ^77^Se NMR spectra of *N*-((*S*)-(+)-*sec*-butyl)- 2-((2-oxopropyl)selanyl)benzamide 11; Figure S2: (a) ^1^H NMR, (b) ^13^C NMR, and (c) ^77^Se NMR spectra of *N*-((*R*)-(-)-*sec*-butyl)- 2-((2-oxopropyl)selanyl)benzamide 12; Figure S3: (a) ^1^H NMR, (b) ^13^C NMR, and (c) ^77^Se NMR spectra of *N*-((*S*)-(+)-1-hydroksy-2-butanyl)-2-((2-oxopropyl)selanyl)benzamide 13; Figure S4: (a) ^1^H NMR, (b) ^13^C NMR, and (c) ^77^Se NMR spectra of *N*-((*R*)-(-)-1-hydroksy-2-butanyl)-2-((2-oxopropyl)selanyl)benzamide 14; Figure S5: (a) ^1^H NMR, (b) ^13^C NMR, and (c) ^77^Se NMR spectra of *N*-((*R*)-(-)-1,2,3,4-tetrahydro-1-napthyl)-2-((2-oxopropyl)selanyl)benzamide 15; Figure S6: (a) ^1^H NMR, (b) ^13^C NMR, and (c) ^77^Se NMR spectra of *N*-((*S*)-(+)-1,2,3,4-tetrahydro-1-napthyl)-2-((2-oxopropyl)selanyl)benzamide 16; Figure S7: (a) ^1^H NMR, (b) ^13^C NMR, and (c) ^77^Se NMR spectra of *N*-((*R*)-(+)-α-metylbenzyl)-2-((2-oxopropyl)selanyl)benzamide 17; Figure S8: (a) ^1^H NMR, (b) ^13^C NMR, and (c) ^77^Se NMR spectra of *N*-((*S*)-(-)-α-metylbenzyl)-2-((2-oxopropyl)selanyl)benzamide 18; Figure S9: (a) ^1^H NMR, (b) ^13^C NMR, and (c) ^77^Se NMR spectra of *N*-((*S*)-(-)-1-(1-napthyl)etyl)-2-((2-oxopropyl)selanyl)benzamide 19; Figure S10: (a) ^1^H NMR, (b) ^13^C NMR, and (c) 77Se NMR spectra of *N*-((*R*)-(+)-1-(1-napthyl)etyl)-2-((2-oxopropyl)selanyl)benzamide 20; Figure S11: (a) ^1^H NMR, (b) ^13^C NMR, and (c) ^77^Se NMR spectra of *N*-((1*S*,2*R*)-(-)-cis-2-hydroksy-1-indanyl)-2-((2-oxopropyl)selanyl)benzamide 21; Figure S12: (a) ^1^H NMR, (b) ^13^C NMR, and (c) ^77^Se NMR spectra of N-((1*R*,2*S*)-(+)-cis-2-hydroksy-1-indanyl)-2-((2-oxopropyl)selanyl)benzamide 22; Figure S13: (a) ^1^H NMR, (b) ^13^C NMR, and (c) ^77^Se NMR spectra of *N*-((1*S*,2*S*)-(+)-trans-2-hydroksy-1-indanyl)-2-((2-oxopropyl)selanyl)benzamide 23; Figure S14: (a) ^1^H NMR, (b) ^13^C NMR, and (c) ^77^Se NMR spectra of *N*-((1R,2R)-(-)-trans-2-hydroksy-1-indanyl)-2-((2-oxopropyl)selanyl)benzamide 24; Figure S15: Chromatograph of *N*-((S)-(+)-sec-butyl)-2-((2-oxopropyl)selanyl)benzamide 11; Figure S16: Chromatograph of *N*-((R)-(-)-sec-butyl)-2-((2-oxopropyl)selanyl)benzamide 12; Figure S17: Chromatograph of *N*-((*S*)-(+)-1-hydroksy-2-butanyl)-2-((2-oxopropyl)selanyl)benzamide 13; Figure S18: Chromatograph of *N*-((R)-(-)-1-hydroksy-2-butanyl)-2-((2-oxopropyl)selanyl)benzamide 14; Figure S19: Chromatograph of *N*-((*R*)-(-)-1,2,3,4-tetrahydro-1-napthyl)-2-((2-oxopropyl)selanyl)benzamide 15; Figure S20: Chromatograph of *N*-((*S*)-(+)-1,2,3,4-tetrahydro-1-napthyl)-2-((2-oxopropyl)selanyl)benzamide 16; Figure S21: Chromatograph of *N*-((*R*)-(+)-α-metylbenzyl)-2-((2-oxopropyl)selanyl)benzamide 17; Figure S22: Chromatograph of *N*-((*S*)-(-)-α-metylbenzyl)-2-((2-oxopropyl)selanyl)benzamide 18; Figure S23: Chromatograph of *N*-((*S*)-(-)-1-(1-napthyl)etyl)- 2-((2-oxopropyl)selanyl)benzamide 19; Figure S24: Chromatograph of *N*-((*S*)-(-)-1-(1-napthyl)etyl)- 2-((2-oxopropyl)selanyl)benzamide 20; Figure S25: Chromatograph of *N*-((1*S*,2*R*)-(-)-cis-2-hydroksy-1-indanyl)- 2-((2-oxopropyl)selanyl)benzamide 21; Figure S26: Chromatograph of *N*-((1*S*,2*R*)-(-)-cis-2-hydroksy-1-indanyl)-2-((2-oxopropyl)selanyl)benzamide 22; Figure S27: Chromatograph of *N*-((1*S*,2*S*)-(+)-trans-2-hydroksy-1-indanyl)-2-((2-oxopropyl)selanyl)benzamide 23; Figure S28: Chromatograph of *N*-((1*R*,2*R*)-(-)-trans-2-hydroksy-1-indanyl)-2-((2-oxopropyl)selanyl)benzamide 24; Figure S29: Example of ^1^H NMR spectra for antioxidant Iwaoka test after reaction time 5 min, 15 min, 30 min, 60 min for compound **23**; Figure S30: Results of antioxidant activity measurement of integration from ^1^H NMR spectra after reaction time 5 min and 15 min for all samples and compound **11**–**24**; Figure S31: Results of antioxidant activity measurement of integration from ^1^H NMR spectra after reaction time 30 min and 60 min for all samples and compound **11**–**24**; Figure S32: Compound **11**/**12**—calibration curve; Figure S33: Compound **13**/**14**—calibration curve; Figure S34: Compound **15**/**16**—calibration curve; Figure S35: Compound **17**/**18**—calibration curve; Figure S36: Compound **19**/**20**—calibration curve; Figure S37: Compound **21**/**22**—calibration curve; Figure S38: Compound **23**/**24**—calibration curve; Figure S39: Standard curves for the relationship: absorbance = function (concentration TE) (A), % DPPH = function (concentration of TE) (B).
