# Supplementary material for: Synthesis of New Chiral β-Carbonyl Selenides with Antioxidant and Anticancer Activity Evaluation—Part I
Source: Materials (Basel). 2024 Feb 15;17(4):899. doi: 10.3390/ma17040899 (PMC10890689; doi:10.3390/ma17040899)
Supplement: Supplementary file 1 [file materials-17-00899-s001.zip › materials-2804284-supplementary.pdf]

Supplementary Materials

Synthesis of New Chiral β-Carbonyl Selenides with Antioxidant and Anticancer Activity Evaluation—Part I

Table of contents

1. 1H, 13C and 77Se NMR spectra of selenides 11-24, pages 2-22

2. HPLC analyses of selenides 11-24, pages 23-29

3. Antioxidant activity measurement, pages 30-31

4. DPPH test calibration curves, pages 32-34

1. NMR spectra of 2-((2-oxopropyl)selanyl) benzamides 11-24

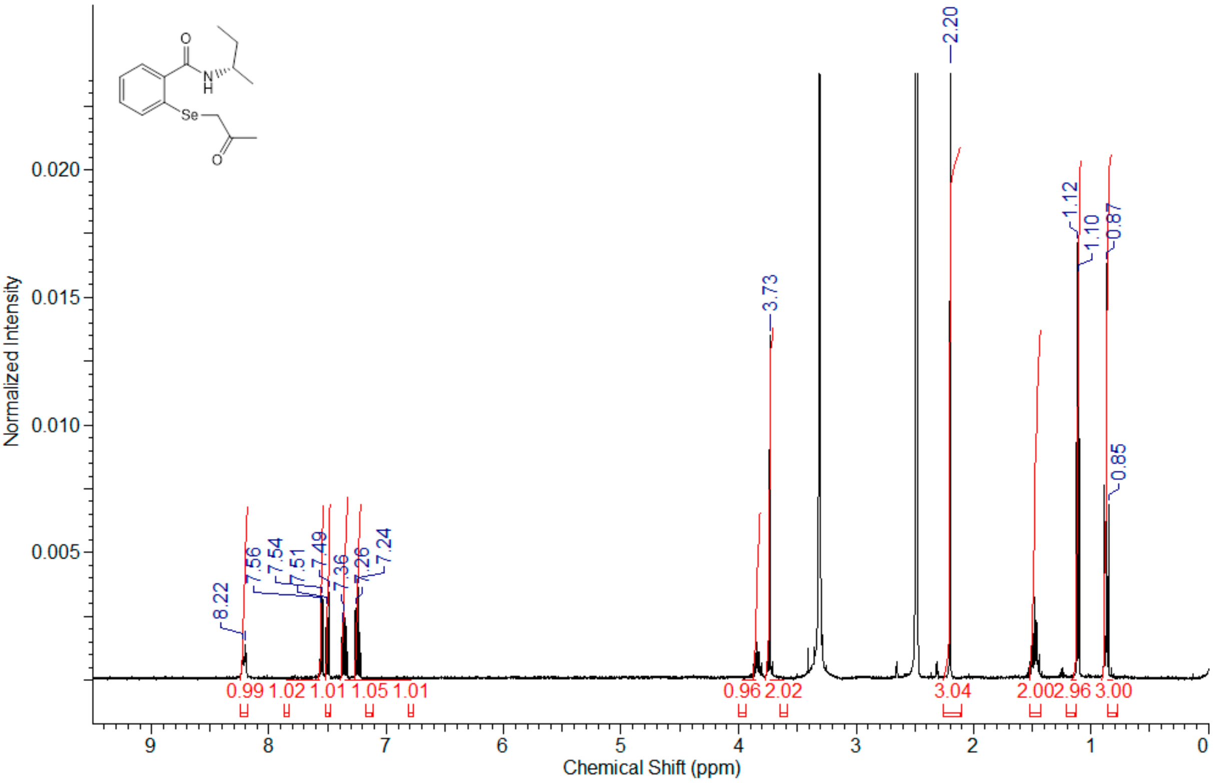
Normalized Intensity
0.020
0.015
0.010
0.005
Chemical Shift (ppm)
O
N
H
Se
O
8.22
7.56
7.54
7.51
7.49
7.36
7.26
7.24
3.73
2.20
1.12
1.10
0.87
0.85
0.99
1.02
1.01
1.05
1.01
0.96
2.02
3.04
2.00
2.96
3.00
9
8
7
6
5
4
3
2
1
0

(a)

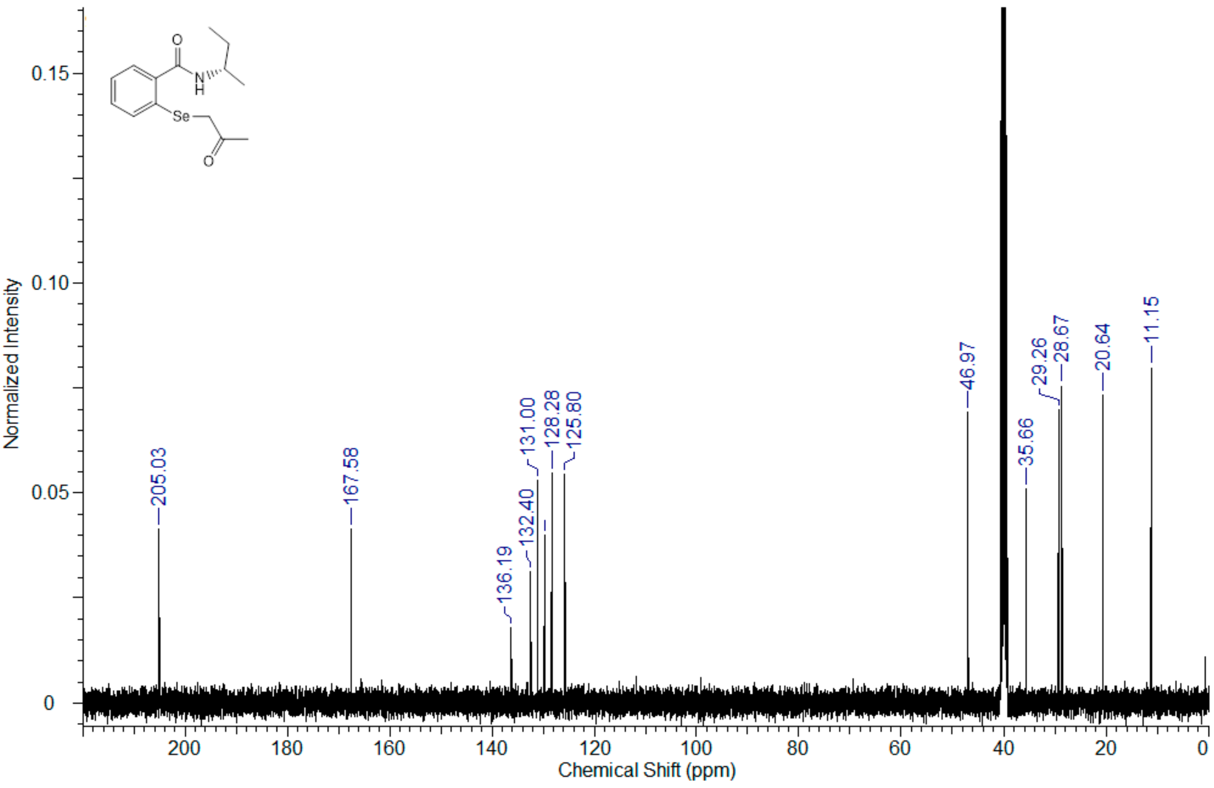
Normalized Intensity
0.15
0.10
0.05
0
Chemical Shift (ppm)
O
N
H
Se
O
205.03
167.58
136.19
132.40
131.00
128.28
125.80
46.97
35.66
29.26
28.67
20.64
11.15
200
180
160
140
120
100
80
60
40
20
0

(b)

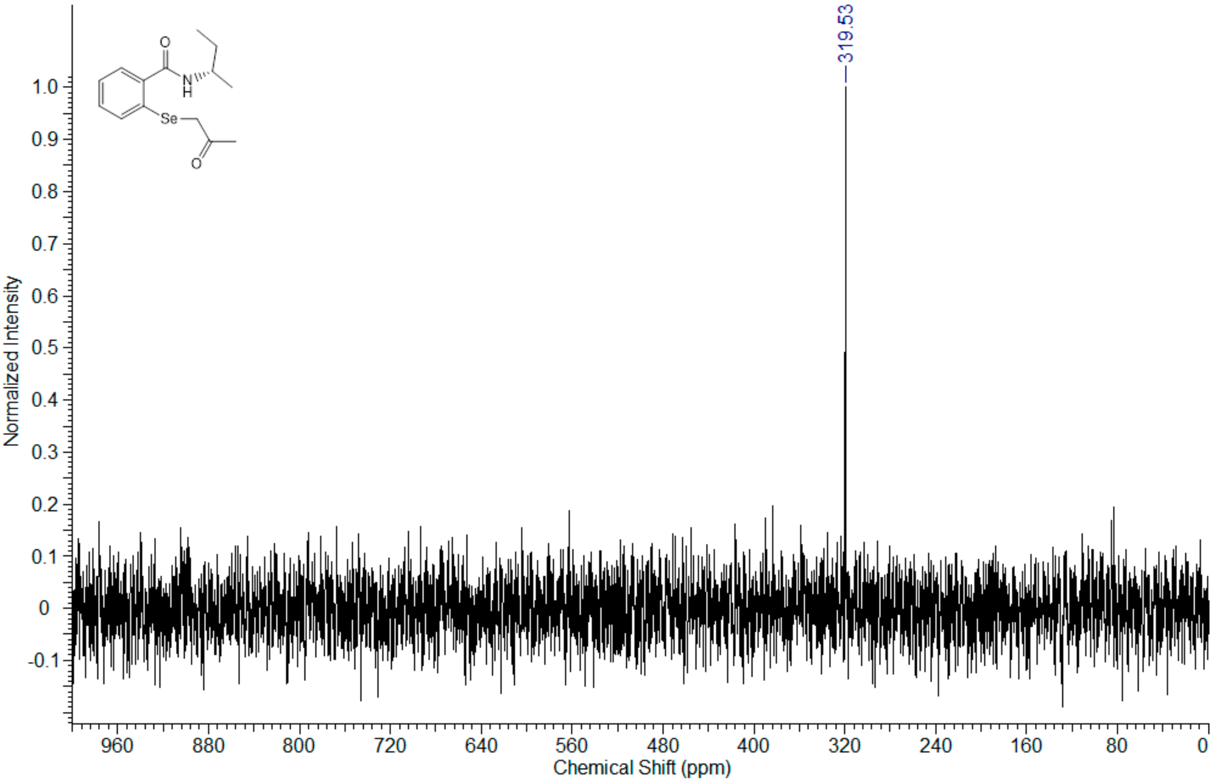

(c)

Figure S1. (**a**) 1H NMR, (**b**) 13C NMR, and (**c**) 77Se NMR spectra of *N*-((*S*)-(+)-*sec*-butyl)-2-((2-oxopropyl)selanyl)benzamide **11**.

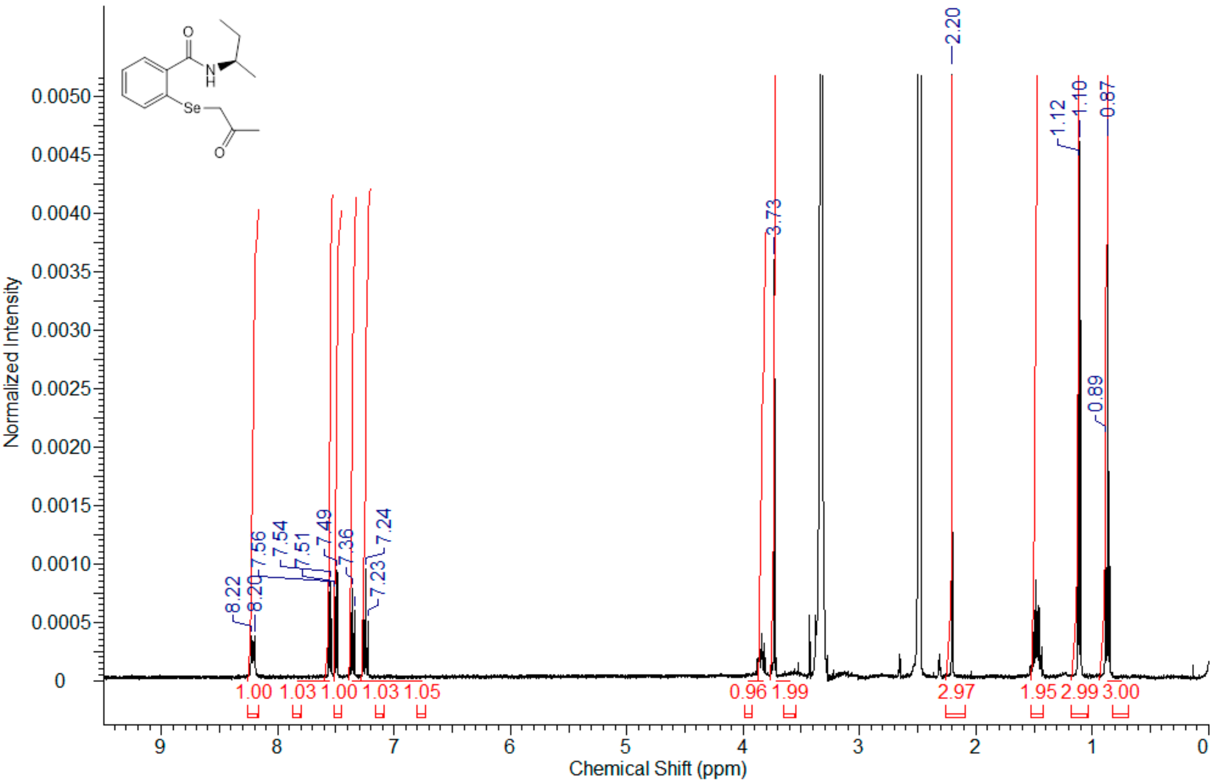

(a)

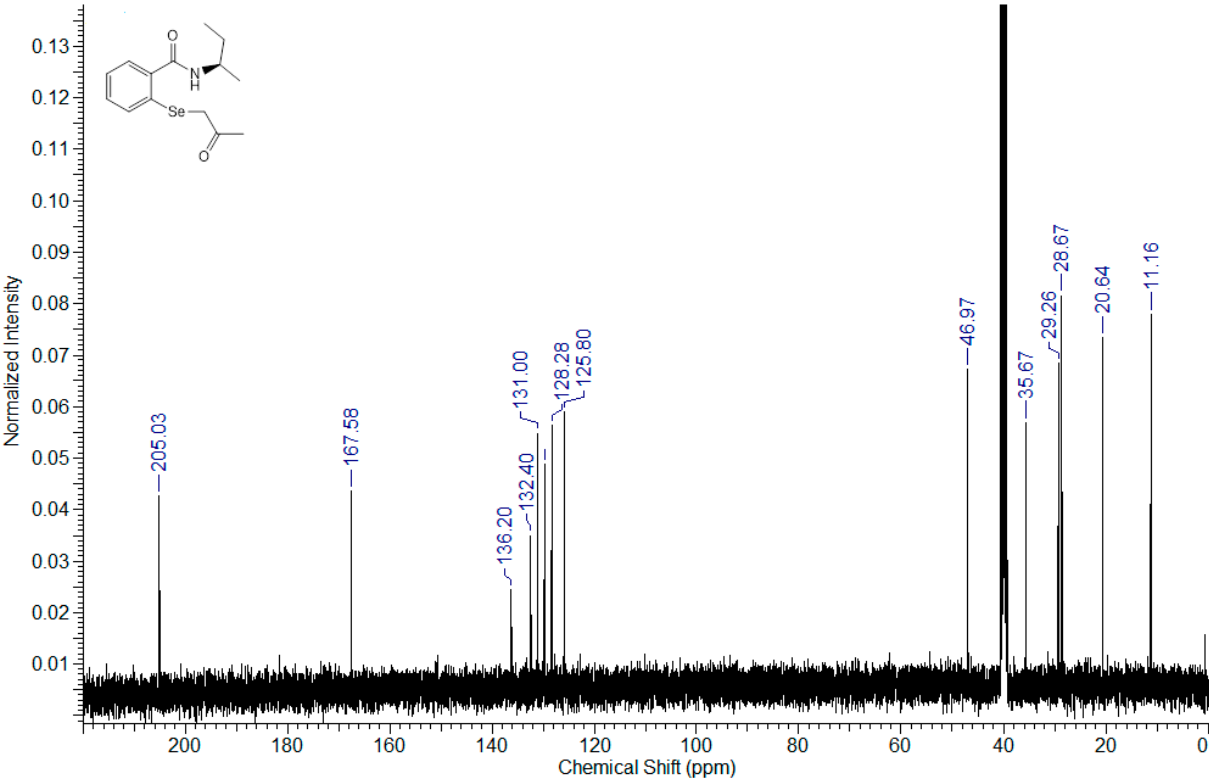

(b)

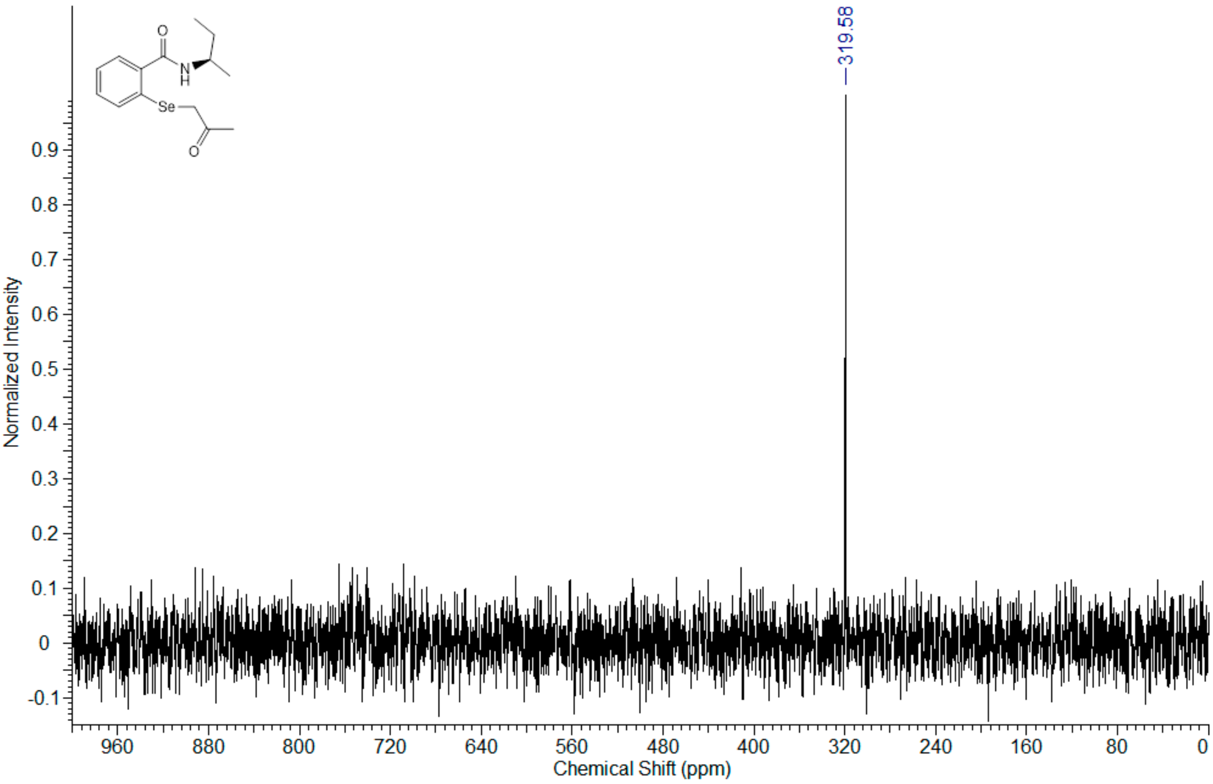

(c)

Figure S2. (**a**) 1H NMR, (**b**) 13C NMR, and (**c**) 77Se NMR spectra of *N*-((*R*)-(-)-*sec*-butyl)-2-((2-oxopropyl)selanyl)benzamide **12**.

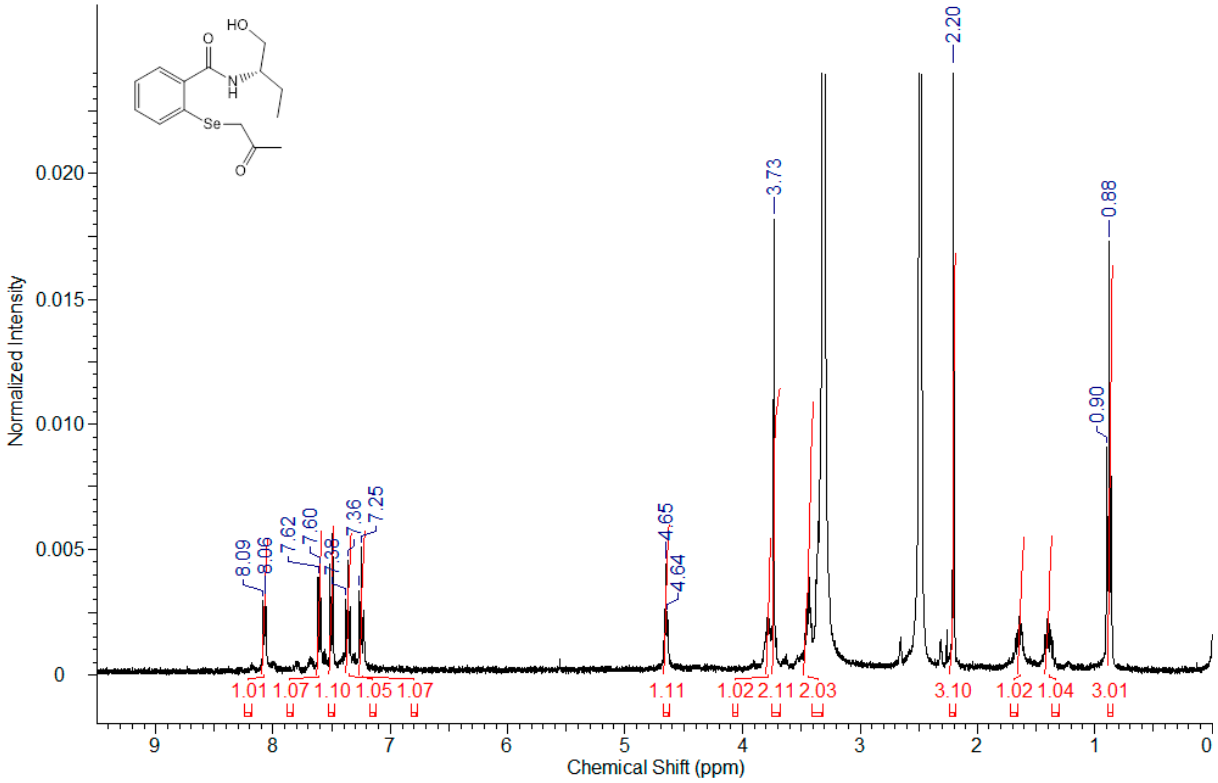
HO
O
N
H
Se
O
Normalized Intensity
Chemical Shift (ppm)
8.09
8.06
7.62
7.60
7.38
7.36
7.25
4.65
4.64
3.73
2.20
0.90
0.88
1.01
1.07
1.10
1.05
1.07
1.11
1.02
2.11
2.03
3.10
1.02
1.04
3.01

(a)

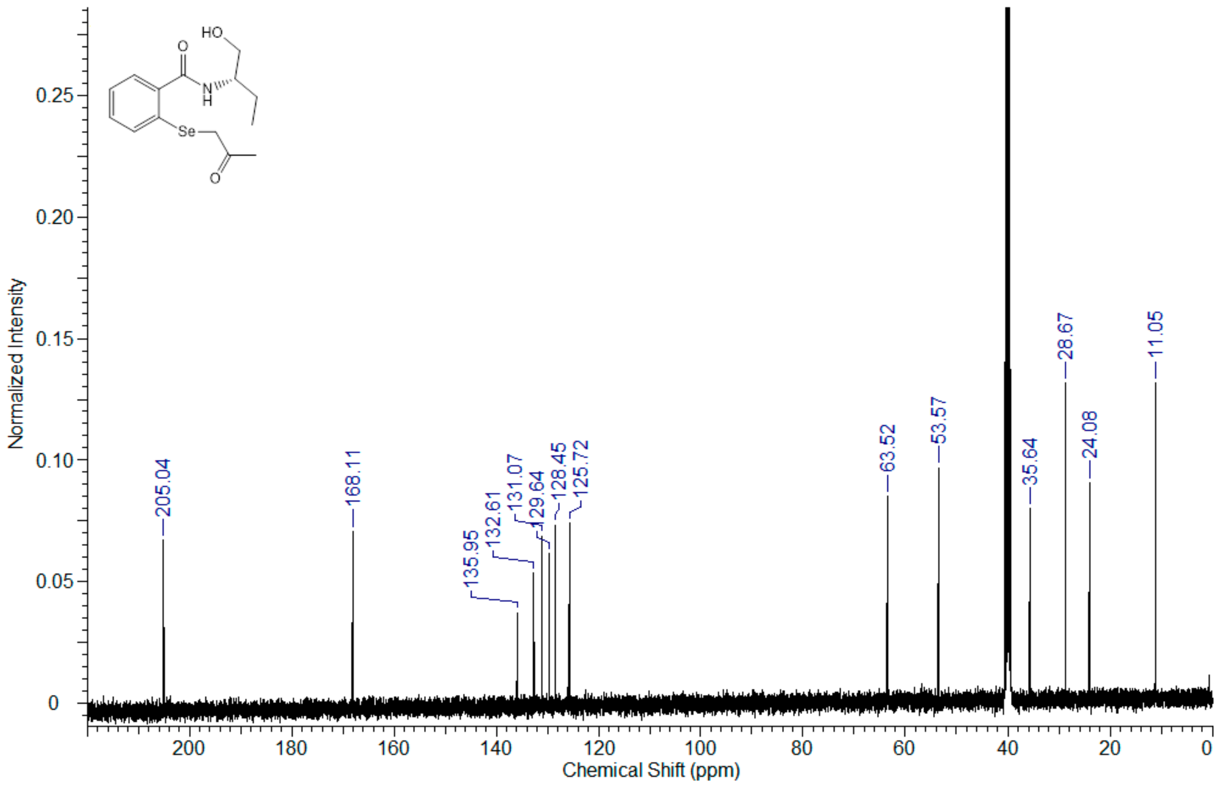
HO
O
N
H
Se
O
Normalized Intensity
Chemical Shift (ppm)
205.04
168.11
135.95
132.61
131.07
129.64
128.45
125.72
63.52
53.57
35.64
28.67
24.08
11.05

(b)

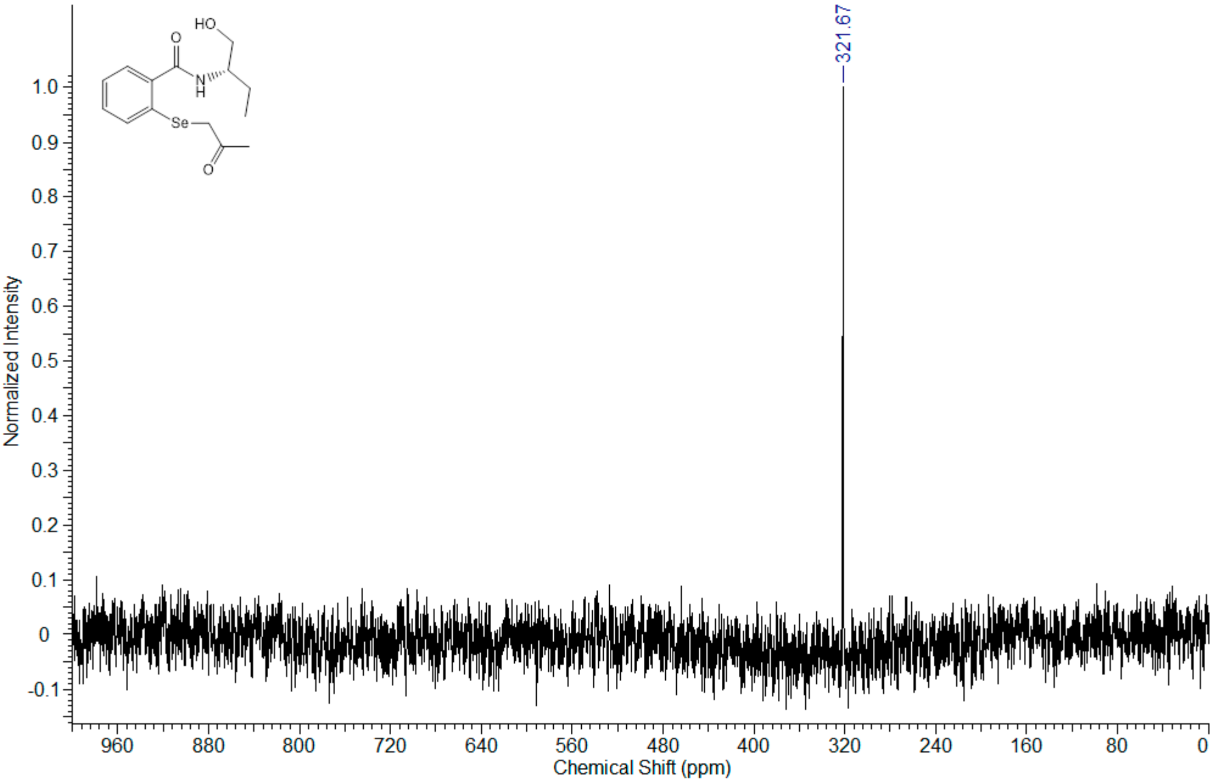

(c)

Figure S3. (**a**) 1H NMR, (**b**) 13C NMR, and (**c**) 77Se NMR spectra of *N*-((*S*)-(+)-1-hydroksy-2-butanyl)-2-((2-oxopropyl)selanyl)benzamide **13**.

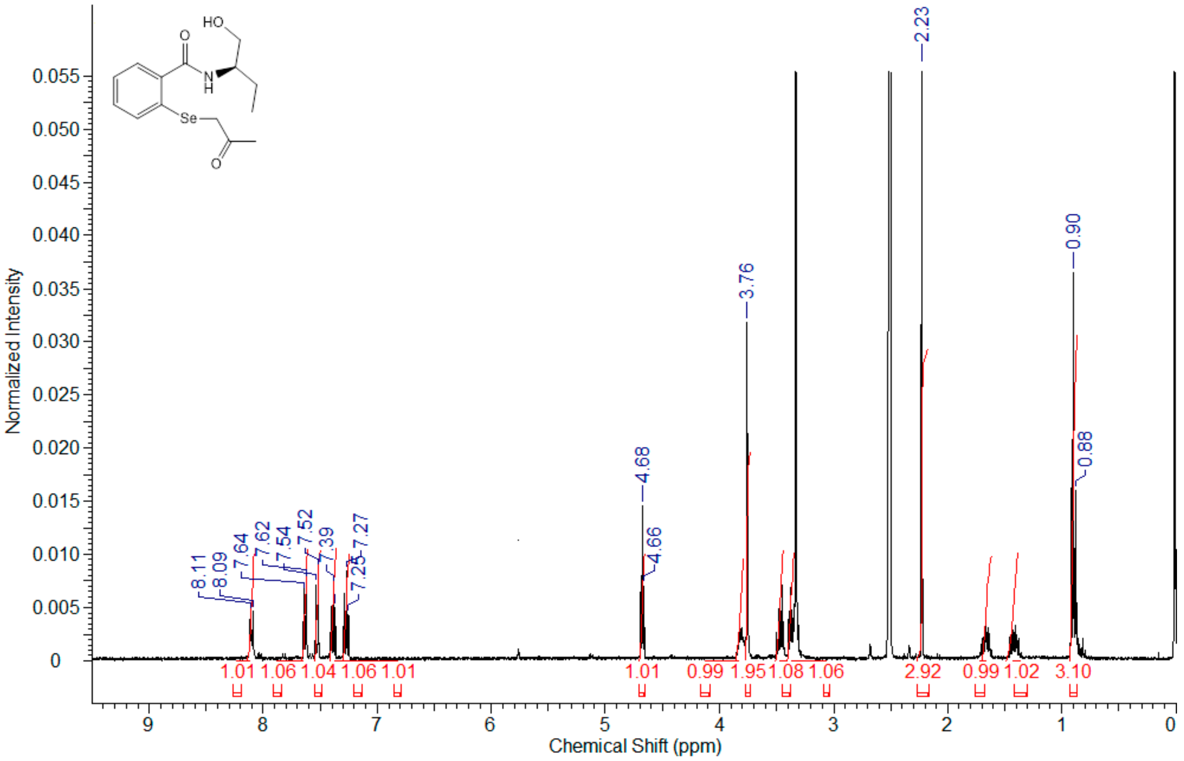

(a)

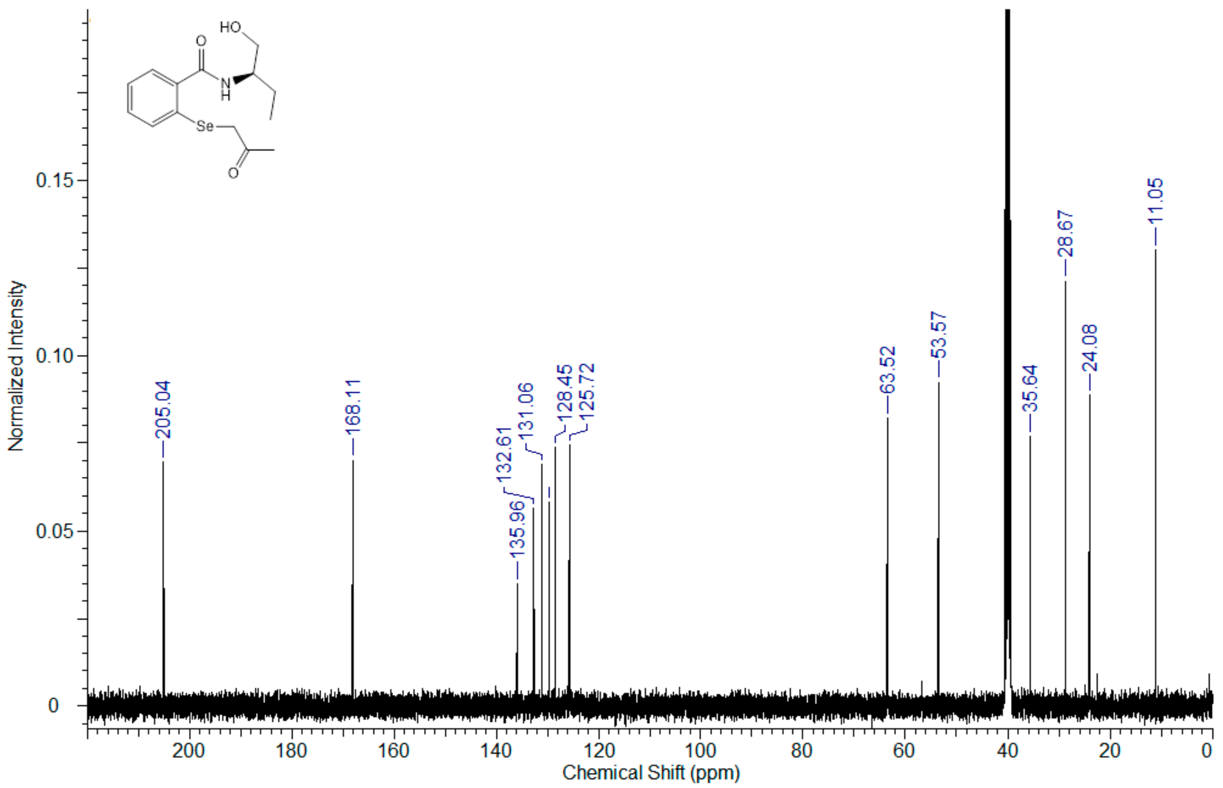

(b)

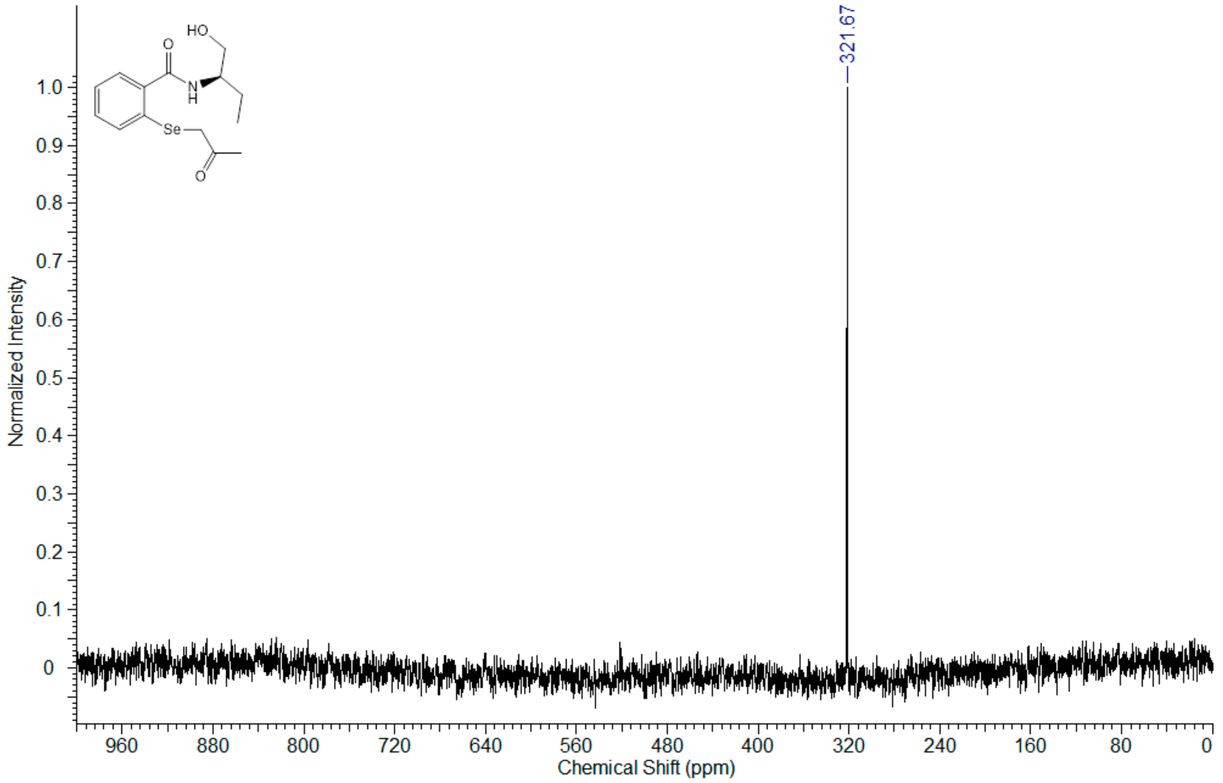

(c)

Figure S4. (**a**) 1H NMR, (**b**) 13C NMR, and (**c**) 77Se NMR spectra of *N*-((*R*)-(-)-1-hydroksy-2-butanyl)-2-((2-oxopropyl)selanyl)benzamide **14**.

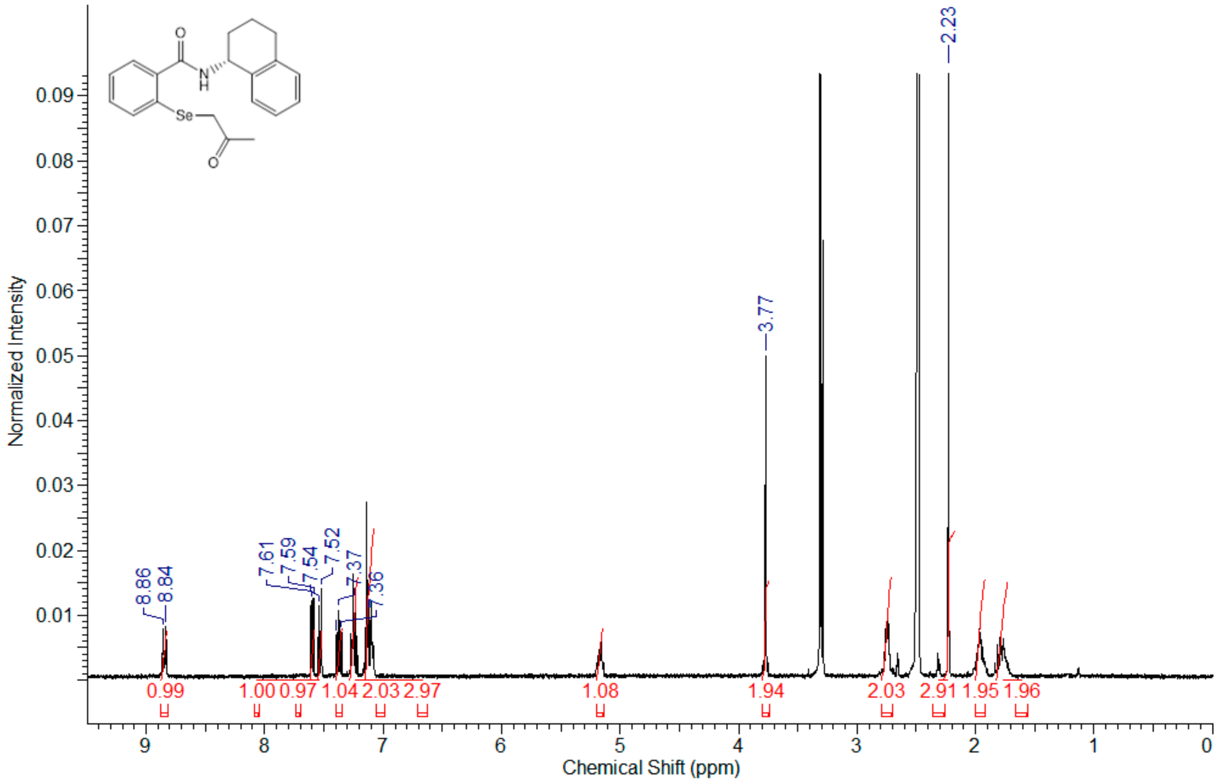

Normalized Intensity
Chemical Shift (ppm)
8.86
8.84
7.61
7.59
7.54
7.52
7.37
7.36
3.77
2.23
0.99
1.00
0.97
1.04
2.03
2.97
1.08
1.94
2.03
2.91
1.95
1.96
Se
NH

(a)

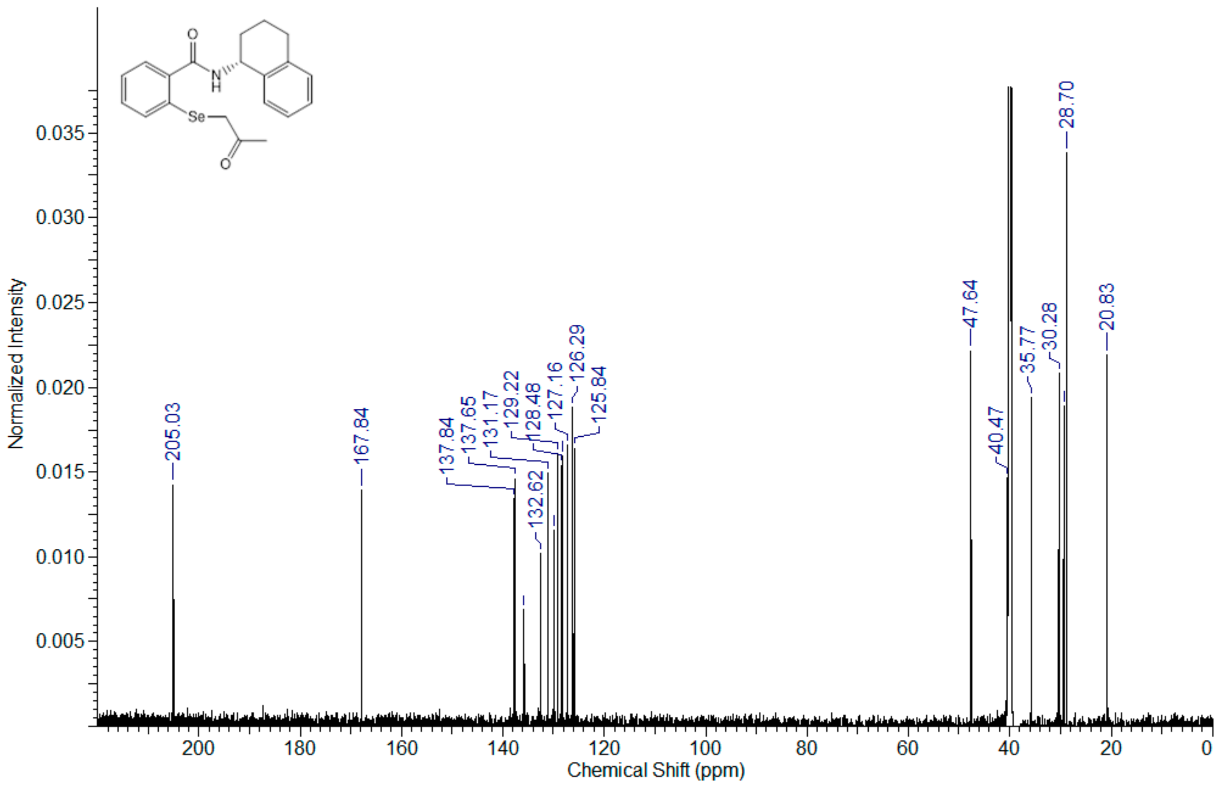

Normalized Intensity
Chemical Shift (ppm)
205.03
167.84
137.84
137.65
131.17
129.22
128.48
127.16
126.29
125.84
132.62
47.64
40.47
35.77
30.28
28.70
20.83
Se
NH

(b)

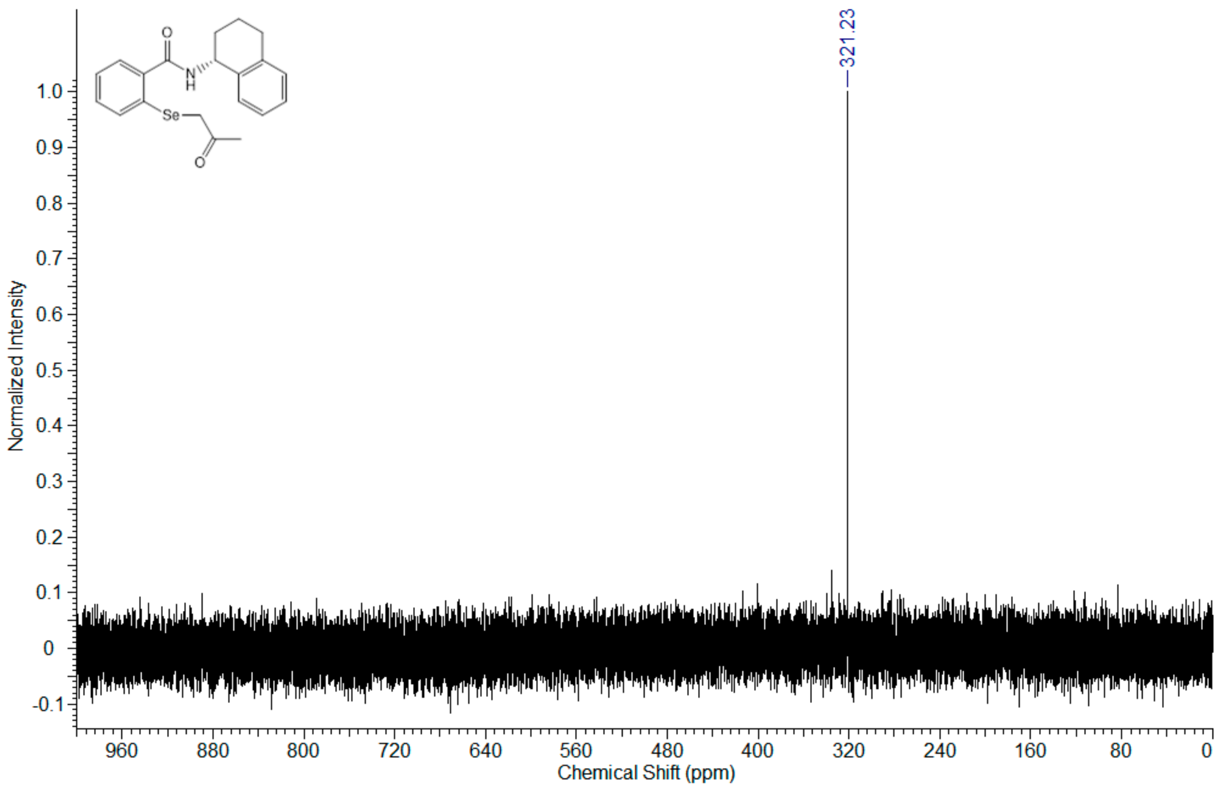

(c)

Figure S5. (**a**) 1H NMR, (**b**) 13C NMR, and (**c**) 77Se NMR spectra of *N*-((*R*)-(-)-1,2,3,4-tetrahydro-1-napthyl) 2-((2-oxopropyl)selanyl)benzamide **15**.

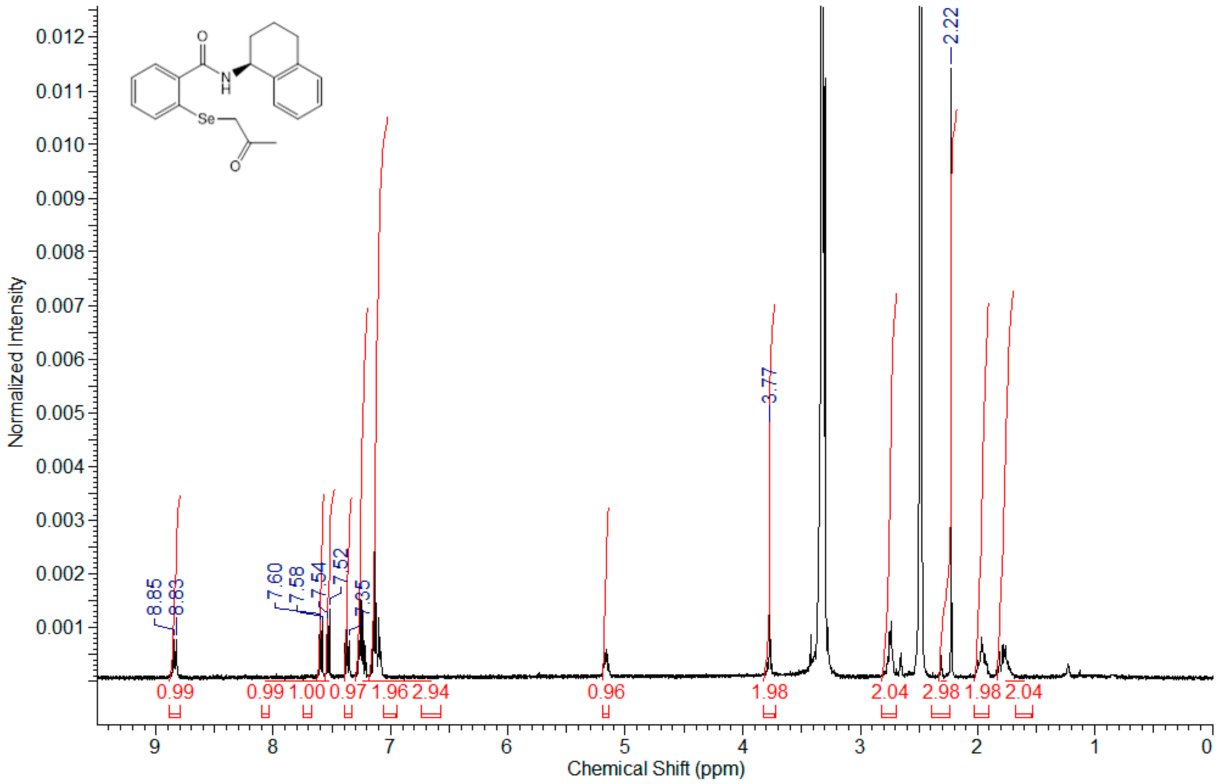

(a)

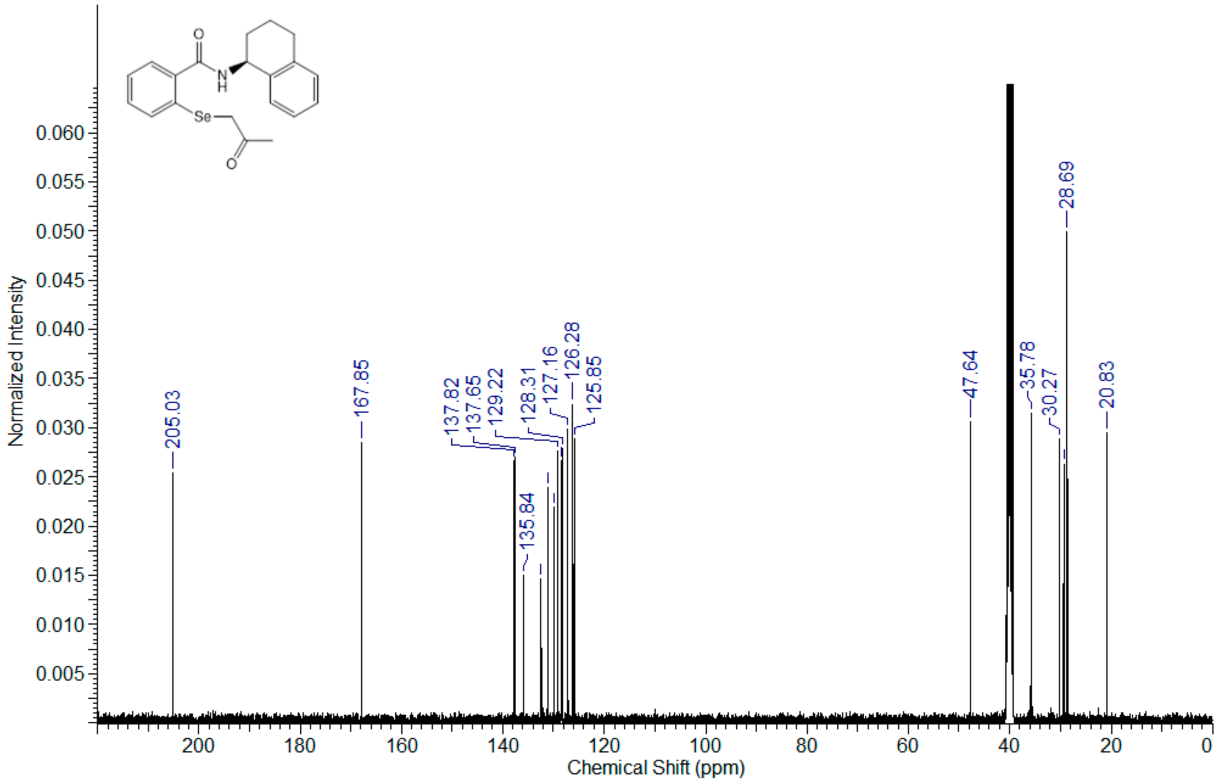

(b)

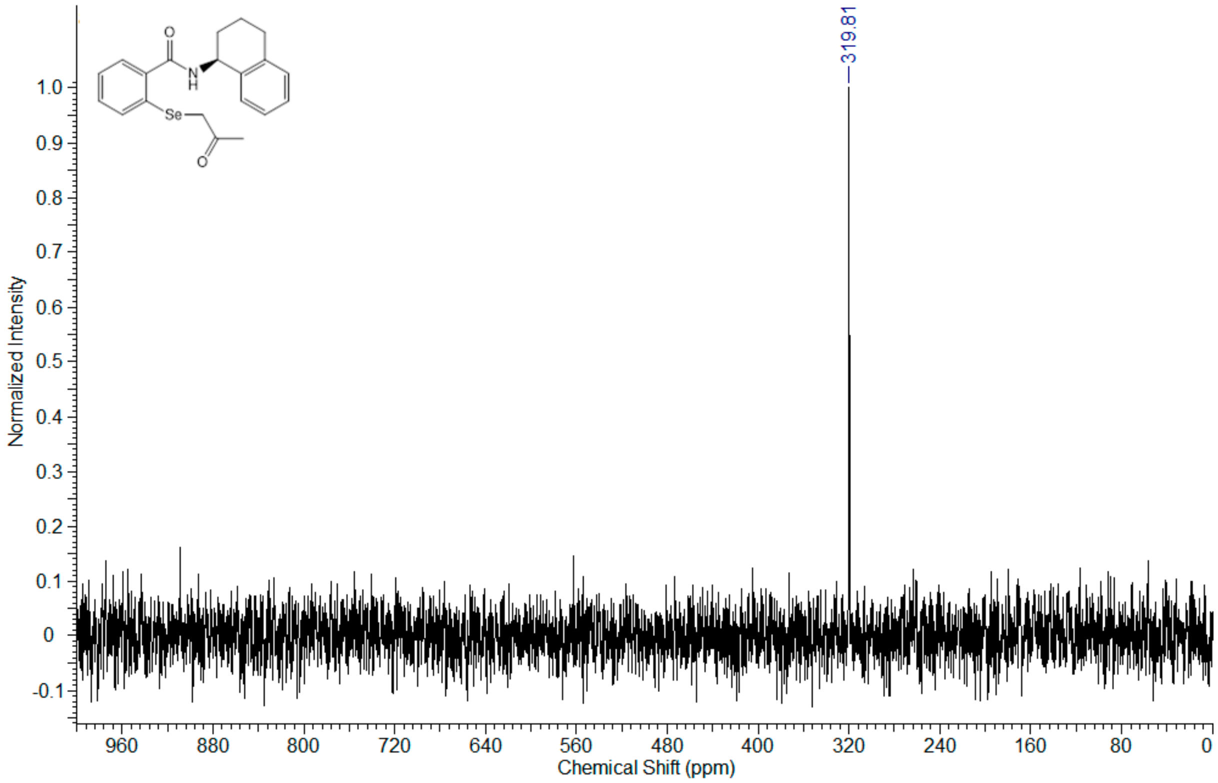

(C)

Figure S6. (**a**) 1H NMR, (**b**) 13C NMR, and (**c**) 77Se NMR spectra of *N*-((*S*)-(+)-1,2,3,4-tetrahydro-1-napthyl) 2-((2-oxopropyl)selanyl)benzamide **16**.

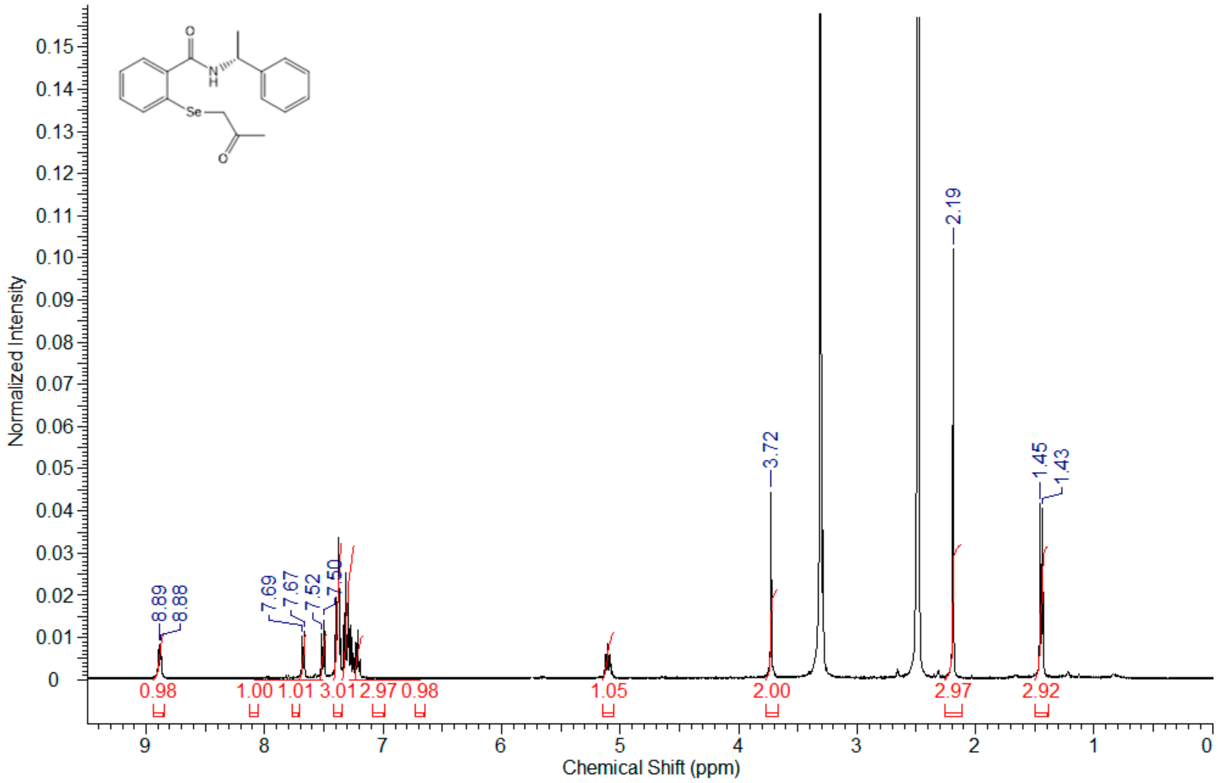

Normalized Intensity
Chemical Shift (ppm)
8.89
8.88
7.69
7.67
7.52
7.50
3.72
2.19
1.45
1.43
0.98
1.00
1.01
3.01
2.97
0.98
1.05
2.00
2.97
2.92
Se
O
N
H

(a)

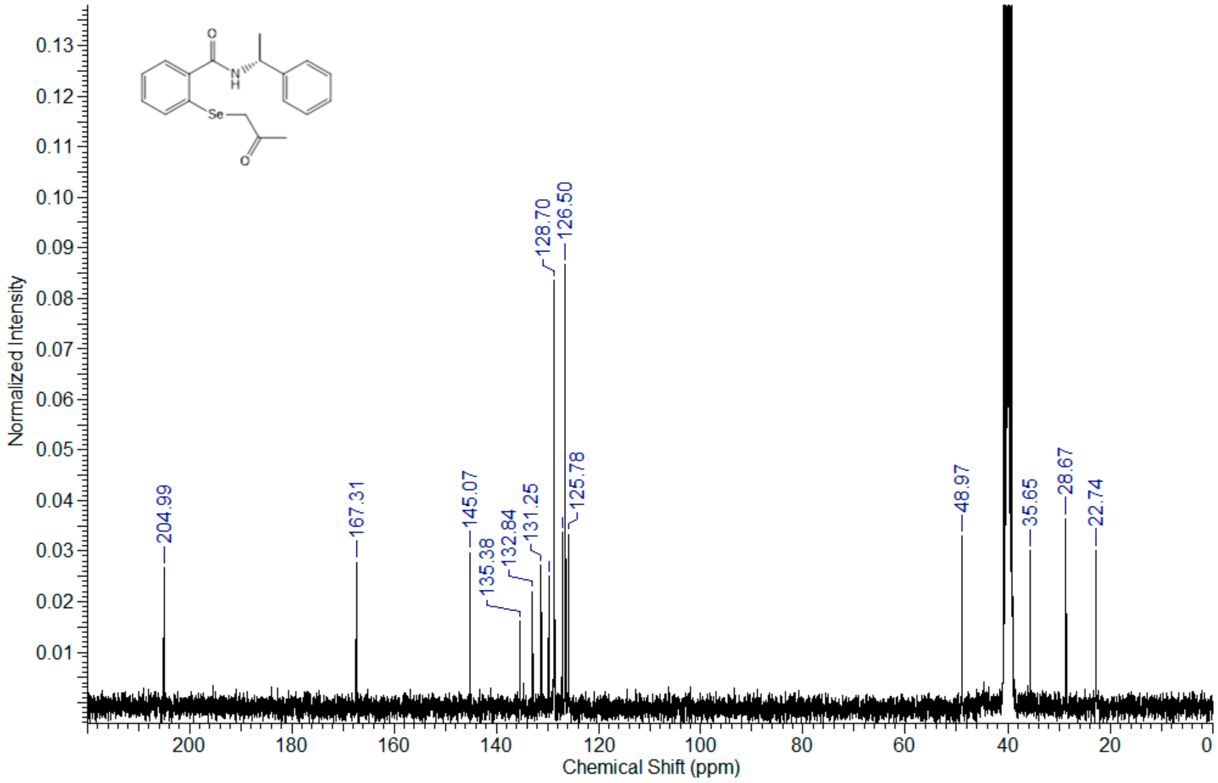

Normalized Intensity
Chemical Shift (ppm)
204.99
167.31
145.07
135.38
132.84
131.25
128.70
126.50
125.78
48.97
35.65
28.67
22.74
Se
O
N
H

(b)

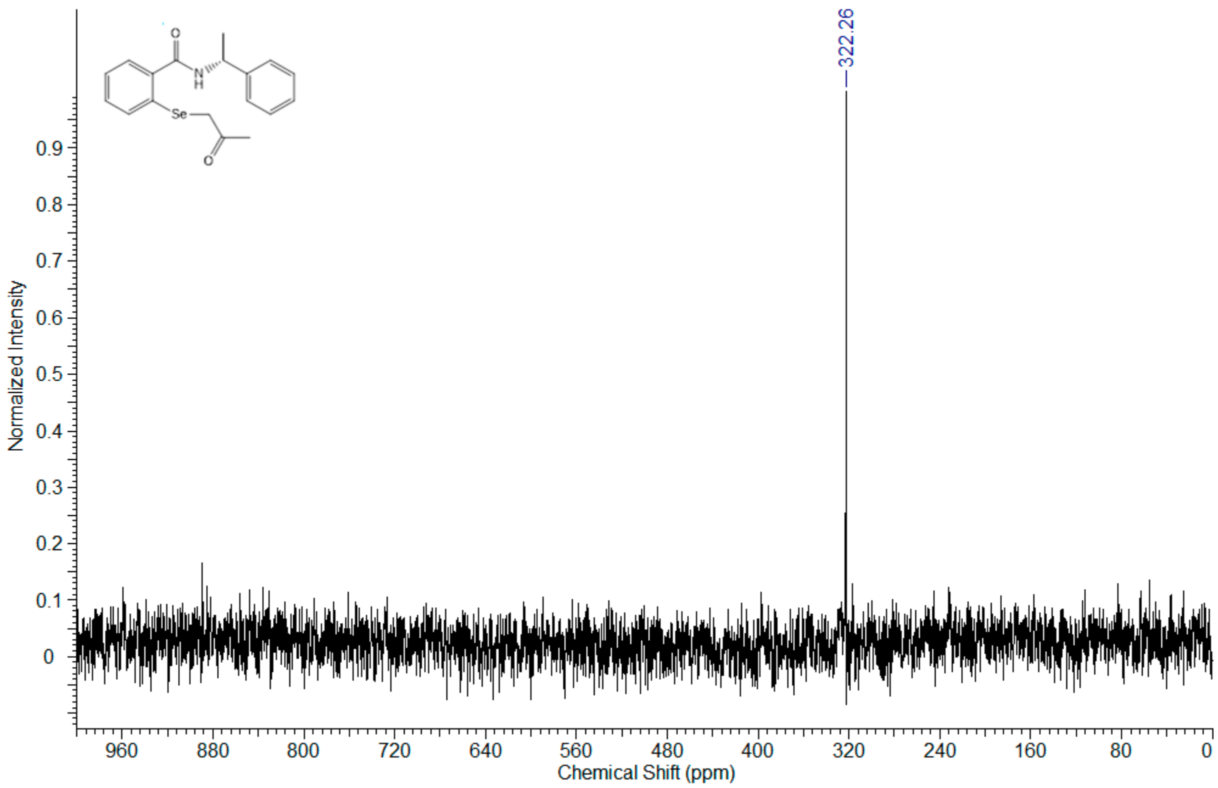

(c)

Figure S7. (**a**) 1H NMR, (**b**) 13C NMR, and (**c**) 77Se NMR spectra of *N*-((*R*)-(+)-α-metylbenzyl)-2-((2-oxopropyl)selanyl)benzamide **17**.

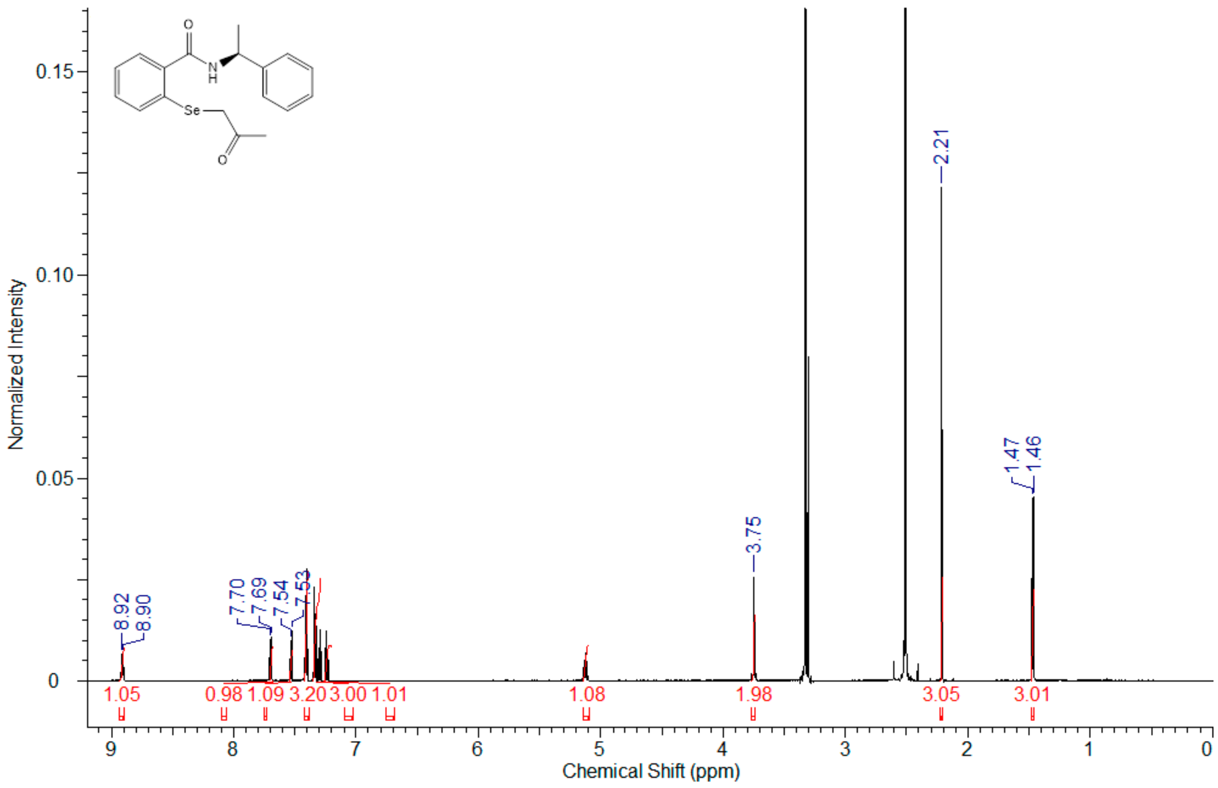

(a)

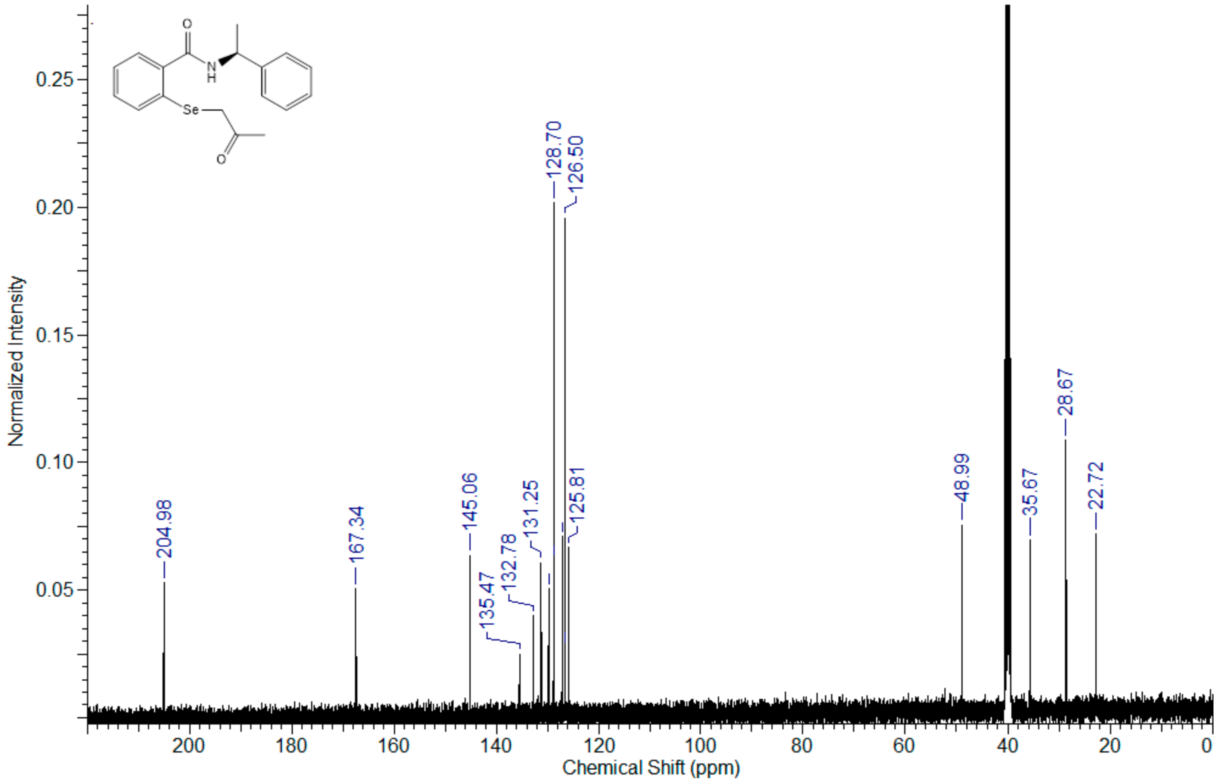

(b)

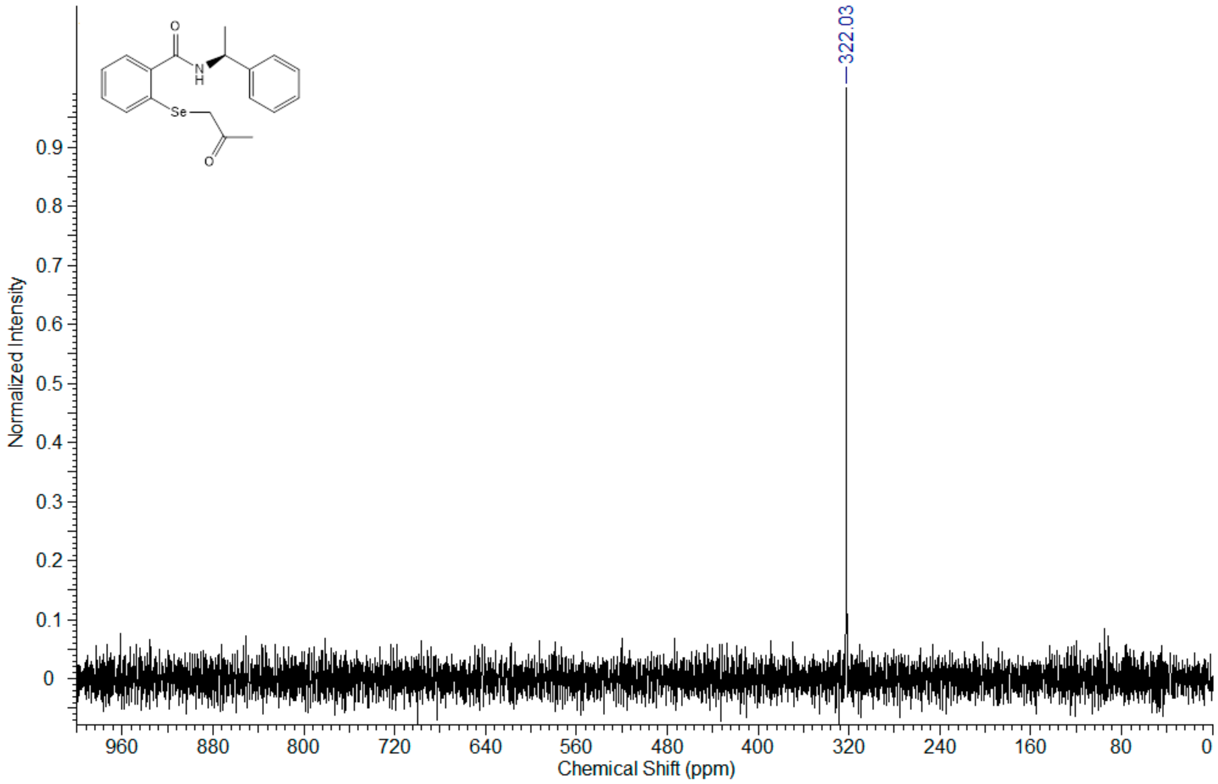

(c)

Figure S8. (**a**) 1H NMR, (**b**) 13C NMR, and (**c**) 77Se NMR spectra of *N*-((*S*)-(-)-α-metylbenzyl)-2-((2-oxopropyl)selanyl)benzamide **18**.

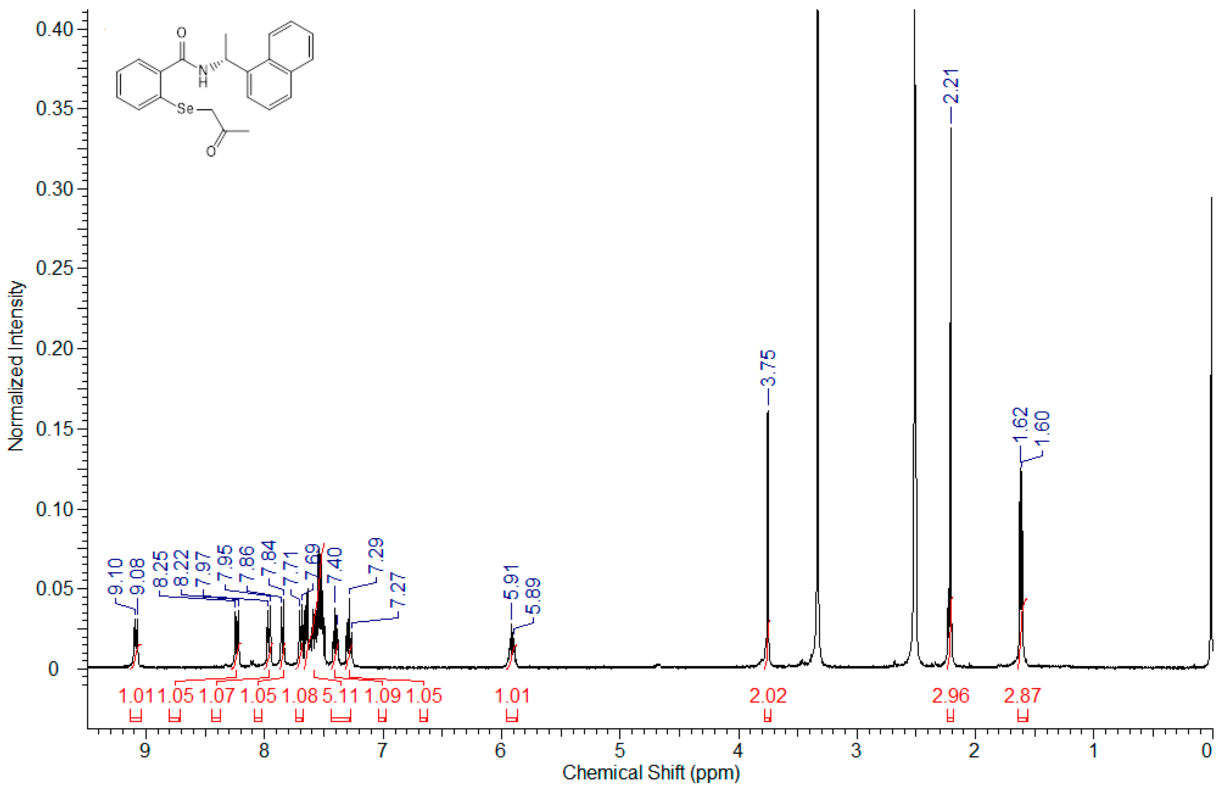

Normalized Intensity
Chemical Shift (ppm)
9.10
9.08
8.25
8.22
7.97
7.95
7.86
7.84
7.71
7.69
7.40
7.29
7.27
5.91
5.89
3.75
2.21
1.62
1.60
1.01
1.05
1.07
1.05
1.08
5.11
1.09
1.05
1.01
2.02
2.96
2.87

(a)

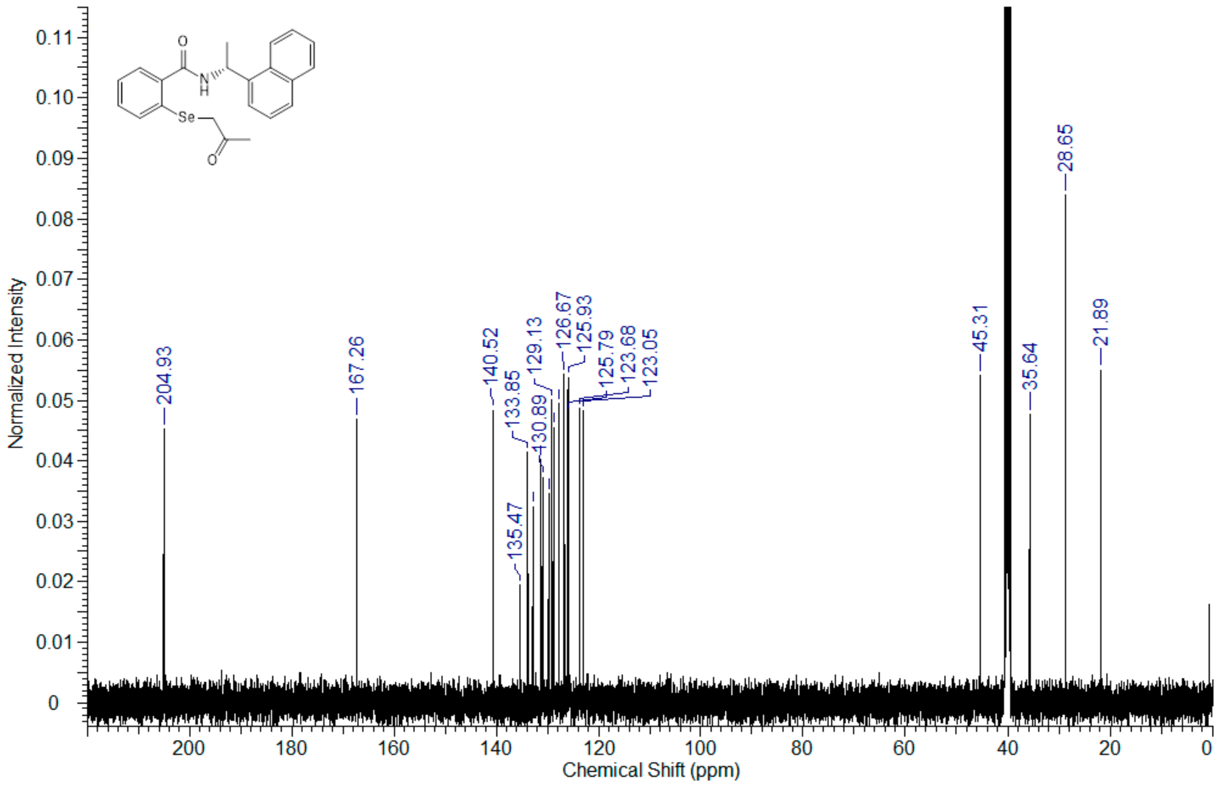

Normalized Intensity
Chemical Shift (ppm)
204.93
167.26
140.52
135.47
133.85
130.89
129.13
126.67
125.93
125.79
123.68
123.05
45.31
35.64
28.65
21.89

(b)

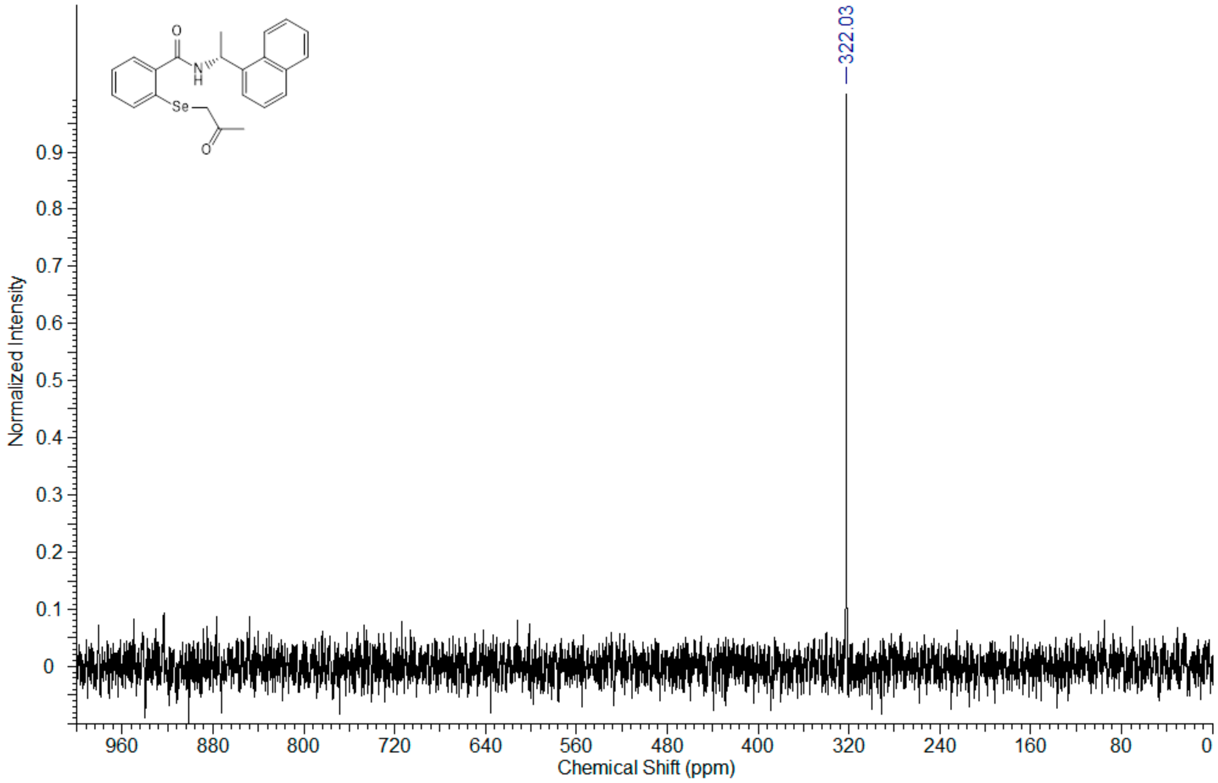

(c)

Figure S9. (**a**) 1H NMR, (**b**) 13C NMR, and (**c**) 77Se NMR spectra of *N*-((*S*)-(-)-1-(1-napthyl)etyl)-2-((2-oxopropyl)selanyl)benzamide **19**.

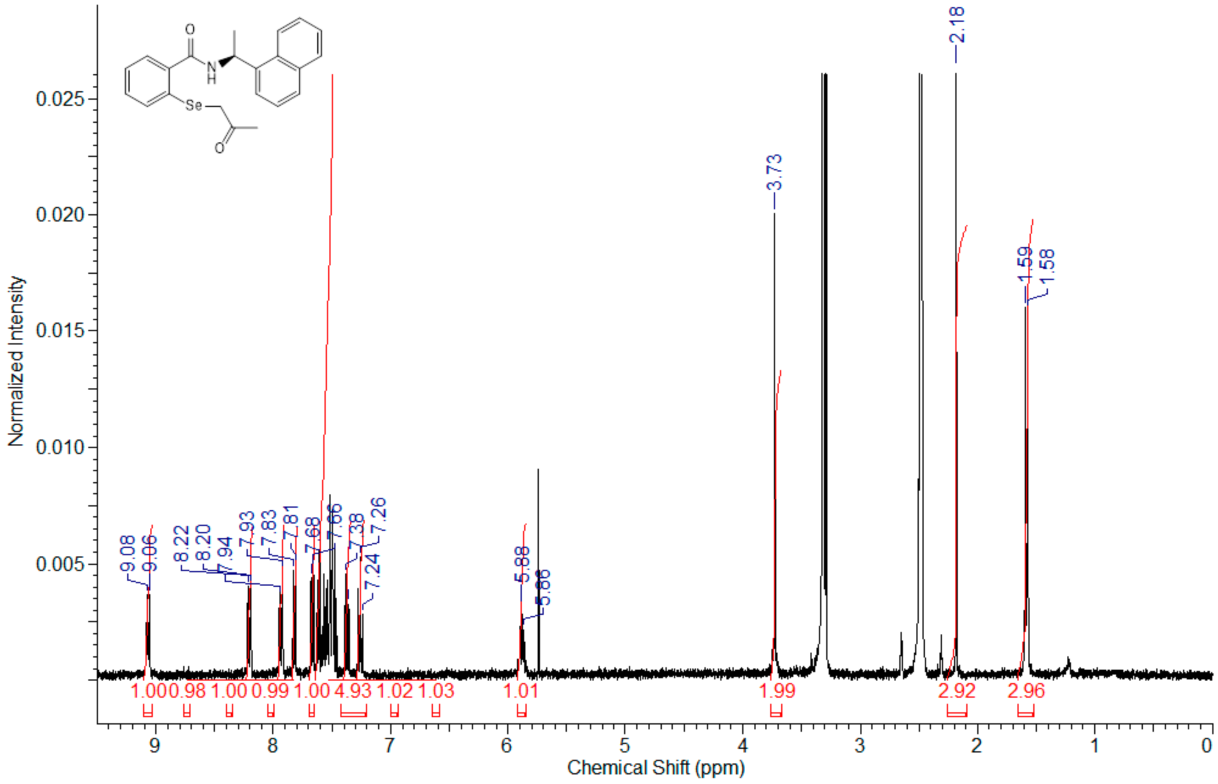

(a)

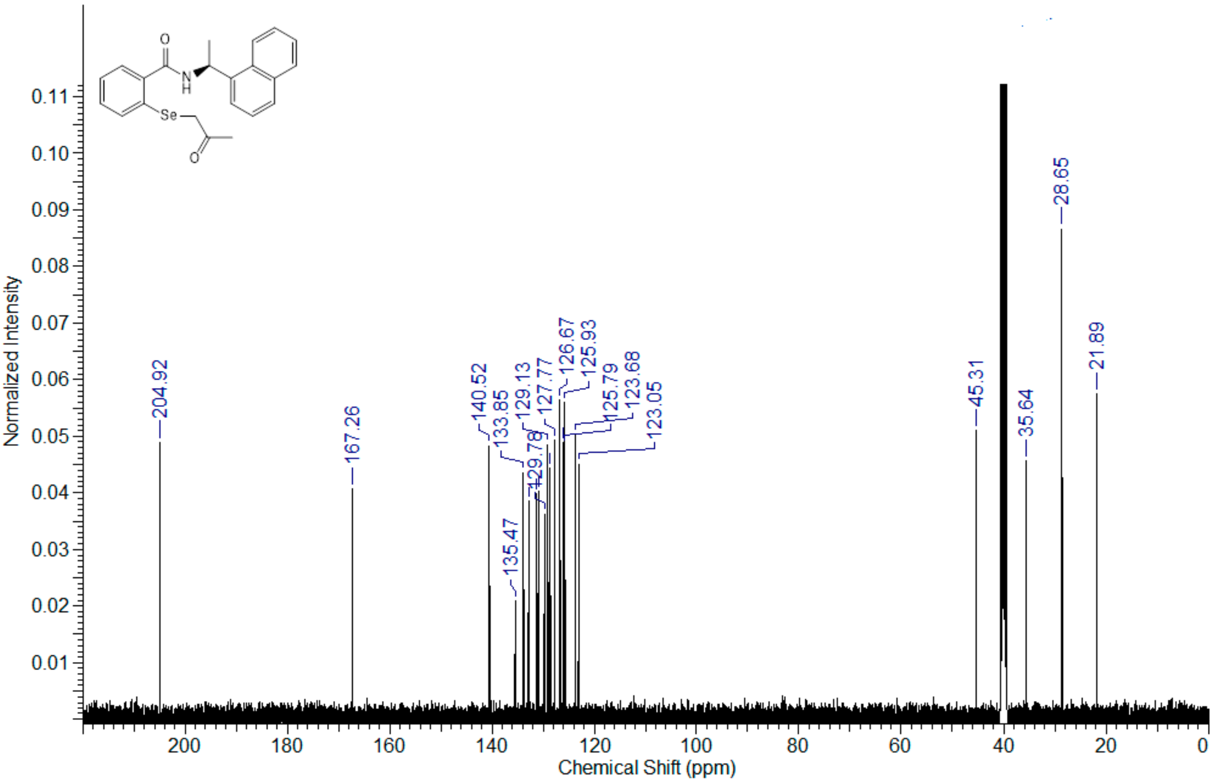

(b)

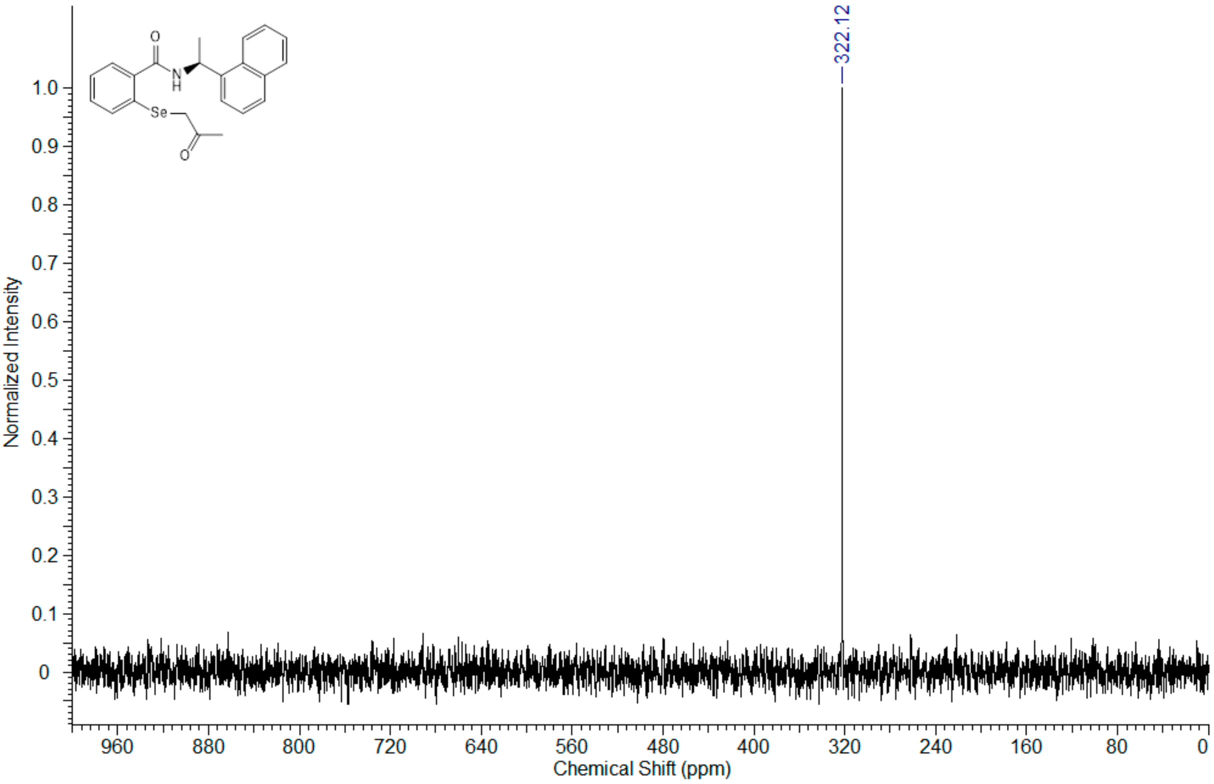

(c)

Figure S10. (**a**) 1H NMR, (**b**) 13C NMR, and (**c**) 77Se NMR spectra of *N*-((*R*)-(+)-1-(1-napthyl)etyl)-2-((2-oxopropyl)selanyl)benzamide **20**.

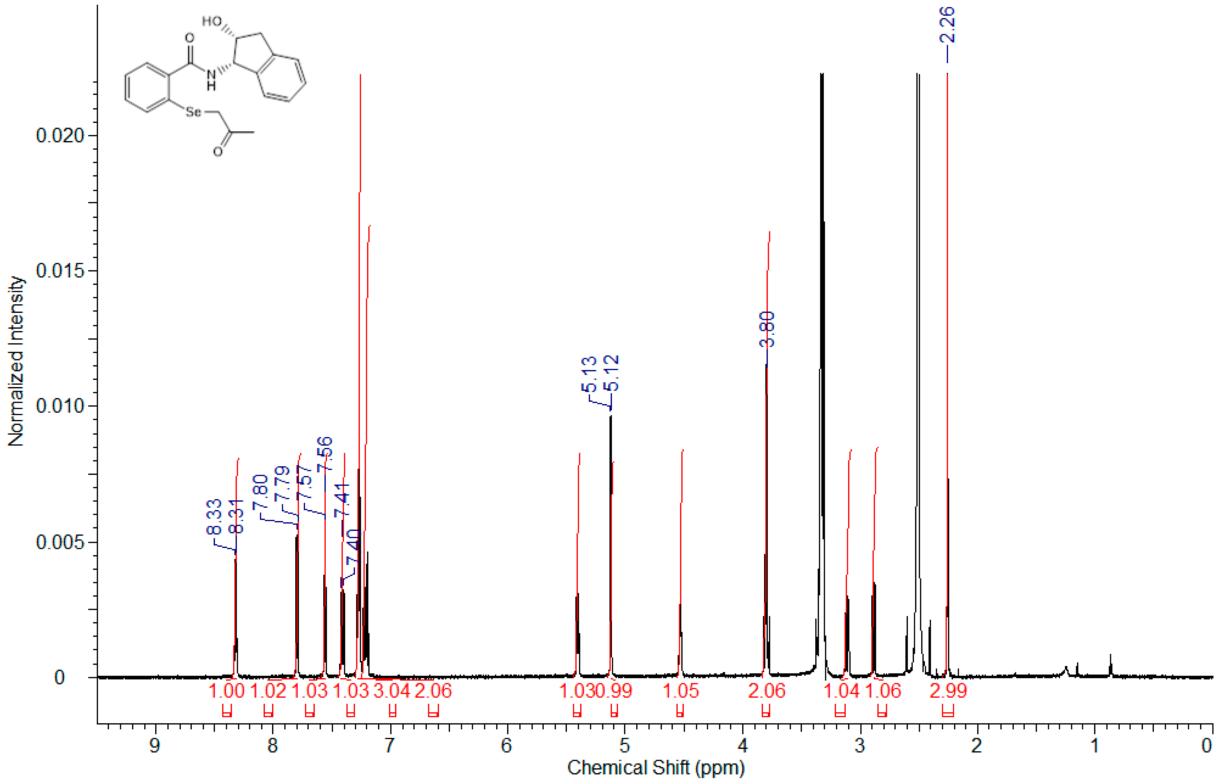
HO
O
N
H
Se
O
0.020
0.015
0.010
0.005
0
Normalized Intensity
8.33
8.31
7.80
7.79
7.57
7.56
7.41
7.40
5.13
5.12
3.80
2.26
1.00
1.02
1.03
1.03
3.04
2.06
1.03
0.99
1.05
2.06
1.04
1.06
2.99
9
8
7
6
5
4
3
2
1
0
Chemical Shift (ppm)

(a)

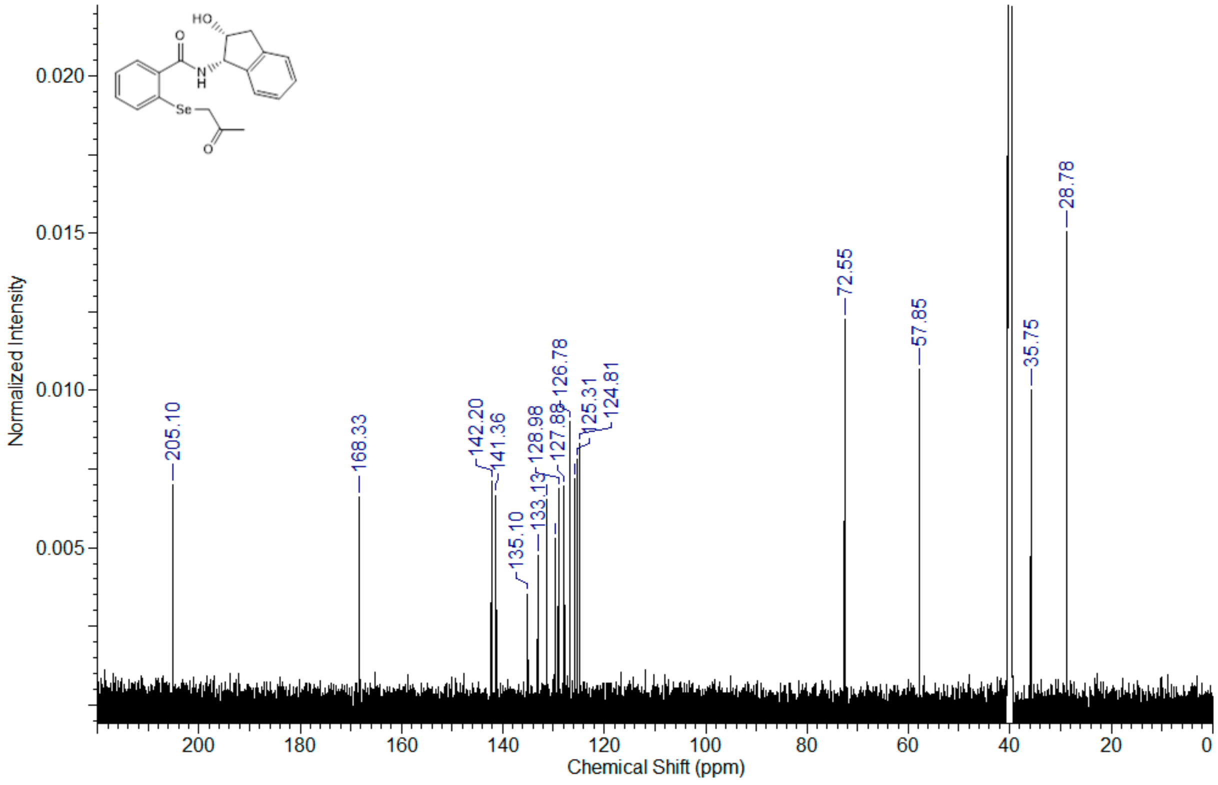
HO
O
N
H
Se
O
0.020
0.015
0.010
0.005
Normalized Intensity
205.10
168.33
142.20
141.36
135.10
133.13
128.98
127.89
126.78
125.31
124.81
72.55
57.85
35.75
28.78
200
180
160
140
120
100
80
60
40
20
0
Chemical Shift (ppm)

(b)

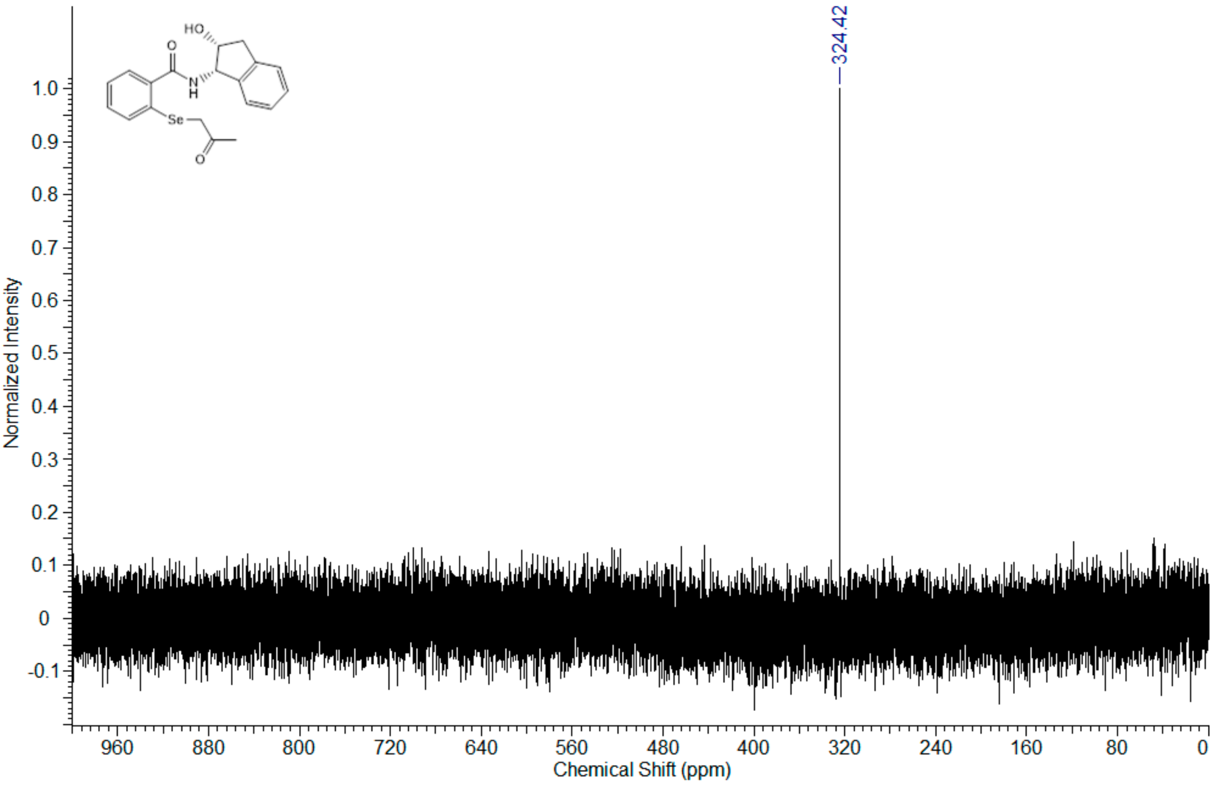

(c)

Figure S11. (**a**) 1H NMR, (**b**) 13C NMR, and (**c**) 77Se NMR spectra of *N*-((*1S,2R*)-(-)-*cis*-2-hydroksy-1-indanyl)- 2-((2-oxopropyl)selanyl)benzamide **21**.

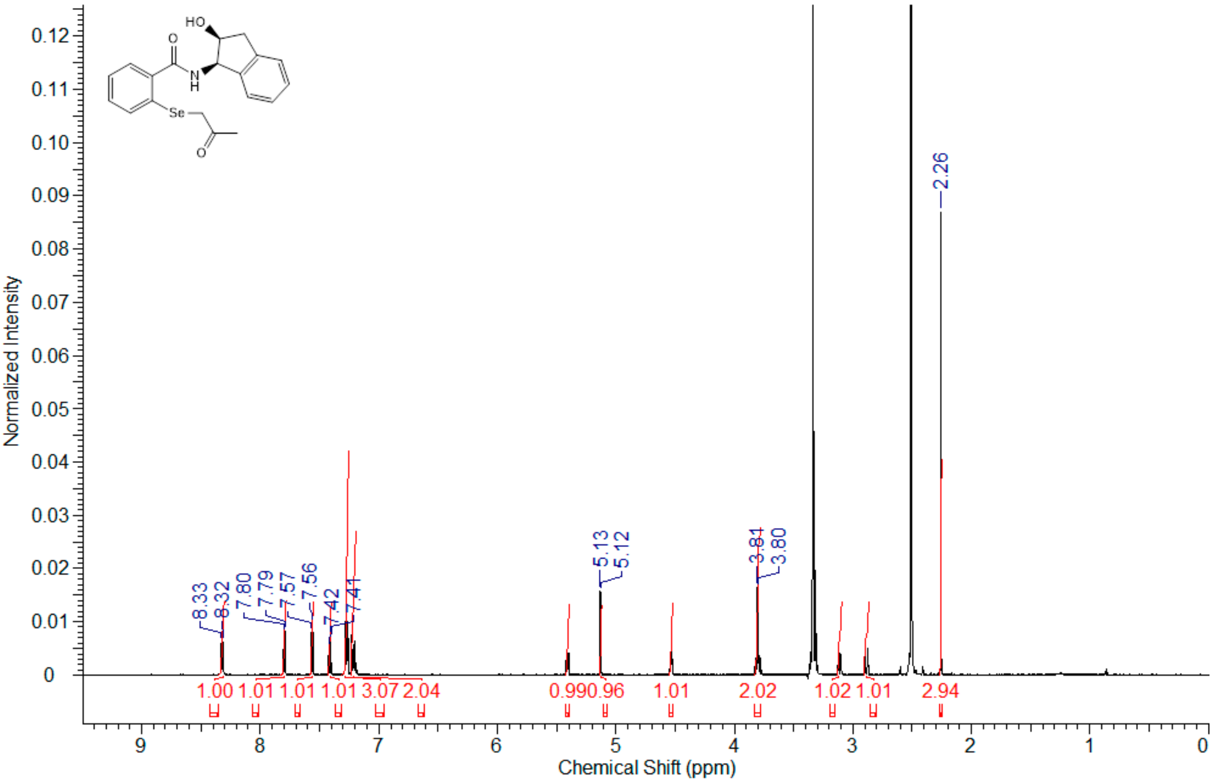

(a)

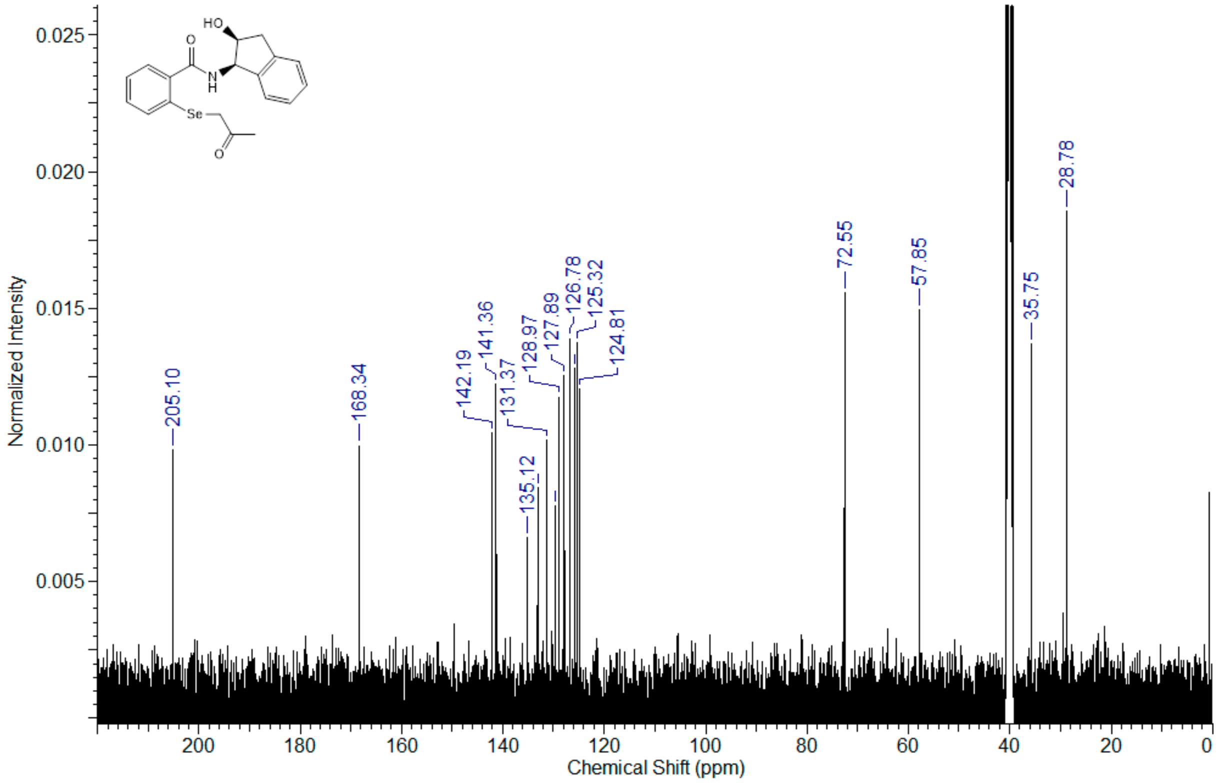

(b)

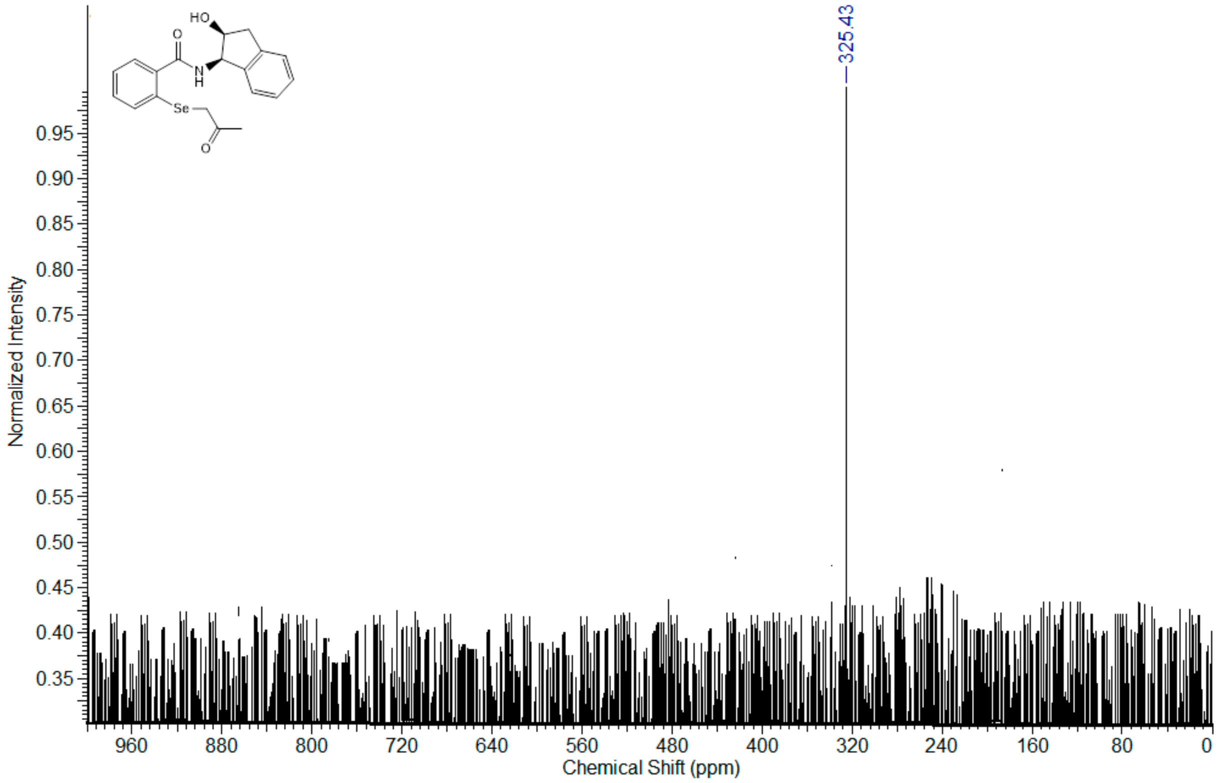

(c)

Figure S12. (**a**) 1H NMR, (**b**) 13C NMR, and (**c**) 77Se NMR spectra of *N*-((*1R,2S*)-(+)-*cis*-2-hydroksy-1-indanyl)- 2-((2-oxopropyl)selanyl)benzamide **22**.

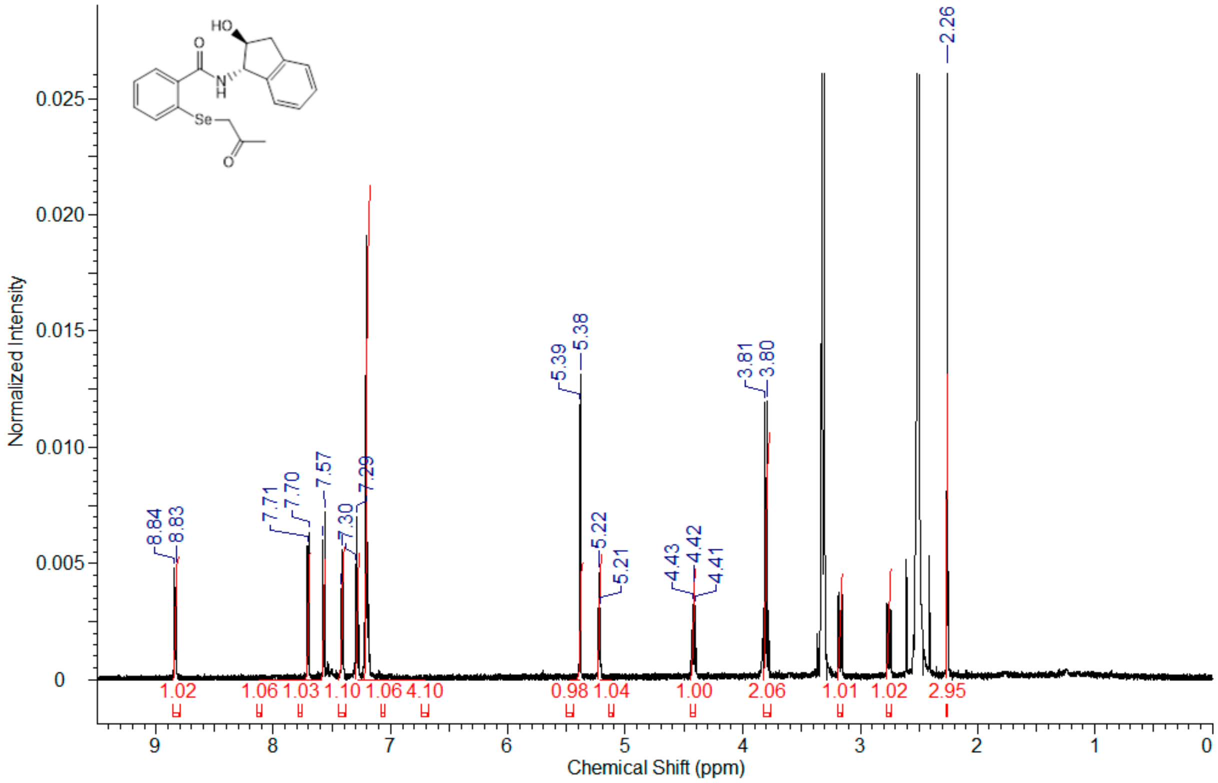

HO
O
N
H
Se
O
Normalized Intensity
0.025
0.020
0.015
0.010
0.005
0
8.84
8.83
7.71
7.70
7.57
7.30
7.29
5.39
5.38
5.22
5.21
4.43
4.42
4.41
3.81
3.80
2.26
1.02
1.06
1.03
1.10
1.06
4.10
0.98
1.04
1.00
2.06
1.01
1.02
2.95
9
8
7
6
5
4
3
2
1
0
Chemical Shift (ppm)

(a)

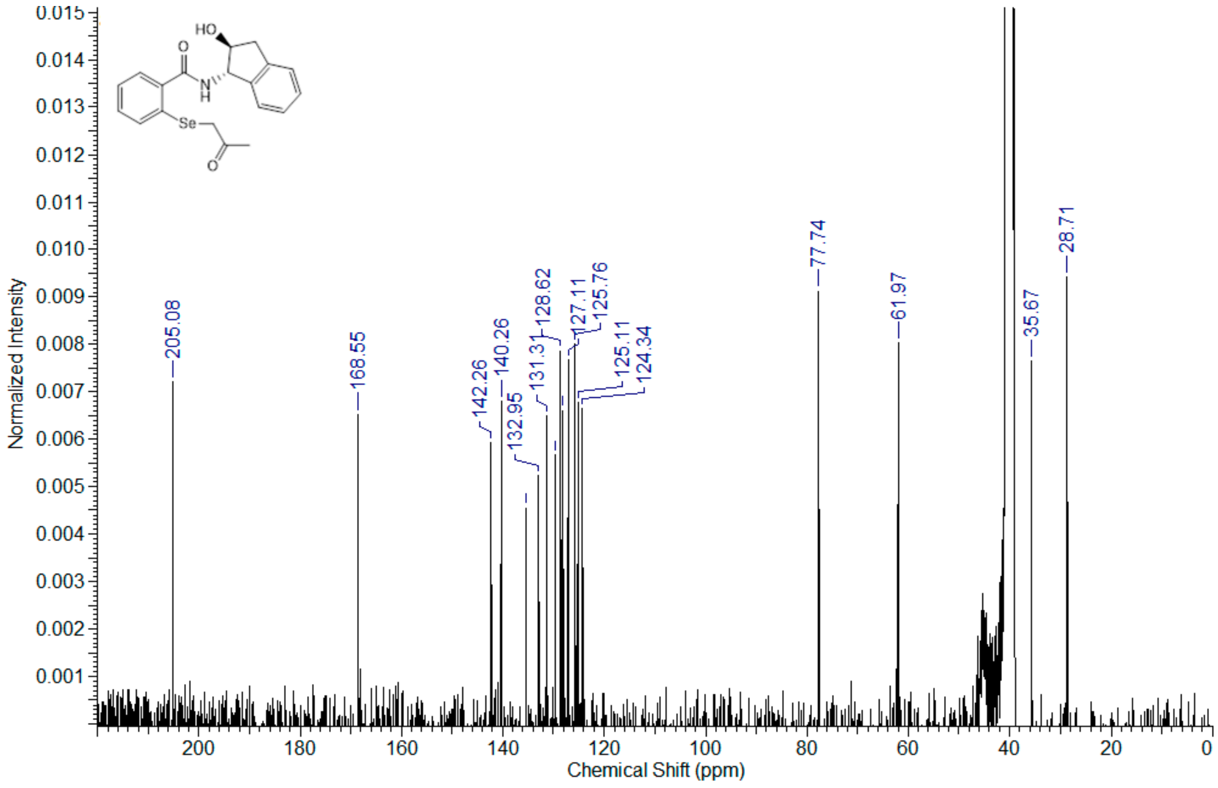

HO
O
N
H
Se
O
Normalized Intensity
0.015
0.014
0.013
0.012
0.011
0.010
0.009
0.008
0.007
0.006
0.005
0.004
0.003
0.002
0.001
205.08
168.55
142.26
140.26
132.95
131.31
128.62
127.11
125.76
125.11
124.34
77.74
61.97
35.67
28.71
200
180
160
140
120
100
80
60
40
20
0
Chemical Shift (ppm)

(b)

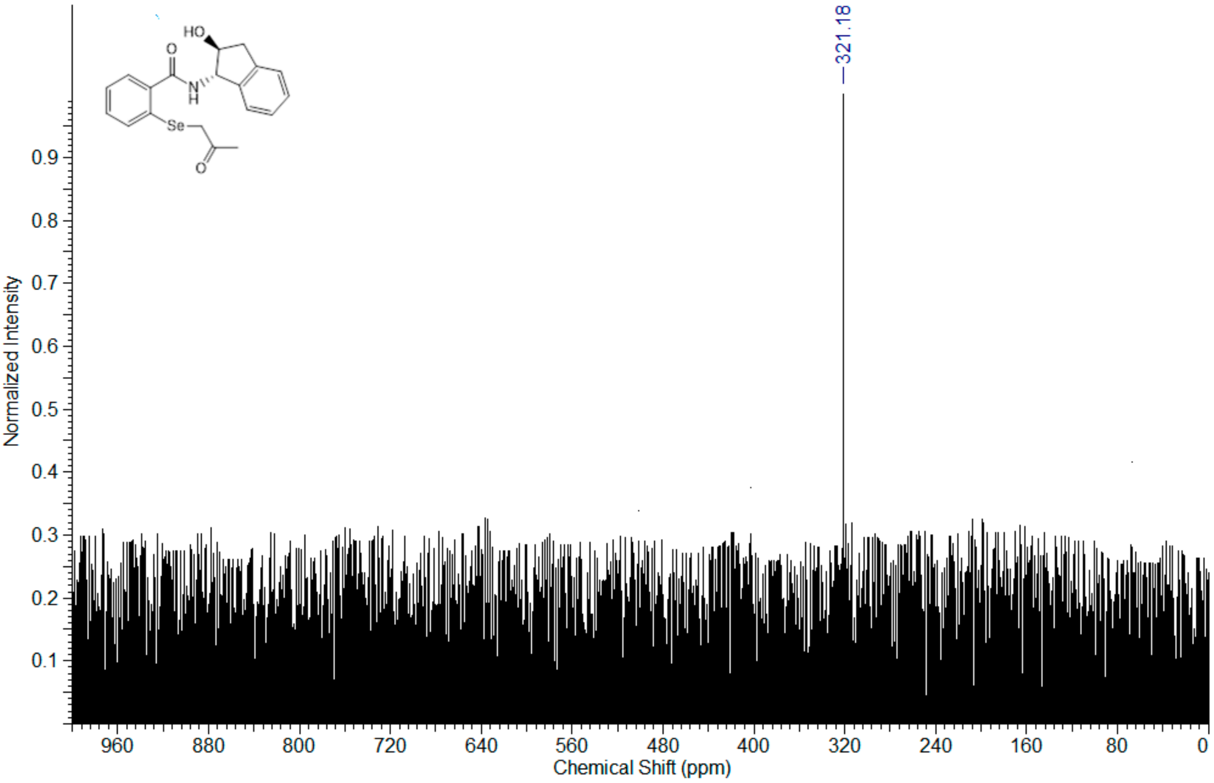

(c)

Figure S13. (**a**) 1H NMR, (**b**) 13C NMR, and (**c**) 77Se NMR spectra of *N*-((*1S,2S*)-(+)-*trans*-2-hydroksy-1-indanyl)- 2-((2-oxopropyl)selanyl)benzamide **23**.

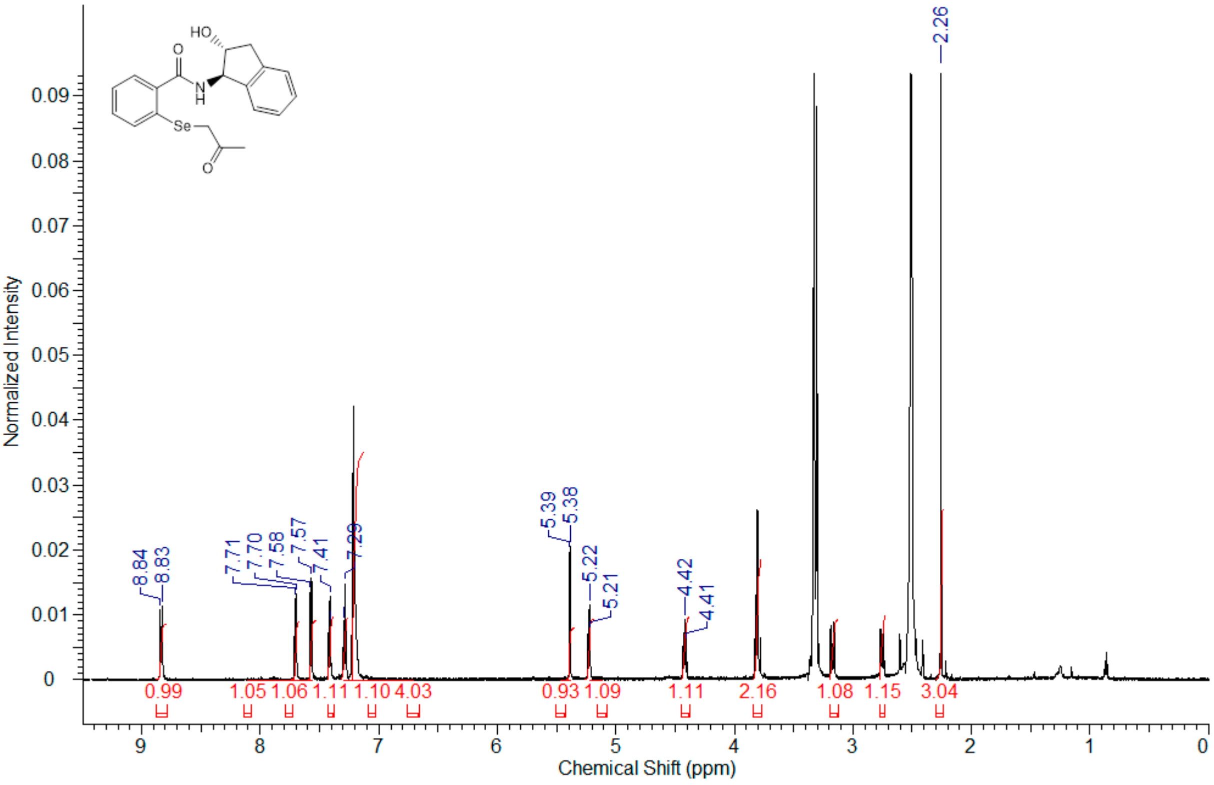

(a)

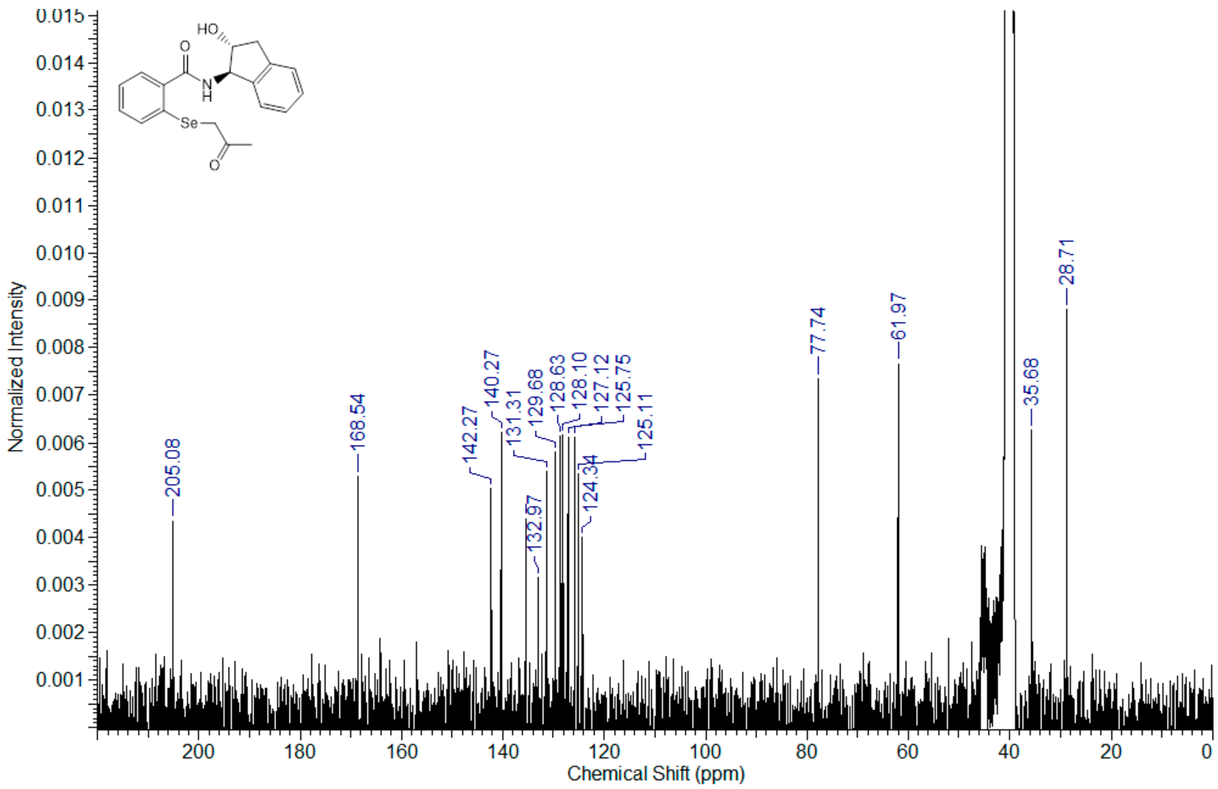

(b)

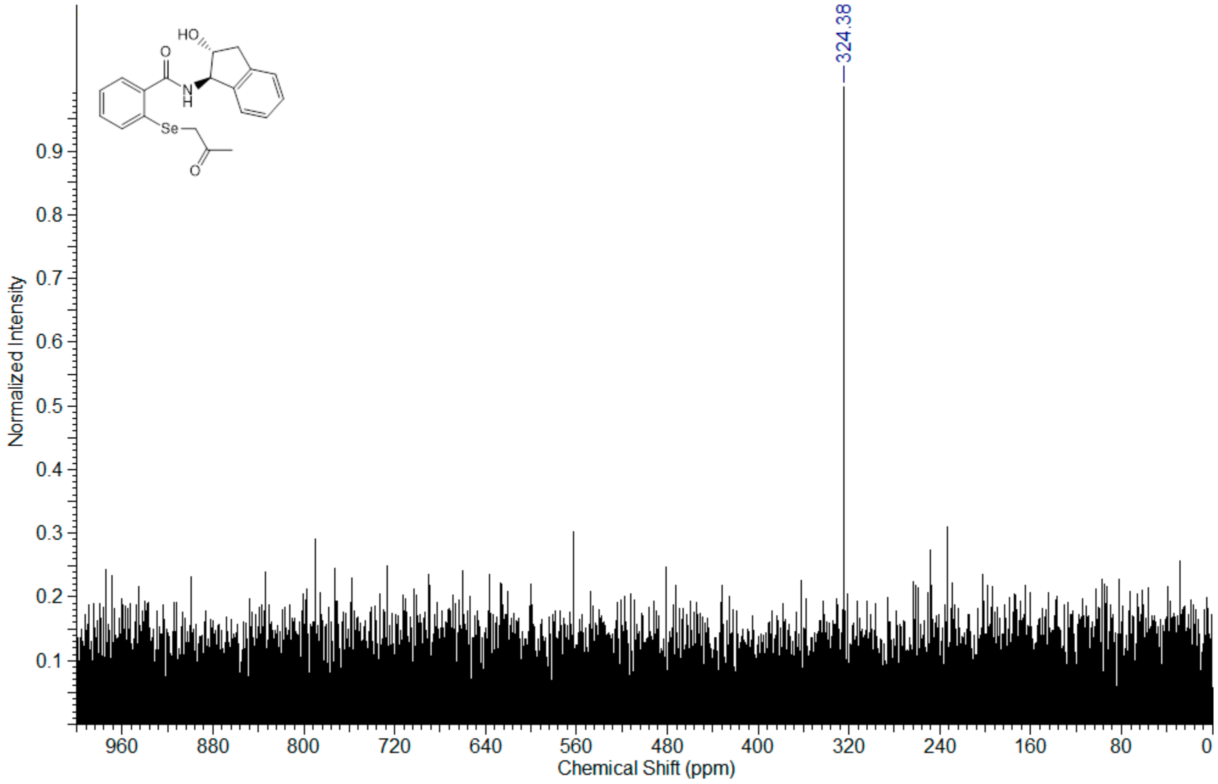

(c)

Figure S14. (**a**) 1H NMR, (**b**) 13C NMR, and (**c**) 77Se NMR spectra of *N*-((*1R,2R*)-(-)-*trans*-2-hydroksy-1-indanyl)- 2-((2-oxopropyl)selanyl)benzamide **24**.

2. HPLC analyses of selenides 11-24

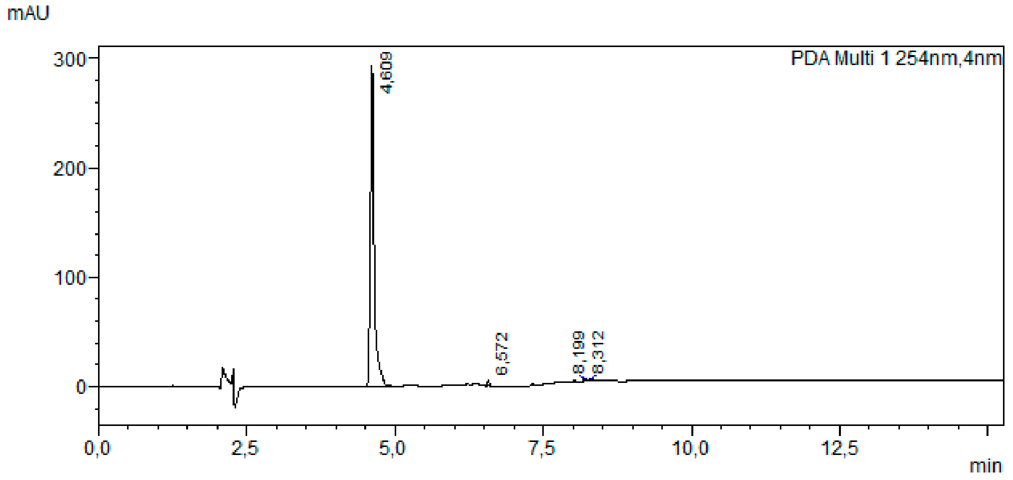

Peak Table

PDA Ch1 254nm

Peak#	Ret. Time	Area	Area%
1	4.609	1332186	98.252
2	6.572	8824	0.651
3	8.199	7521	0.555
4	8.312	7361	0.543
Total		1355892	100.000

Figure S15. Chromatograph of *N*-((*S*)-(+)-*sec*-butyl)- 2-((2-oxopropyl)selanyl)benzamide **11**.

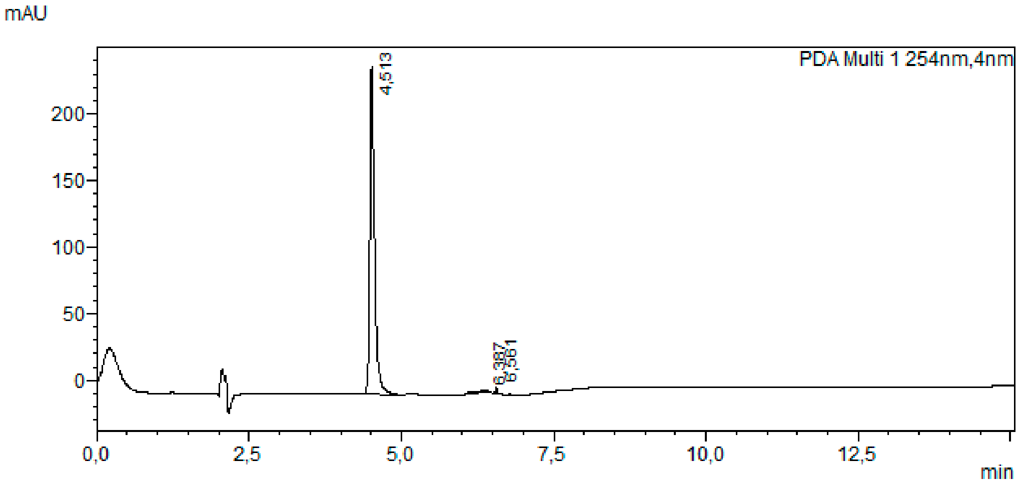

Peak Table

PDA Ch1 254nm

Peak#	Ret. Time	Area	Area%
1	4.513	1342761	97.757
2	6.387	20868	1.519
3	6.561	9944	0.724
Total		1373573	100.000

Figure S16. Chromatograph of *N*-((*R*)-(-)-*sec*-butyl)- 2-((2-oxopropyl)selanyl)benzamide **12**.

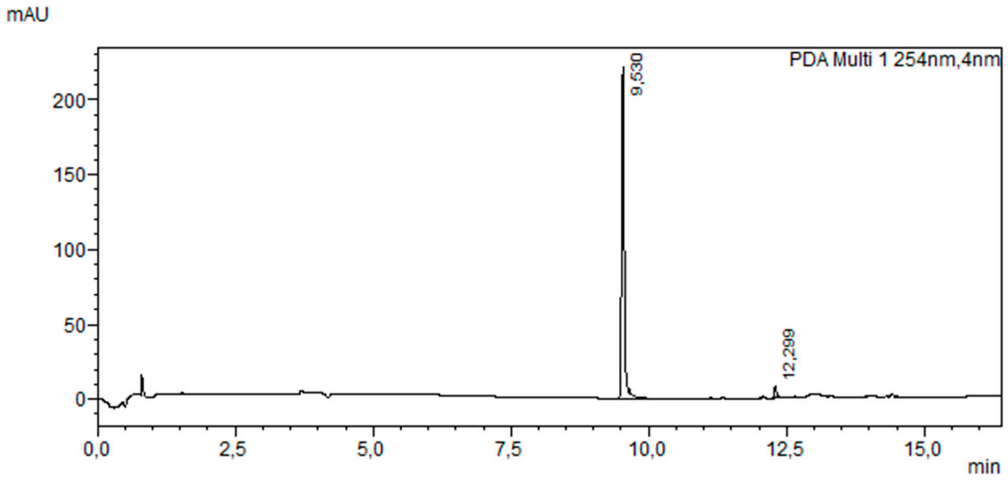

Peak Table

PDA Ch1 254nm

Peak#	Ret. Time	Area	Area%
1	9.530	891703	97.380
2	12.299	23989	2.620
Total		915692	100.000

Figure S17. Chromatograph of *N*-((*S*)-(+)-1-hydroksy-2-butanyl)- 2-((2-oxopropyl)selanyl)benzamide **13**.

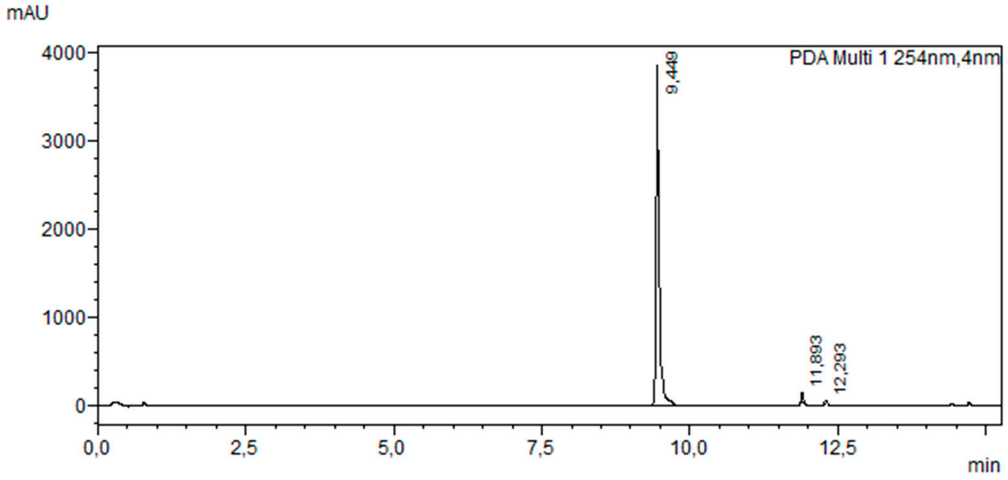

Peak Table

PDA Ch1 254nm

Peak#	Ret. Time	Area	Area%
1	9.449	14327609	97.407
2	11.893	287457	1.954
3	12.293	93968	0.639
Total		14709033	100.000

Figure S18. Chromatograph of *N*-((*R*)-(-)-1-hydroksy-2-butanyl)- 2-((2-oxopropyl)selanyl)benzamide **14**.

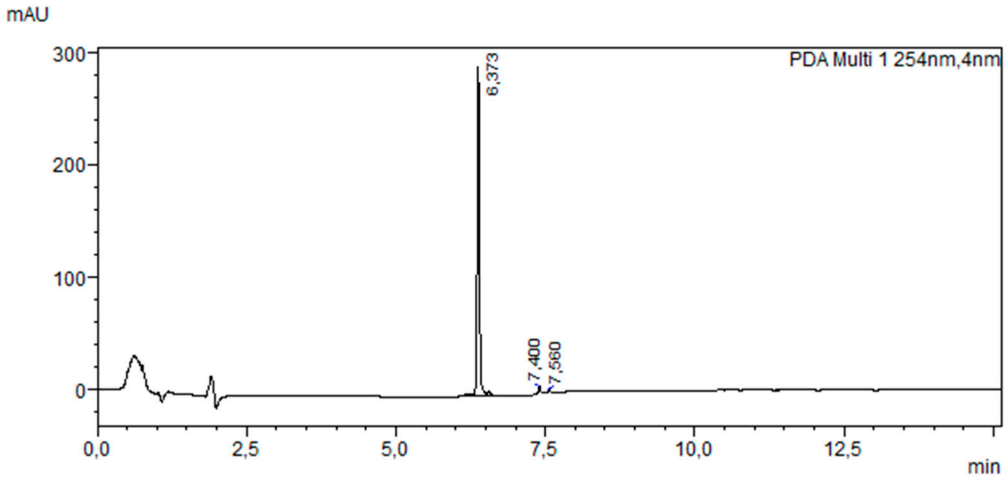

Peak Table

PDA Ch1 254nm

Peak#	Ret. Time	Area	Area%
1	6,373	837632	98,578
2	7,400	9905	1,166
3	7,560	2180	0,257
Total		849717	100,000

Figure S19. Chromatograph of *N*-((*R*)-(-)-1,2,3,4-tetrahydro-1-napthyl) 2-((2-oxopropyl)selanyl)benzamide **15**.

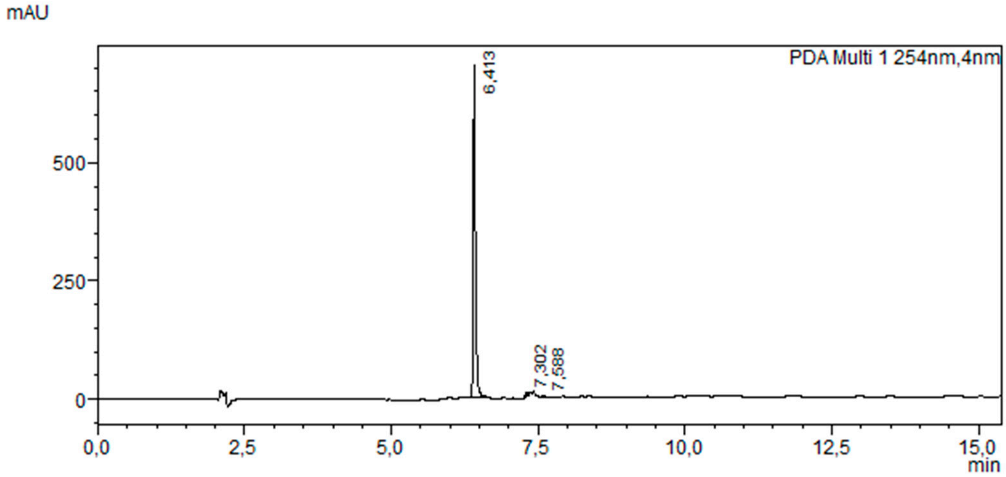

Peak Table

PDA Ch1 254nm

Peak#	Ret. Time	Area	Area%
1	6,413	1968918	98,553
2	7,302	15652	0,783
3	7,588	13263	0,664
Total		1997833	100,000

Figure S20. Chromatograph of *N*-((*S*)-(+)-1,2,3,4-tetrahydro-1-napthyl) 2-((2-oxopropyl)selanyl)benzamide **16**.

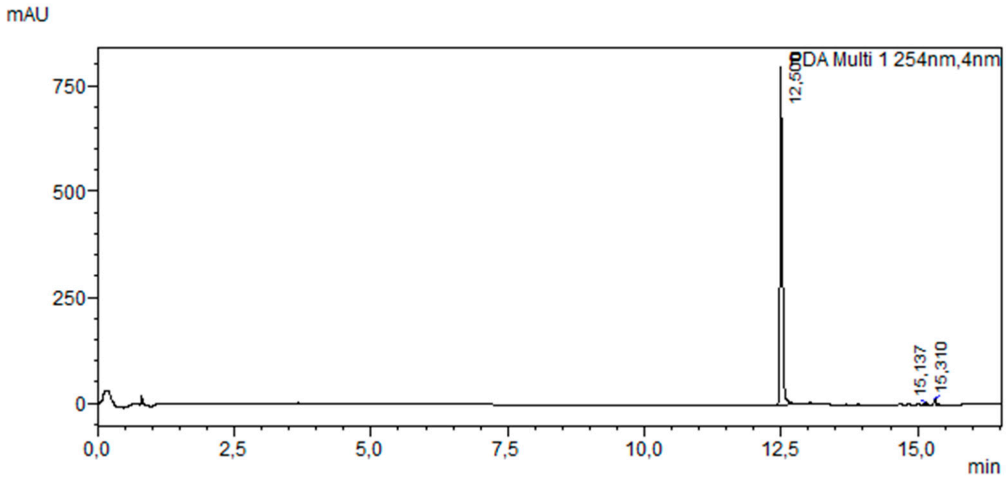

Peak Table

PDA Ch1 254nm

Peak#	Ret. Time	Area	Area%
1	12,500	2454696	98,801
2	15,137	18709	0,753
3	15,310	11081	0,446
Total		2484486	100,000

Figure S21. Chromatograph of *N*-((*R*)-(+)-α-metylbenzyl)-2-((2-oxopropyl)selanyl)benzamide **17**.

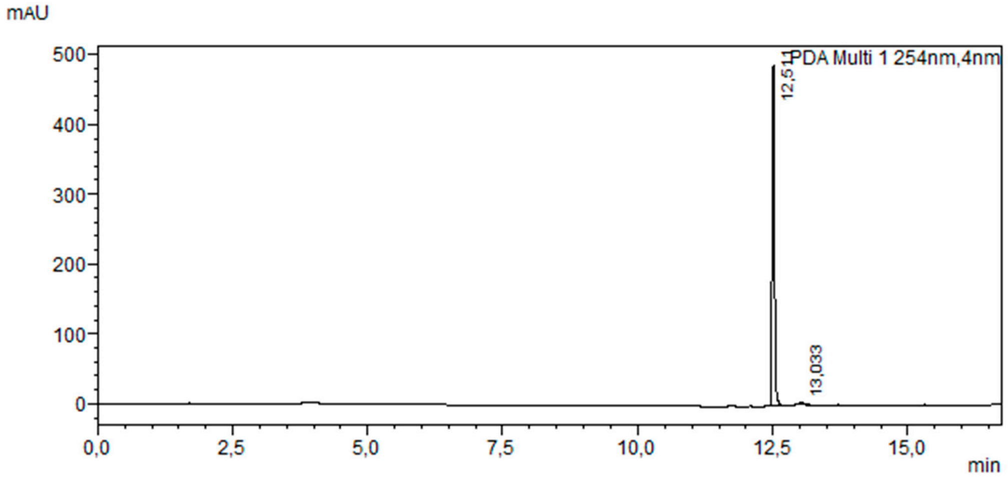

Peak Table

PDA Ch1 254nm

Peak#	Ret. Time	Area	Area%
1	12,511	1434673	99,076
2	13,033	13380	0,924
Total		1448053	100,000

Figure S22. Chromatograph of *N*-((*S*)-(-)-α-metylbenzyl)-2-((2-oxopropyl)selanyl)benzamide **18**.

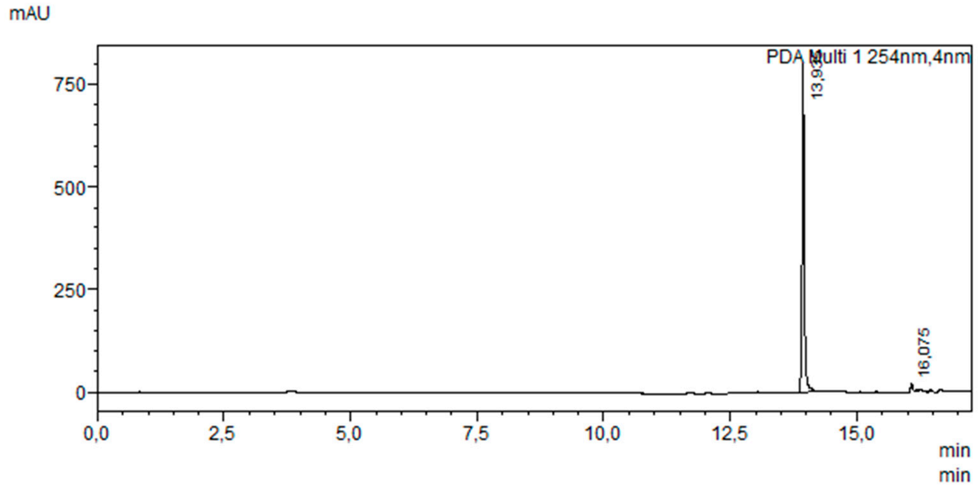

Peak Table

PDA Ch1 254nm

Peak#	Ret. Time	Area	Area%
1	13,935	2598181	98,974
2	16,075	26939	1,026
Total		2625120	100,000

Figure S23. Chromatograph of *N*-((*S*)-(-)-1-(1-napthyl)etyl)- 2-((2-oxopropyl)selanyl)benzamide **19**.

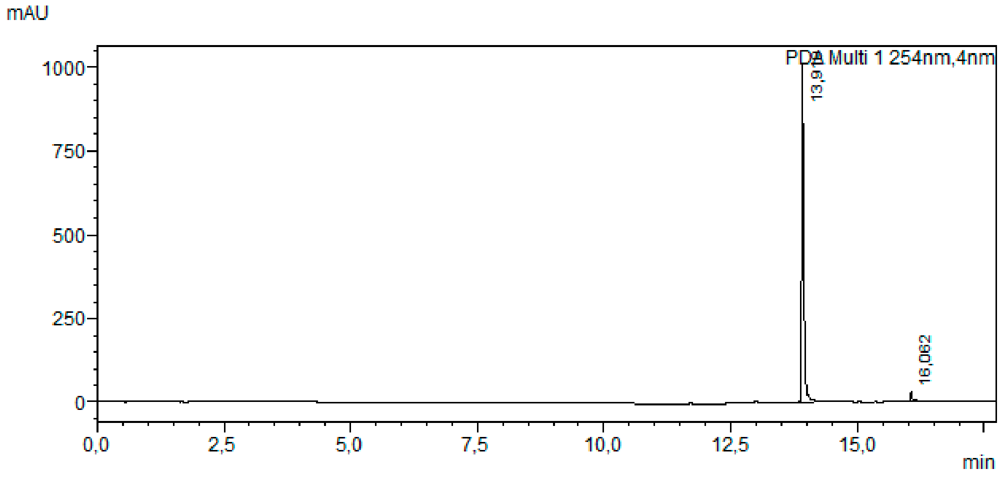

Peak Table

PDA Ch1 254nm

Peak#	Ret. Time	Area	Area%
1	13,919	3184362	98,031
2	16,062	63946	1,969
Total		3248308	100,000

Figure S24. Chromatograph of *N*-((*S*)-(-)-1-(1-napthyl)etyl)- 2-((2-oxopropyl)selanyl)benzamide **20**.

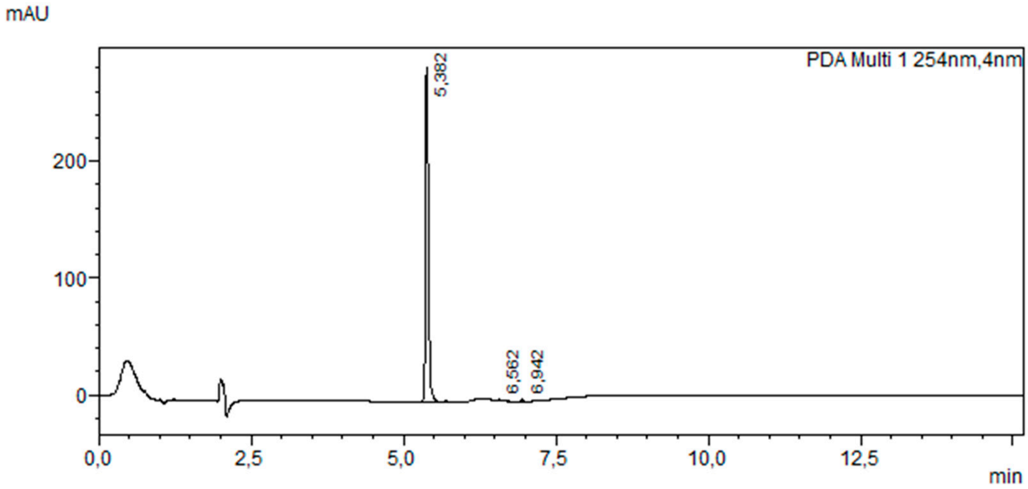

Peak Table

PDA Ch1 254nm

Peak#	Ret. Time	Area	Area%
1	5,382	895176	99,004
2	6,562	2353	0,260
3	6,942	6653	0,736
Total		904183	100,000

Figure S25. Chromatograph of *N*-((*1S,2R*)-(-)-*cis*-2-hydroksy-1-indanyl)-2-((2-oxopropyl)selanyl)benzamide **21**.

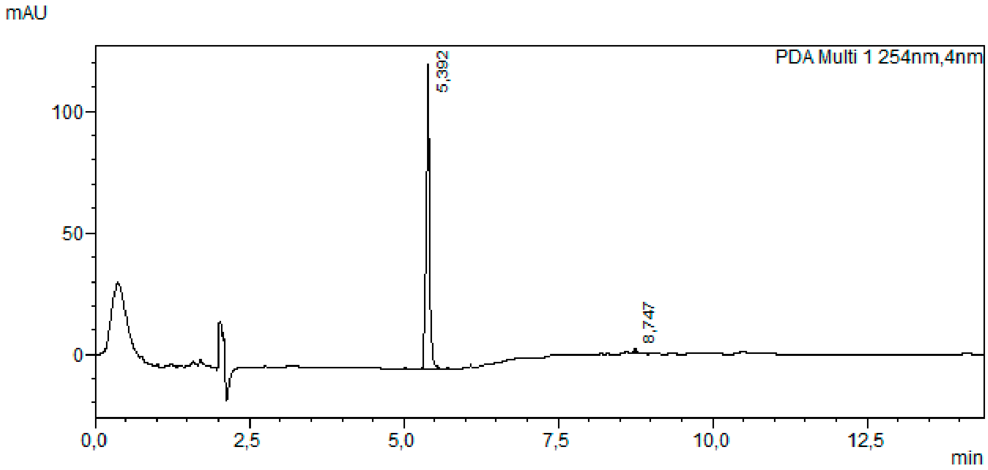

Peak Table

PDA Ch1 254nm

Peak#	Ret. Time	Area	Area%
1	5,392	454208	98,380
2	8,747	7481	1,620
Total		461689	100,000

Figure S26. Chromatograph of *N*-((*1S,2R*)-(-)-*cis*-2-hydroksy-1-indanyl)-2-((2-oxopropyl)selanyl)benzamide **22**.

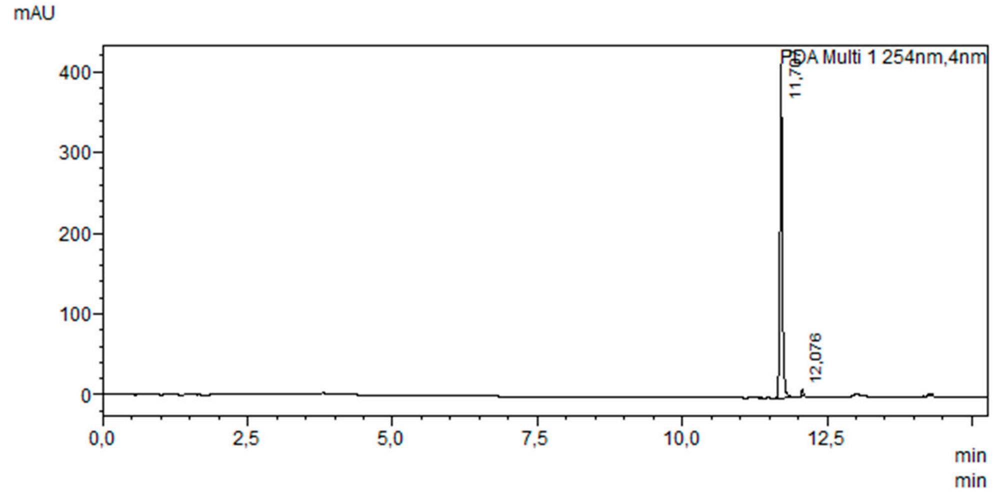

Peak Table

PDA Ch1 254nm

Peak#	Ret. Time	Area	Area%
1	11,707	1234832	98,252
2	12,076	21963	1,748
Total		1256796	100,000

Figure S27. Chromatograph of *N*-((*1S,2S*)-(+)-*trans*-2-hydroksy-1-indanyl)-2-((2-oxopropyl)selanyl)benzamide **23**.

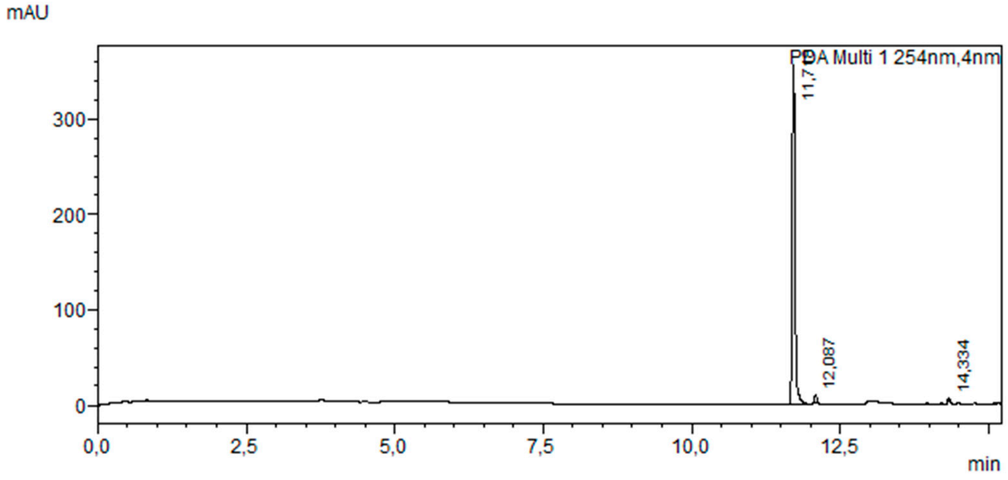

Peak Table

PDA Ch1 254nm

Peak#	Ret. Time	Area	Area%
1	11,718	1105112	97,421
2	12,087	20992	1,851
3	14,334	8268	0,729
Total		1134373	100,000

Figure S28. Chromatograph of *N*-((*1R,2R*)-(-)-*trans*-2-hydroksy-1-indanyl)-2-((2-oxopropyl)selanyl)benzamide **24**.

3. Antioxidant activity measurement

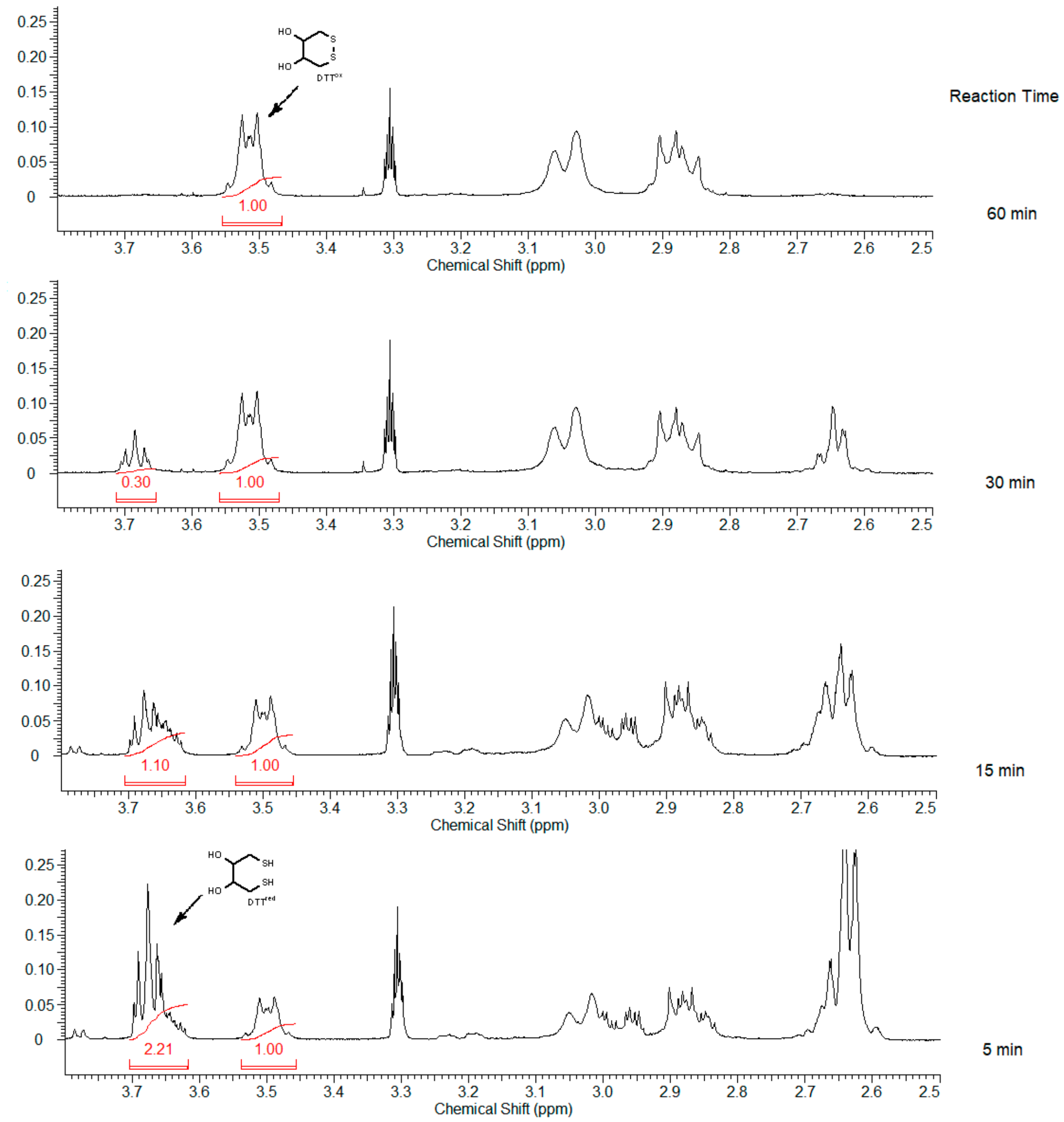

Figure S29. Example of ^{1}H NMR spectra for antioxidant Iwaoka test after reaction time 5 min, 15 min, 30 min, 60 min for compound 23.

	**5 min**			**15 min**		
Compound	Integration DTT^{red}	Integration DTT^{ox}	**Remaining DTT^{red}[%]**	Integration DTT^{red}	Integration DTT^{ox}	**Remaining DTT^{red}[%]**
**11/12**	5,301	1	84,13	1,945	1	66,04
**11/12**	2,751	1	73,34	1,293	1	56,39
**13/14**	3,843	1	79,35	2,06	1	67,32
**13/14**	1,827	1	64,63	1,231	1	55,18
**15/16**	5,999	1	85,71	3,536	1	77,95
**15/16**	5,886	1	85,48	3,618	1	78,35
**17/18**	12,895	1	92,80	8,566	1	89,55

**17/18**	7,569	1	88,33	9,945	1	90,86
**19/20**	13,786	1	93,24	8,491	1	89,46
**19/20**	13,322	1	93,02	9,703	1	90,66
**21/22**	3,136	1	75,82	1,077	1	51,85
**21/22**	2,205	1	68,80	1,096	1	52,29
**21/22**	2,684	1	72,86	1,105	1	52,49
**23/24**	3,844	1	79,36	1,052	1	51,27
**23/24**	3,721	1	78,82	1,622	1	61,86

Figure S30. Results of antioxidant activity measurement of integration from ^{1}H NMR spectra after reaction time 5 min and 15 min for all samples and compound 11-24.

	**5 min**			**15 min**		
Compound	Integration DTT^{red}	Integration DTT^{ox}	**Remaining DTT^{red}[%]**	Integration DTT^{red}	Integral DTT^{ox}	**Remaining DTT^{red}[%]**
**11/12**	0,705	1	41,35	0,197	1	16,46
**11/12**	0,886	1	46,98	0,1	1	9,09
**13/14**	1,104	1	52,47	0,307	1	23,49
**13/14**	0,823	1	45,15	0,25	1	20,00
**15/16**	2,273	1	69,45	1,267	1	55,89
**15/16**	2,245	1	69,18	1,322	1	56,93
**17/18**	4,373	1	81,39	2,733	1	73,21
**17/18**	5,331	1	84,20	2,869	1	74,15
**19/20**	4,904	1	83,06	2,422	1	70,78
**19/20**	5,509	1	84,64	2,468	1	71,16
**21/22**	0,451	1	31,08	0,134	1	11,82
**21/22**	0,301	1	23,14	0	1	0,00
**21/22**	0,375	1	27,27	0,079	1	7,32
**23/24**	0,368	1	26,90	0,09	1	8,26
**23/24**	0,502	1	33,42	0,15	1	13,04

Figure S31. Results of antioxidant activity measurement of integration from ^{1}H NMR spectra after reaction time 30 min and 60 min for all samples and compound 11-24.

4. DPPH test calibration curves

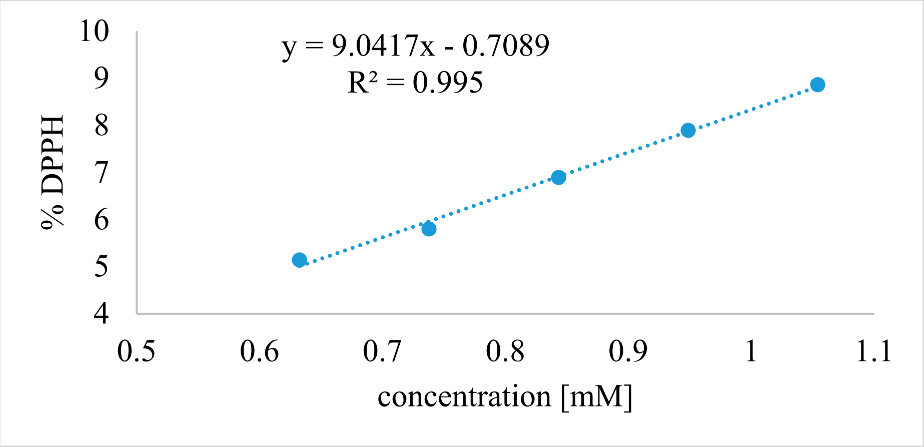

Figure S32. Compound 11/12—calibration curve.

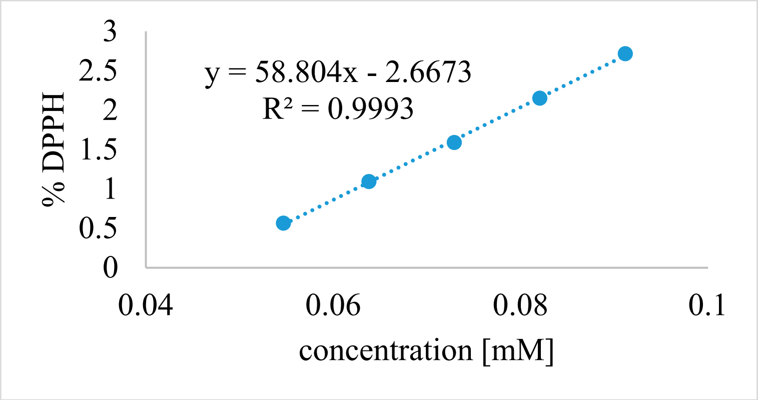

Figure S33. Compound 13/14—calibration curve.

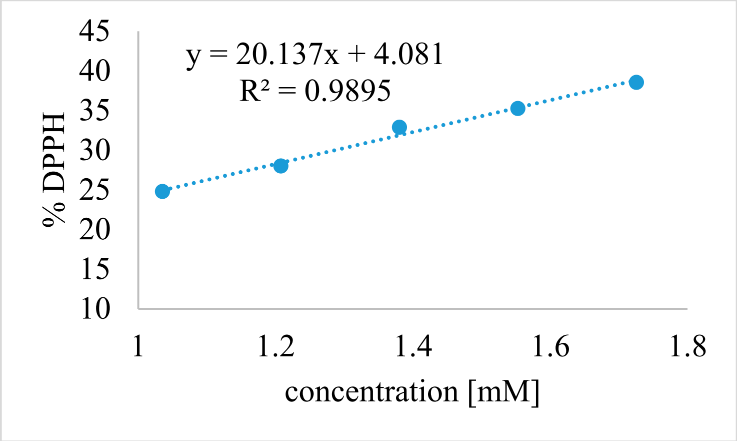

Figure S34. Compound 15/16—calibration curve.

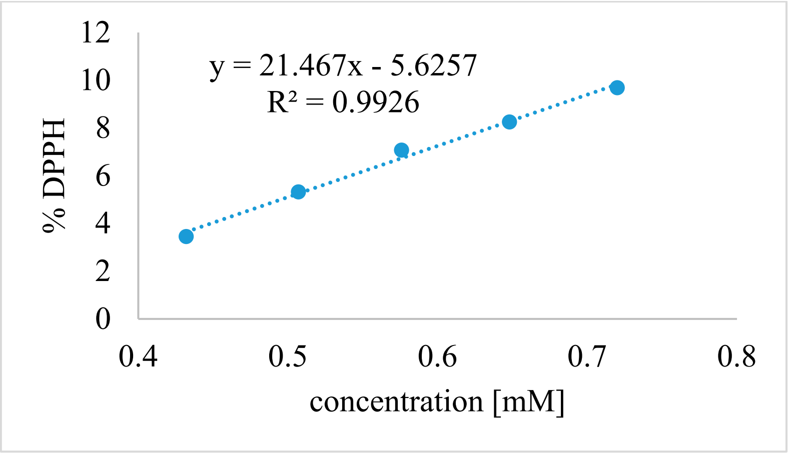

Figure S35. Compound 17/18—calibration curve.

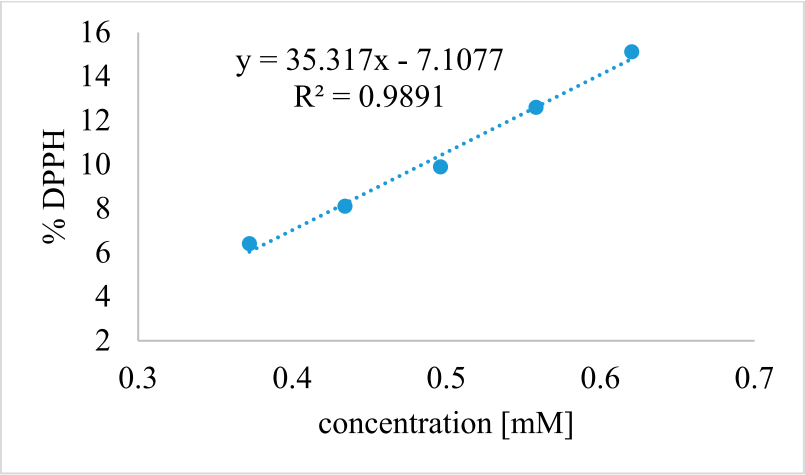

Figure S36. Compound 19/20—calibration curve.

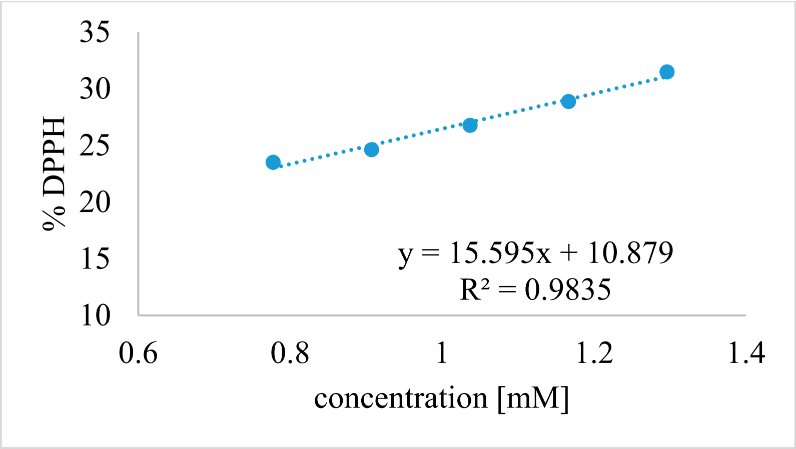

Figure S37. Compound 21/22—calibration curve.

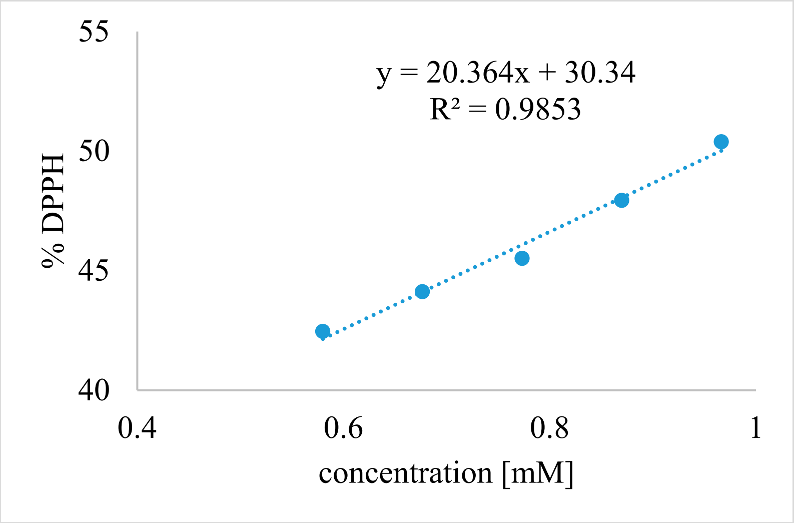

Figure S38. Compound 23/24—calibration curve.

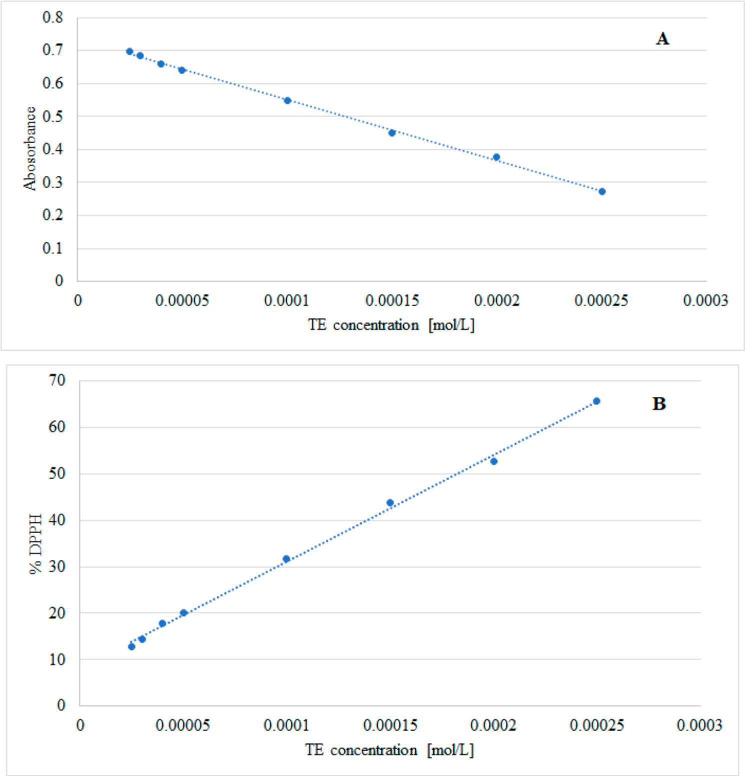

Concentration range	$0.025 \cdot 10^{-3}$ -$0.25 \cdot 10^{-3}$ M
slope	304119
intercept	0.7493
R^2	0.998

Figure S39. Standard curves for the relationship: absorbance = function (concentration TE) (A), % DPPH = function (concentration of TE) (B).
